# The GRK2/AP‐1 Signaling Axis Mediates Vascular Endothelial Dysfunction and Atherosclerosis Induced by Oscillatory Low Shear Stress

**DOI:** 10.1002/advs.202501981

**Published:** 2025-06-10

**Authors:** Li‐Da Wu, Yi Shi, Chao‐Hua Kong, Ai‐Qun Chen, Ying Kang, Jun‐Yan Kan, Xiao‐Min Jiang, Peng Chu, Dong‐Chen Wang, Yi‐Fei Lv, Zhi‐Yuan Qian, Zi‐Hao Jiang, Yun‐Wei Chen, Yue Sun, Rui‐Rui Chang, Wen‐Ying Zhou, Yue Gu, Jun‐Xia Zhang, Shao‐Liang Chen

**Affiliations:** ^1^ Department of Cardiology Nanjing First Hospital Nanjing Medical University Nanjing 210000 China; ^2^ College of Pharmacy Nanjing Medical University Nanjing 210000 China

**Keywords:** activator protein 1, atherosclerosis, G protein‐coupled receptor kinase 2, shear stress, vascular endothelial dysfunction

## Abstract

Disturbed blood flow and the resulting oscillatory low shear stress (OSS) are key contributors to vascular endothelial dysfunction and the initiation of atherosclerosis. However, the molecular mediators that translate abnormal hemodynamic signals into pathological vascular endothelial responses remain unclear. G protein‐coupled receptors (GPCRs) are classical mechanosensors in the vascular endothelium. Here, using vascular endothelial‐specific knockout mice, in vitro parallel plate flow chamber systems, and phosphoproteomic analysis, G protein‐coupled receptor kinase 2 (GRK2) is identified as a central mediator of OSS‐induced vascular endothelial dysfunction. Mechanistically, OSS promotes GRK2 phosphorylation at serine 29, which subsequently activates the transcription factor activator protein‐1 (AP‐1), increasing the expression of the proinflammatory adhesion molecules intercellular cell adhesion molecule‐1 (ICAM1) and vascular cell adhesion molecule 1 (VCAM1). In parallel, AP‐1 promotes nuclear receptor subfamily 4 group A 1 (NR4A1) transcription, which anchors liver kinase B1 (LKB1) to the nucleus and suppresses downstream AMP‐activated protein kinase (AMPK) signaling, leading to metabolic dysregulation and impaired vascular endothelial homeostasis. These findings underscore the GRK2/AP‐1 signaling axis as a crucial mechanotransduction cascade linking disturbed flow to vascular endothelial dysfunction. Given the important role of GPCRs in mechanotransduction, targeting GRK2 may offer a novel therapeutic approach for atherosclerosis.

## Graphical abstract

This study highlights GRK2 is a central mediator in OSS‐induced endothelial dysfunction. OSS activates GPCRs in endothelial cells, leading to GRK2 phosphorylation and the activation of AP‐1. AP‐1 induces inflammation, while also promoting NR4A1 expression and anchoring LKB1 in the nucleus, which suppresses AMPK activity. This cascade causes endothelial dysfunction and atherosclerosis.

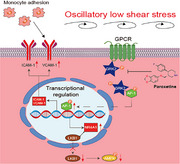

## 1 Introduction

Atherosclerosis is a leading cause of mortality worldwide and a significant global health challenge.^[^ ^1^ ^]^ Vascular endothelial cells, which line the interior surface of blood vessels, are directly exposed to hemodynamic forces from blood flow, and their dysfunction is closely linked to the development of atherosclerosis.^[^ ^2^ ^]^ Shear stress, a tangential force parallel to the vessel wall caused by the friction of blood flow, is a critical mechanical stimulus for vascular endothelial function.^[^ ^3^ ^]^ The magnitude of shear stress is positively associated with blood viscosity and inversely correlated with vascular diameter. In straight segments of blood vessels, pulsatile blood flow induces laminar shear stress (LSS), which generally protects the health and function of the endothelium. However, in vascular bifurcations and curved regions, disturbed flow leads to oscillatory low shear stress (OSS), which predisposes these areas to atherosclerotic plaque formation.^[^ ^4^ ^]^ Inflammation and energy metabolic dysfunction are key contributors to vascular endothelial dysfunction and atherogenesis. Despite extensive research, the molecular mechanisms by which OSS induces inflammation and energy metabolic dysfunction remain incompletely understood.^[^ ^5^ ^]^

G protein‐coupled receptors (GPCRs) are present on the surface of nearly all living cells and play an essential role in transmitting extracellular signals.^[^ ^6^ ^]^ Changes in the extracellular mechanical environment, including stretching or pressure, can activate GPCRs. G protein‐coupled receptor kinase 2 (GRK2), a member of the GRKs family, is primarily responsible for regulating GPCR signal transduction.^[^ ^7^ ^]^ Mechanical signals can activate GPCRs, triggering GRK2 phosphorylation and the recruitment of β‐arrestin, which terminates GPCR signaling. In addition to its canonical role in GPCR desensitization, GRK2 can catalyze the phosphorylation of various substrates, contributing to broader cellular signaling processes.^[^ ^8^ ^]^ GRK2 has emerged as a key player in cellular signal transduction and cardiovascular diseases.^[^ ^9^ ^]^ However, whether GRK2 plays a role in OSS‐induced endothelial inflammation and energy metabolism dysfunction is unknown.

Activator protein‐1 (AP‐1) is a transcription factor that plays a multifaceted role in inflammation. First, it regulates the proliferation and survival of inflammatory cells, influencing the magnitude and duration of inflammatory responses.^[^ ^10^ ^]^ Second, it regulates the expression of numerous inflammation‐related genes,^[^ ^11^ ^]^ including cytokines (e.g., tumor necrosis factor‐alpha (TNF‐α) and interleukin‐1 (IL‐1)), inflammatory mediators (e.g., nitric oxide synthase (NOS) and cyclooxygenase (COX)), and cell adhesion molecules (e.g., intercellular adhesion molecule 1 (ICAM1) and vascular cell adhesion molecule 1 (VCAM1)),^[^ ^12^, ^13^, ^14^ ^]^ and promotes their release, exacerbating the inflammatory cascade.^[^ ^15^ ^]^ The existence of an inflammatory regulatory circuit between AP‐1 and GRK2 as remains underreported.

This study utilized large‐scale phosphoproteomics, transgenic mice, and numerous molecular biology techniques to illustrate that OSS increases the level of GRK2 phosphorylated at serine 29 (GRK2^S29p^) in vascular endothelial cells, thereby promoting AP‐1 phosphorylation and contributing to atherosclerosis. Our findings suggest that GRK2^S29p^ represents a potential therapeutic target for the treatment of atherosclerosis, offering new insights into the mechanistic link between hemodynamic forces and vascular diseases.

## 2 Results

### 2.1 OSS Increases the Level of GRK2^S29p^ in Vascular Endothelial Cells

**Figure 1:**
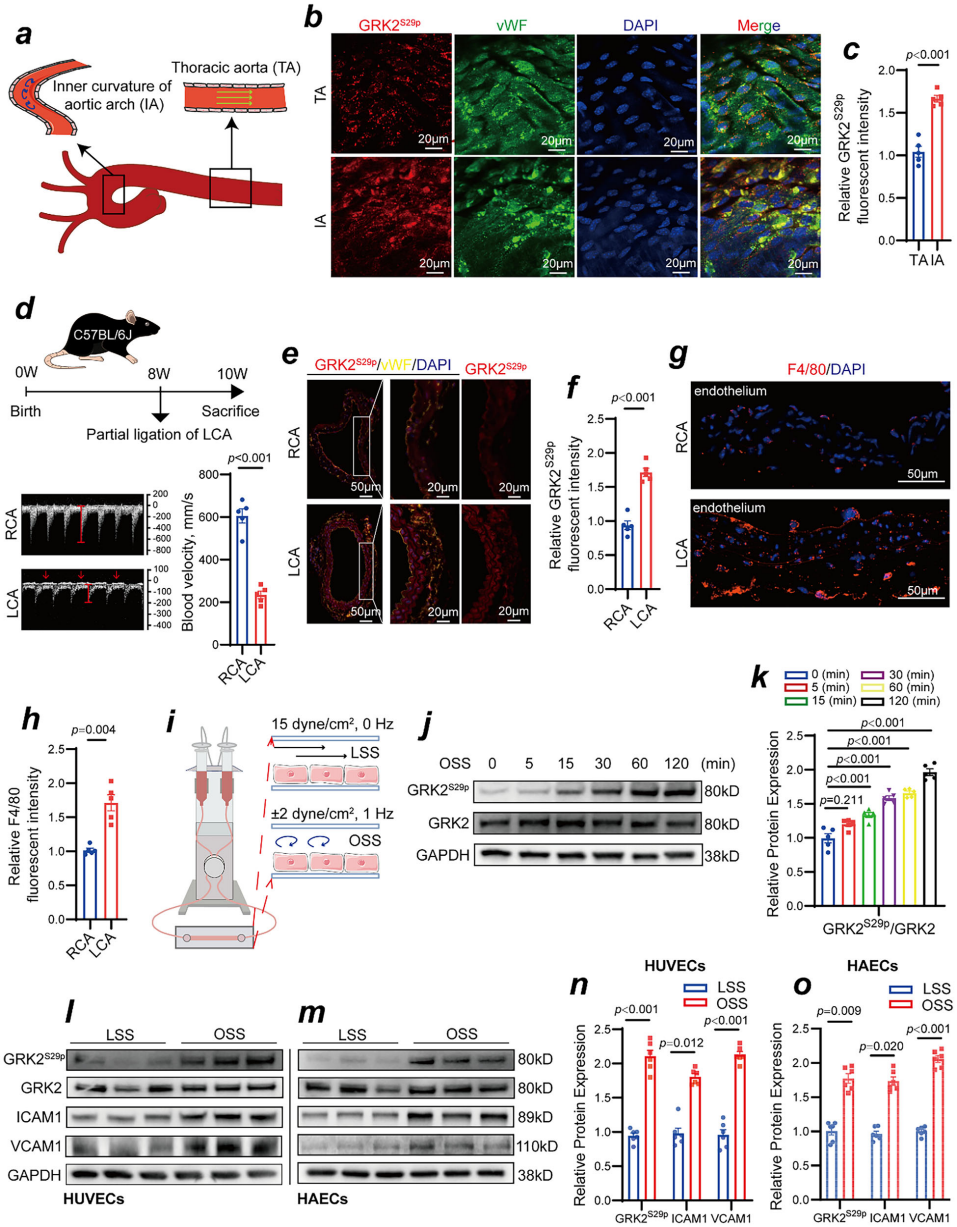
GRK2 activity is elevated in vascular endothelial cells exposed to disturbed flow. a) Schematic illustrating blood flow in the thoracic aorta and the inner curvature of the aortic arch. b) *Enface* immunofluorescence staining of GRK2^S29p^ (red), von Willebrand factor (vWF, endothelial marker, green), and DAPI (blue) in the endothelial cells of the thoracic aorta and the inner curvature of the aortic arch isolated from C57BL/6J mice. c) Quantification of the relative GRK2^S29p^ fluorescent intensity (n = 5, Student's *t* test). d) Schematic figure of the construction of a partial LCA ligation animal model to simulate disturbed flow and OSS in vivo. High‐resolution Doppler ultrasound imaging confirmed the successful model establishment, with significantly reduced flow velocity and evidence of diastolic flow reversal in the ligated LCA. Red arrows highlight diastolic flow reversal. The right carotid artery (RCA) served as an internal control (n = 5, Student's *t* test). e) Immunofluorescence staining for GRK2^S29p^ (red), vWF (yellow), and DAPI (blue) in the RCA endothelium under laminar flow and the LCA endothelium under disturbed flow. f) Quantification of the relative GRK2^S29p^ fluorescent intensity (n = 5, Student's *t* test). g) Immunofluorescence staining for F4/80 (red) and DAPI (blue) reflecting the infiltration of macrophages in the RCA and the LCA. h) Quantification of the relative F4/80 fluorescent intensity (n = 5, Student's *t* test). i) Schematic figure of the in vitro parallel plate flow chamber system, in which endothelial cells were under LSS (15 dyne cm^−^^2^, 0 Hz) and OSS (±2 dyne cm^−^^2^, 1 Hz) conditions. j) HUVECs were subjected to OSS for 0, 5, 15, 30, 60, or 120 min. The proteins in the cell lysate were analyzed via Western blotting with the indicated antibodies. k) Quantification of GRK2^S29p^ expression levels (n = 5, one‐way ANOVA). l–o) GRK2^S29p^ expression was elevated in HUVECs (l, n) and HAECs (m, o), as was the expression of ICAM1 and VCAM1 under OSS conditions compared with LSS conditions.

As illustrated in **Figure** **1**a, blood flow in the thoracic aorta (TA) is laminar flow, subjecting the endothelium to LSS; in contrast, flow on the inner curvature of the aortic arch (IA) is disturbed flow, exposing the endothelium to OSS. Our study employed *enface* immunofluorescence staining of vascular endothelial cells from thoracic aortas and the inner curvature of aortic arches isolated from C57BL/6J mice. The results indicated that GRK2^S29p^ levels were elevated in vascular endothelial cells from the OSS region (inner curvature of the aortic arch) compared with those from the LSS region (thoracic aorta) (Figure 1b,c). Figure 1d shows a schematic diagram of the in vivo disturbed flow model used in this study, which was established by partial ligation of the left common carotid artery (LCA) according to the standard protocol described by Nam et al.^[^ ^16^ ^]^ Figure S1, Supporting Information illustrates the procedure, which involved ligating three of four LCA caudal branches, the external carotid artery (ECA), internal carotid artery (ICA), and occipital artery (OA), while leaving the superior thyroid artery (STA) patent to maintain partial blood flow. To confirm the successful induction of disturbed flow with OSS, we performed high‐resolution Doppler ultrasound imaging, which clearly demonstrated decreased flow velocity and diastolic flow reversal (red arrows) in the LCA (Figure 1d). Immunofluorescence revealed that the level of GRK2^S29p^ in the endothelium of the LCA subjected to OSS was significantly greater than that in the RCA (Figure 1e,f). Additionally, F4/80 immunofluorescence staining revealed that the degree of macrophage infiltration in the LCA under OSS was significantly greater than that in the RCA (Figure 1g,h). A parallel plate flow chamber system was also used in this study to apply different shear stresses to vascular endothelial cells in vitro. Figure 1i shows a schematic of the parallel plate flow chamber system. After exposing human umbilical vein endothelial cells (HUVECs) to OSS for different periods of time (±2 dyne cm^−2^, 1 Hz, 0, 5, 15, 30, 60, and 120 min), the Western blot results revealed that the GRK2^S29p^ levels increased with prolonged OSS exposure, whereas the overall expression level of GRK2 remained unchanged (Figure 1j,k). Following in vitro exposure of HUVECs to LSS (15 dyne cm^−^^2^, 0 Hz) or OSS (±2 dyne cm^−^^2^, 1 Hz) for 2 h, OSS markedly increased GRK2^S29p^ levels compared with LSS (Figure 1l,n). To further investigate the impact of OSS on GRK2^S29p^ in human aortic endothelial cells (HAECs), HAECs were subjected to LSS or OSS for 2 h, and the results were consistent with those in HUVECs (Figure 1m,o). Notably, we also examined the phosphorylation levels of other GRK2 sites and found that GRK2^T86p^, GRK2^T13p^, GRK2^S685p^, and GRK2^S670p^ levels remained unchanged (Figure S2, Supporting Information). Furthermore, following OSS exposure, the increase in GRK2^S29p^ levels was accompanied by significant upregulation of the inflammatory adhesion molecules ICAM1 and VCAM1 in HUVECs and HAECs (Figure 1l–o). The immunofluorescence results for ICAM1 and VCAM1 also indicated that OSS could lead to increased expression levels of ICAM1 and VCAM1 (Figure S3, Supporting Information).

### 2.2 GRK2^S29p^ Expression was Increased in Vascular Endothelial Cells at Atherosclerotic Plaques

**Figure 2:**
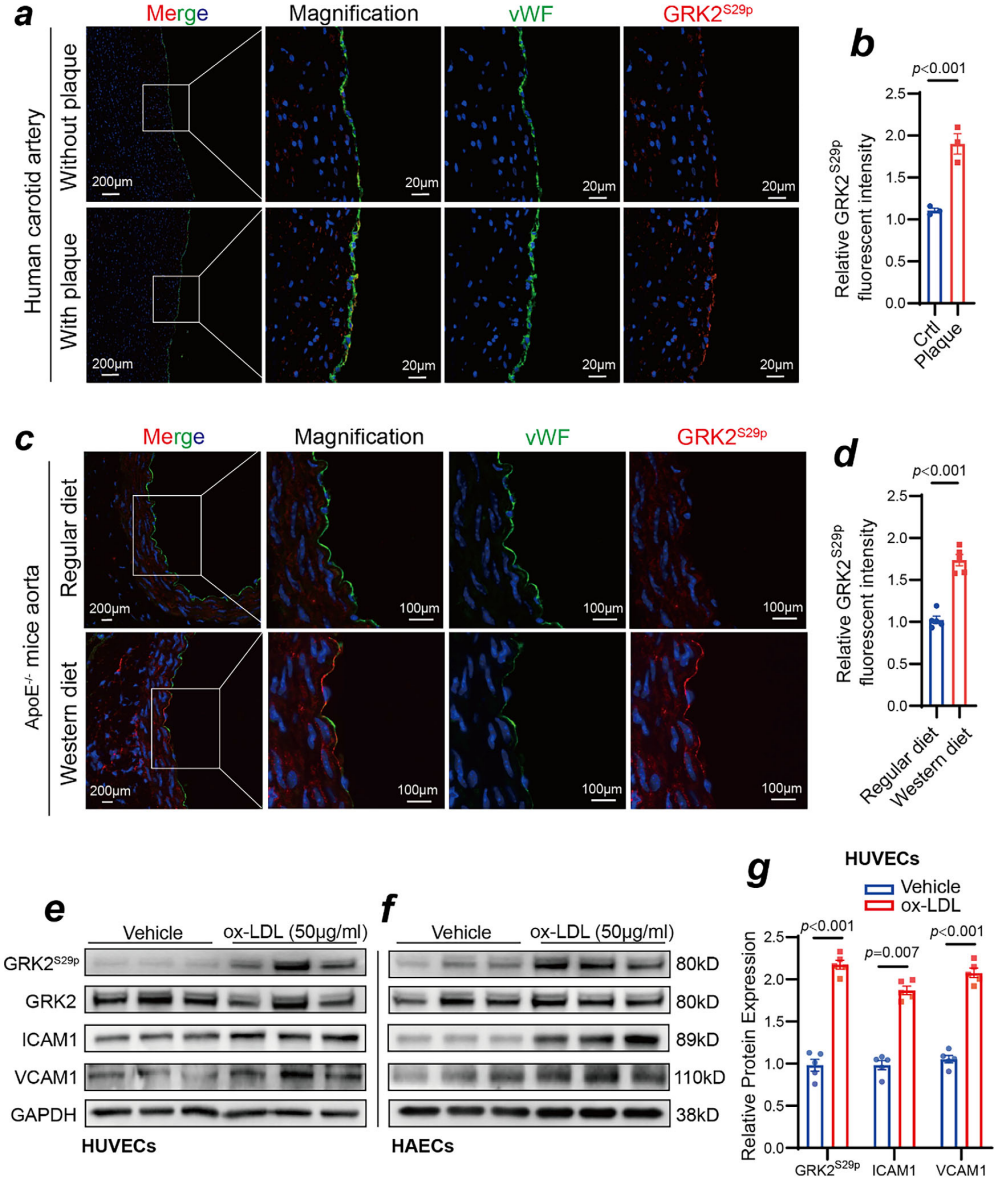
Elevated GRK2^S29p^ expression in the endothelium of atherosclerotic lesions in human carotid arteries and aortic arteries of ApoE^‐/‐^ hyperlipidemic mice. a) Immunofluorescence staining of GRK2^S29p^ (red), vWF (green), and DAPI (blue) in human carotid arteries with or without atherosclerotic plaques. b) Quantification of the relative GRK2^S29p^ fluorescent intensity (n = 3, Student's *t* test). c) Immunofluorescence staining for GRK2^S29p^ (red), vWF (green), and DAPI (blue) in aortas isolated from ApoE^−/−^ mice fed a normal diet and those fed a high‐fat Western diet. d) Quantification of the relative GRK2^S29p^ fluorescent intensity (n = 5, Student's *t* test). e,f) HUVECs (e) and HAECs (f) were treated with oxidized low‐density lipoprotein (x‐LDL, 50 µg mL^−1^ for 48 h), and the proteins in the cell lysate were evaluated via Western blotting with the indicated antibodies. g) Quantification of GRK2^S29p^, ICAM1, and VCAM1 protein expression levels (n = 5, Student's *t* test).

Vascular endothelial dysfunction is the initial step in atherosclerosis. In this study, in accordance with ethical guidelines, we performed immunofluorescence staining of GRK2^S29p^ in human carotid arteries with or without atherosclerotic plaques. The results revealed that GRK2^S29p^ expression was elevated in the endothelium of atherosclerotic lesions (**Figure** **2**a,b). We also developed an atherosclerotic animal model in which Apolipoprotein E knock (ApoE^‐/‐^) mice were fed a high‐fat Western diet for 12 weeks. Immunofluorescence staining revealed that GRK2^S29p^ expression was greater in atherosclerotic plaques than in nonatherosclerotic plaques in mice (Figure 2c,d). In vitro, we stimulated HUVECs and HAECs with ox‐LDL (50 µg mL^−1^). The results revealed that after ox‐LDL administration, the phosphorylation level of GRK2^S29p^, as well as the expression levels of the inflammatory adhesion molecules ICAM1 and VCAM1, were significantly elevated (Figure 2e–g; Figure S4, Supporting Information).

### 2.3 GRK2 May Be a Potential Target for Treating OSS‐Induced Inflammation in the Vascular Endothelium and Atherosclerosis

**Figure 3:**
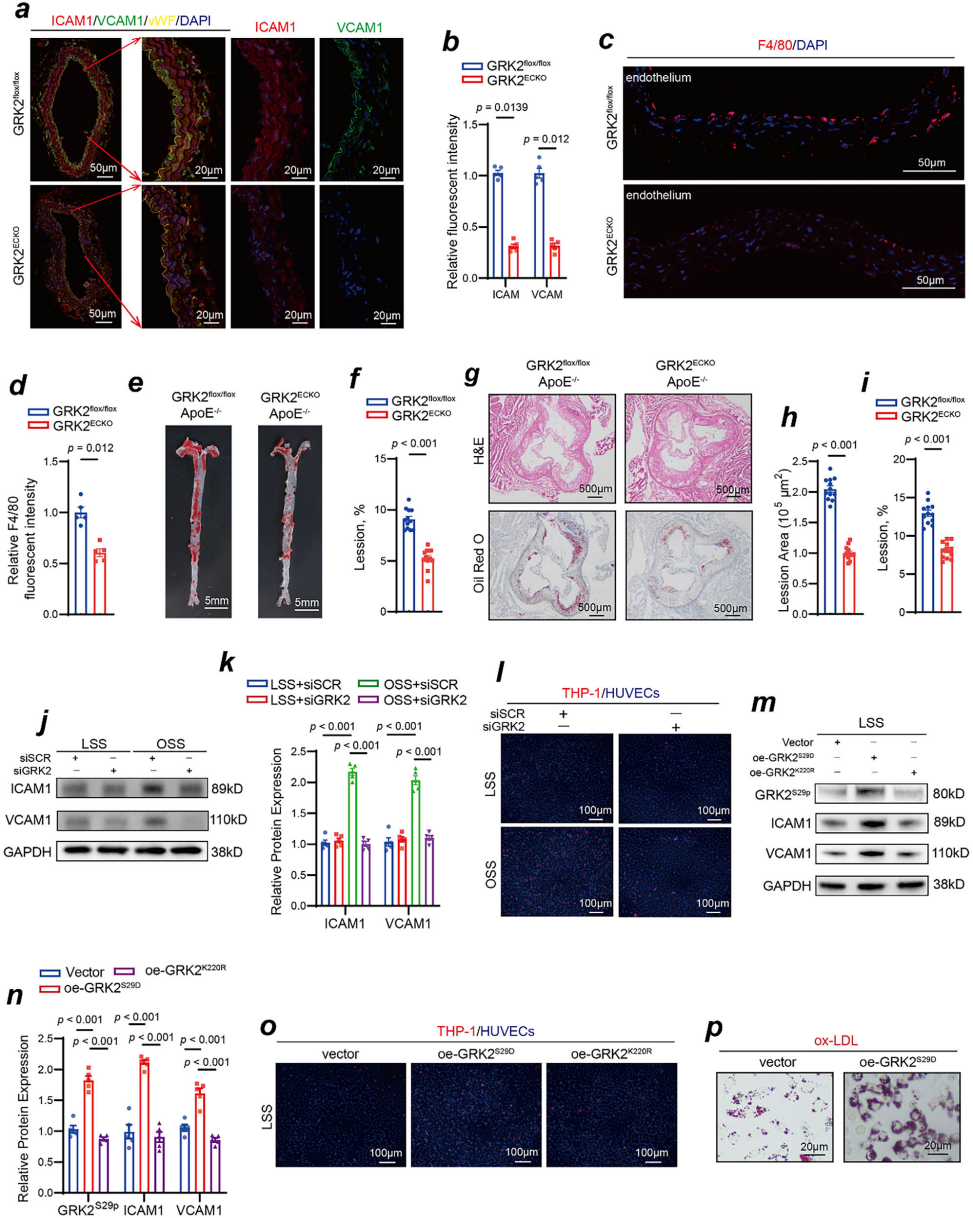
GRK2 knockout alleviates OSS‐induced vascular endothelial dysfunction and atherosclerosis. a,b) Immunofluorescence staining for ICAM1 (red), VCAM1 (green), vWF (yellow) and DAPI (blue) in partially ligated left common carotid arteries isolated from GRK2^flox/flox^ mice and GRK2^ECKO^ mice. (b) Quantification of the relative ICAM1 and VCAM1 fluorescent intensity (n = 5, Student's *t* test). c,d) Immunofluorescence staining for F4/80 (red) and DAPI (blue) indicating the infiltration of macrophages in partially ligated left common carotid arteries isolated from GRK2^flox/flox^ mice and GRK2^ECKO^ mice. (d) Quantification of the relative F4/80 fluorescent intensity (n = 5, Student's *t* test). e) Arterial tissues were isolated from GRK2^flox/flox^; ApoE^‐/‐^ mice and GRK2^ECKO^; ApoE^‐/‐^ mice fed a high‐fat Western diet, and their atherosclerotic lesions were examined via Oil Red O staining. f) Quantification of atherosclerotic lesions (n = 12, Student's *t* test). g) Aortic roots isolated from GRK2^flox/flox^; ApoE^‐/‐^ mice and GRK2^ECKO^; ApoE^‐/‐^ mice fed a high‐fat Western diet were sectioned for H&E staining and Oil Red O staining to examine atherosclerotic lesions. h,i) Quantification of atherosclerotic lesions (n = 12, Student's *t* test). j) ICAM1 and VCAM1 expression in GRK2 siRNA treated‐HUVECs and scramble siRNA treated‐HUVECs exposed to different shear stresses was detected via Western blotting. k) Quantification of ICAM1 and VCAM1 protein expression levels (n = 5, one‐way ANOVA). l) Adhesion of THP‐1 monocytes from GRK2 siRNA treated‐HUVECs and scramble siRNA treated‐HUVECs exposed to different shear stresses. m) GRK2^S29p^, ICAM1 and VCAM1 expression levels were detected by Western blotting in constitutively activated GRK2^S29D^‐overexpressing HUVECs, and dysfunctional GRK2^K220R^‐overexpressing HUVECs exposed to LSS. n) Quantification of GRK2^S29p^, ICAM1 and VCAM1 protein expression levels (n = 5; one‐way ANOVA). o) Compared with HUVECs overexpressing the wild‐type plasmid, HUVECs overexpressing the continuously activated GRK2^S29D^ plasmid presented increased monocyte adhesion under LSS conditions. p) RAW macrophages engulfed ox‐LDL after treatment with conditioned medium from constitutively activated GRK2^S29D^‐overexpressing HUVECs and GRK2^K220R^‐overexpressing HUVECs (n = 5, one‐way ANOVA).

In this study, we successfully established vascular endothelial cell‐specific GRK2 conditional knockout mice. Figure S5a, Supporting Information shows the generation scheme. Immunofluorescence staining confirmed the successful knockout of GRK2 in the vascular endothelium of GRK2^ECKO^ mice (Figure S5b, Supporting Information). Figure S5c, Supporting Information shows the results of genotyping via PCR. We successfully generated vascular endothelial cell‐specific GRK2 conditional knockout mice (GRK2^ECKO^: GRK2^flox/flox^ Cdh5‐CreERT2) and their control littermates (GRK2^flox/flox^). To further confirm the vascular endothelial cell‐specific knockout of GRK2, we performed Western blot analysis of aortic tissues with mechanically denuded endothelium. As shown in Figure S5d, Supporting Information, GRK2 expression was successfully knocked out in endothelium of GRK2^ECKO^ mice. However, GRK2 expression levels in the vascular medial layer were comparable between GRK2^ECKO^ and GRK2^flox/flox^ mice, indicating that vascular endothelial‐specific knockout of GRK2 did not affect GRK2 expression in vascular smooth muscle cells (Figure S5e,f, Supporting Information). To verify that other GRKs family members were not affected in GRK2^ECKO^ mice, we isolated the aorta endothelium of GRK2^flox/flox^ and GRK2^ECKO^ mice and performed Western blot analysis. GRK1 and GRK7 are predominantly expressed in retinal photoreceptor cells and exhibit negligible expression in most non‐retinal tissues, including vascular endothelial cells. Our results revealed that the expression levels of other GRKs family members (GRK3, GRK4, GRK5, and GRK6) remained unchanged between GRK2^ECKO^ and GRK2^flox/flox^ mice, confirming that the knockout of GRK2 did not induce compensatory alterations in other GRKs family members (Figure S5g,h, Supporting Information). We subsequently performed partial ligation of the carotid artery in both GRK2^ECKO^ mice and GRK2^flox/flox^ littermates to assess whether vascular endothelial knockout of GRK2 could alleviate OSS‐induced inflammation in the vascular endothelium. The immunofluorescence results shown in **Figure** **3**a indicate that after partial ligation of the carotid artery, the expression levels of ICAM1 and VCAM1 were significantly lower in GRK2^ECKO^ mice than in GRK2^flox/flox^ mice (Figure 3a,b). Compared with GRK2^flox/flox^ mice, F4/80 immunofluorescence revealed a significant reduction in macrophage infiltration in the vessels of GRK2^ECKO^ mice following partial ligation of the carotid artery (Figure 3c,d). By crossing GRK2^ECKO^ mice with ApoE^‐/‐^ mice, we successfully obtained GRK2^ECKO^ mice on an ApoE^‐/‐^ background (GRK2^ECKO^; ApoE^‐/‐^) and their control littermates (GRK2^flox/flox^; ApoE^‐/‐^). After 12 weeks of high‐fat Western diet consumption, Oil Red O staining revealed that the area and severity of plaques were significantly reduced in GRK2^ECKO^; ApoE^‐/‐^ mice (Figure 3e,f). Hematoxylin‐eosin (H&E) and Oil Red O staining of aortic root sections further confirmed that the severity of atherosclerosis was significantly lower in GRK2^ECKO^; ApoE^‐/‐^ mice than in GRK2^flox/flox^; ApoE^‐/‐^ mice (Figure 3g–i). We compared body weight and cholesterol levels between two groups of mice and found no significant differences between GRK2^ECKO^; ApoE^‐/‐^ and GRK2^flox/flox^; ApoE^‐/‐^ (Table S10, Supporting Information). To investigate whether GRK2 knockdown could alleviate OSS‐induced inflammation in the vascular endothelial cells in vitro, we designed three independent small interfering RNA (siRNA) sequences targeting GRK2. The Western blot results (Figure S6a,b, Supporting Information) confirmed that all three GRK2‐targeting siRNA sequences effectively reduced GRK2 protein expression in HUVECs, and siRNA #2 was selected for subsequent experiments. We found that GRK2 knockdown led to a reduction in ICAM1 and VCAM1 expression levels in HUVECs after exposure to OSS in vitro (Figure 3j,k). Since both ICAM1 and VCAM1 are monocyte adhesion molecules, we conducted THP‐1 monocyte recruitment experiments. The results revealed that monocyte adhesion was significantly lower in GRK2‐knockdown HUVECs than in control HUVECs after exposure to OSS, further confirming that GRK2 knockdown could abrogate OSS‐induced recruitment of inflammatory cells (Figure 3l). Compared with HUVECs overexpressing the wild‐type plasmid, HUVECs overexpressing the GRK2^S29D^ plasmid, which are constitutively active, presented significantly increased expression levels of ICAM1 and VCAM1 after 2 h of LSS stimulation (Figure 3m,n), along with increased monocyte adhesion (Figure 3o). Given that overexpression of the GRK2^S29D^ plasmid induces inflammation in the vascular endothelial cells, we aimed to investigate its effects under physiological rather than pathological conditions. Conversely, HUVECs overexpressing the GRK2^K220R^ plasmid, which disrupts the kinase activity of GRK2, showed no significant changes in ICAM1 and VCAM1 expression levels (Figure 3m,n) or in monocyte adhesion (Figure 3o). These findings suggest that the kinase activity of GRK2 may play a critical role in OSS‐induced vascular endothelial dysfunction. Furthermore, in addition to the increase in the expression of membrane‐bound adhesion molecules ICAM1 and VCAM1 by overexpression of GRK2^S29D^ in HUVECs, we observed that conditioned medium derived from HUVECs overexpressing GRK2^S29D^ promoted ox‐LDL uptake by macrophages, suggesting the presence of secreted pro‐inflammatory factors that enhance macrophage activation (Figure 3p).

### 2.4 AP‐1 Is a Downstream of GRK2 and Plays a Role in OSS‐Induced Vascular Endothelial Dysfunction

**Figure 4:**
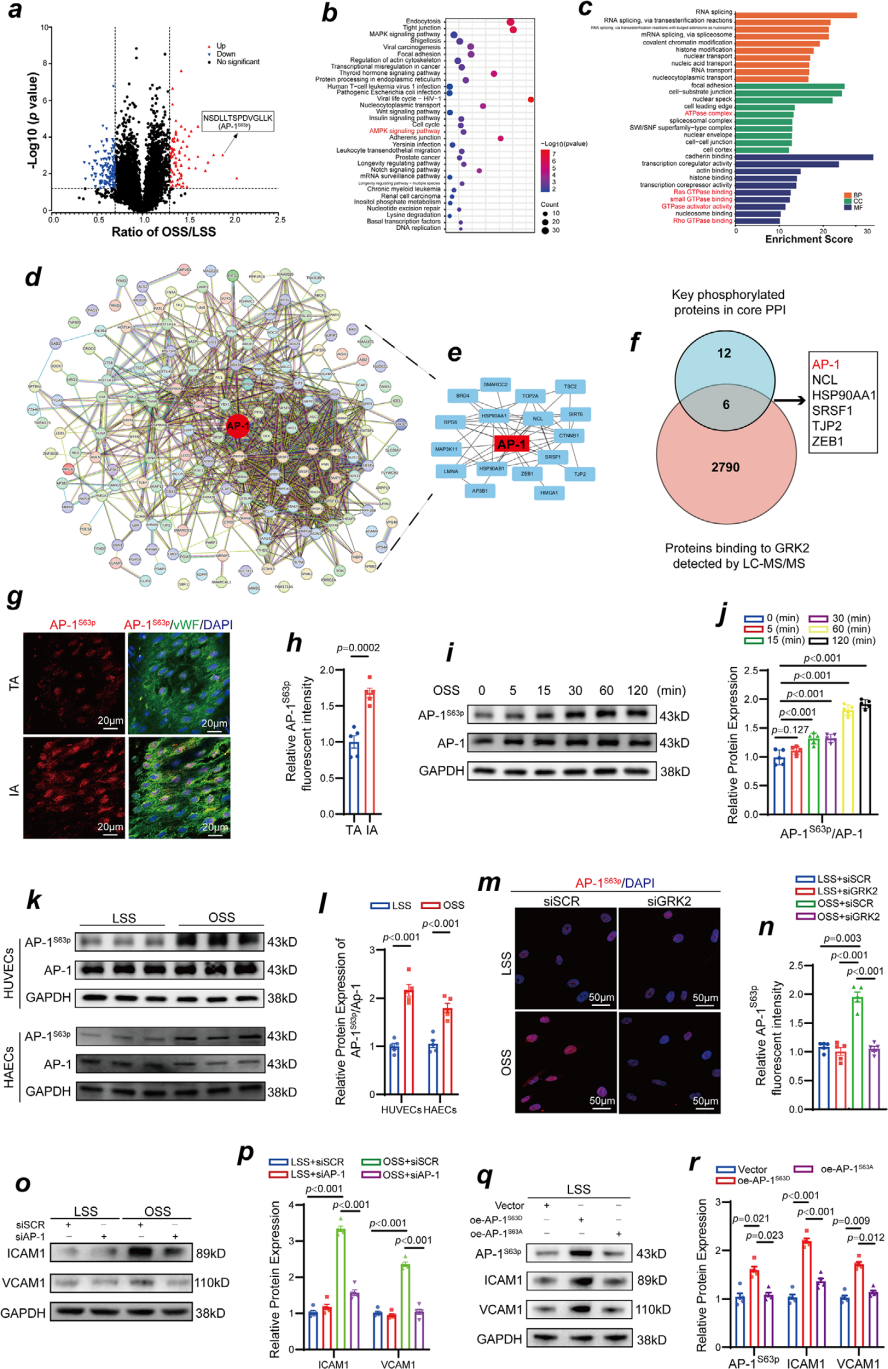
AP‐1^S63p^ is a downstream of GRK2 and plays a role in OSS‐induced vascular endothelial dysfunction. a) Volcano plot revealing differentially expressed phosphorylated proteins in vascular endothelial cells exposed to OSS. b) GO functional enrichment analysis of differentially expressed phosphorylated proteins. c) KEGG pathway enrichment analysis of differentially expressed phosphorylated proteins. d) PPI network based on differentially expressed phosphorylated proteins. e) Core PPI network. f) Venn diagram showing the shared proteins between the key phosphorylated proteins in vascular endothelial cells exposed to OSS and the LC‒MS/MS‐detected proteins that potentially bind to GRK2. g) *Enface* immunofluorescence staining of AP‐1^S63p^ (red), vWF (green), and DAPI (blue) in the endothelium of the inner curvature of the aorta arch and the thoracic aorta isolated from C57BL/B6J mice. h) Quantification of the AP‐1^S63p^ fluorescent intensity level (n = 5, one‐way ANOVA). i) HUVECs subjected to OSS for 0, 5, 15, 30, 60, and 120 min. The proteins in the cell lysate were evaluated via Western blotting with the indicated antibodies. j) Quantification of AP‐1^S63p^ protein expression level (n = 5, one‐way ANOVA). (k) HUVECs and HAECs were subjected to LSS and OSS for 120 min. The proteins in the cell lysate were analyzed via Western blotting with the indicated antibodies. l) Quantification of AP‐1^S63p^ protein expression level (n = 5, student's *t* test). m) Immunofluorescence staining for AP‐1^S63p^ (red) and DAPI (blue) in GRK2 siRNA treated‐HUVECs and scramble siRNA treated‐HUVECs exposure to different shear stresses. n) Quantification of AP‐1^S63p^ fluorescent intensity level (n = 5, one‐way ANOVA). o) ICAM1 and VCAM1 expression in AP‐1 siRNA treated‐HUVECs and scramble siRNA treated‐HUVECs exposed to different shear stress stresses was detected via Western blot. p) Quantification of ICAM1 and VCAM1 protein expression levels (n = 5, one‐way ANOVA). q) AP‐1^S63p^, ICAM1 and VCAM1 expression in constitutively activated AP‐1^S63D^‐overexpressing HUVECs and inactivated AP‐1^S63A^‐ overexpressing HUVECs was detected via Western blotting after exposure to LSS. r) Quantification of AP‐1^S63p^, ICAM1 and VCAM1 protein expression levels (n = 5, one‐way ANOVA).

Given that GRK2 is a serine/threonine kinase, we performed phosphoproteomic analysis to investigate OSS‐induced changes in the global protein phosphorylation profile. As shown in the volcano plot in **Figure** **4**a, the results indicated that after OSS stimulation, a total of 279 peptide segments in HUVECs exhibited changes in phosphorylation levels, with 86 peptides with increased phosphorylation and 193 peptides with decreased phosphorylation. Notably, the phosphorylation of peptide segment with the sequence NSDLLTSPDVGLLK was significantly increased, and the sequence corresponded to AP‐1 phosphorylated at serine 63 (AP‐1^S63p^). We subsequently performed functional enrichment analysis on the differentially phosphorylated proteins identified in the phosphoproteomic analysis. Figure 4b displays the results of the KEGG pathway enrichment analysis, revealing significant enrichment of inflammation‐related signaling pathways, including the MAPK signaling pathway, as well as energy metabolism‐related pathways. Furthermore, as illustrated in Figure 4c, the results of the GO functional enrichment analysis indicated that the differentially phosphorylated proteins were enriched in terms related to inflammation and energy metabolism. In addition, we constructed a protein‐protein interaction (PPI) network based on all the differentially phosphorylated proteins, as shown in Figure 4d. Figure 4e presents the core PPI network, where AP‐1 occupies a central position, indicating closer interactions with other proteins within the PPI network. The Venn diagram in Figure 4f illustrates the intersection of key differentially phosphorylated proteins from the core PPI network and proteins potentially binding with GRK2 identified through liquid chromatography‒tandem mass spectrometry (LC‐MS/MS). AP‐1 is one of six proteins that interact with GRK2 and plays a role in OSS‐induced vascular endothelial dysfunction (Figure S7, Supporting Information). In vivo, the results of the *enface* immunofluorescence imaging revealed that AP‐1^S63p^ expression was elevated in endothelial cells in the inner curvature of the aortic arch, exposing to OSS (Figure 4g,h). In vitro experiments involving HUVECs subjected to OSS over different periods of time (0, 5, 15, 30, 60, and 120 min) demonstrated that AP‐1^S63p^ levels increased in a time‐dependent manner, whereas the expression level of AP‐1 remained unchanged (Figure 4i,j). Compared with that after exposure to LSS, the phosphorylation level of AP‐1^S63p^ was elevated in both HUVECs and HAECs after exposure to OSS (Figure 4k,l). To further investigate whether AP‐1 plays a role in inflammation in the vascular endothelial cells induced by OSS, we constructed three independent siRNA sequences targeting AP‐1, which resulted in significant reductions in AP‐1^S63p^ and AP‐1 expression levels in HUVECs. For subsequent experiments, we selected the first siRNA sequence (#1) (Figure S8, Supporting Information). We conducted immunofluorescence staining following GRK2 knockdown using siRNA. The results indicated that AP‐1^S63p^ localized in the nucleus presented increased phosphorylation levels in HUVECs after 2 h of OSS stimulation compared with those after LSS, but this phosphorylation level decreased upon GRK2 knockdown (Figure 4m,n). Figure 4o,p also indicated that AP‐1 knockdown led to reduced expression levels of ICAM1 and VCAM1 in HUVECs exposed to OSS, suggesting that AP‐1 knockdown could alleviate OSS‐induced inflammation in the vascular endothelial cells (Figure 4o,p). Compared with HUVECs overexpressing the wild‐type AP‐1 plasmid, those overexpressing the constitutively phosphorylated AP‐1^S63D^ mutant presented significantly increased ICAM1 and VCAM1 expression under LSS (Figure 4q,r), whereas HUVECs overexpressing the continuously inactive AP‐1^S63A^ plasmid did not show an increase in ICAM1 and VCAM1 expression levels (Figure 4q,r). The results of the monocyte adhesion experiment further confirmed that, compared with HUVECs overexpressing the wild‐type plasmid, HUVECs overexpressing the continuously activated AP‐1^S63D^ plasmid presented increased monocyte adhesion after LSS stimulation, whereas HUVECs overexpressing the inactive AP‐1^S63A^ plasmid did not exhibit increased monocyte adhesion (Figure S9a, Supporting Information).

### 2.5 GRK2 Mediates OSS‐Induced Inflammation in the Vascular Endothelium via AP‐1 Phosphorylation

**Figure 5:**
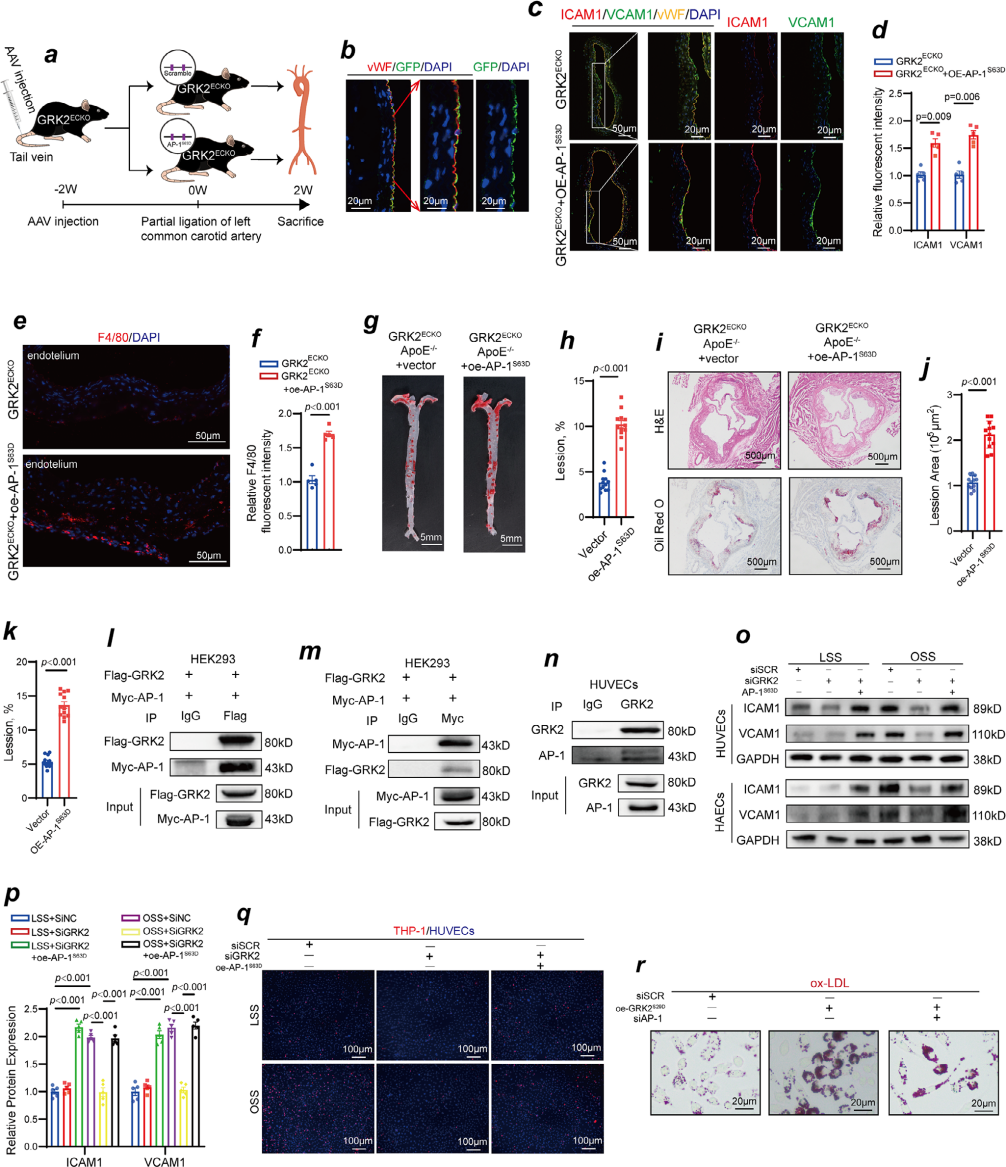
AAV9‐mediated overexpression of AP‐1^S63D^ under the control of the ICAM‐2 promoter in vascular endothelial cells promotes endothelial dysfunction and accelerates atherosclerosis. a) Schematic figure of the administration of ICAM2 promoter‐driven AAV9 carrying AP‐1^S63D^ or vector via tail vein injection in GRK2^ECKO^ mice. b) Co‐immunofluorescence staining of viral GFP (green) and vWF (red). c) Immunofluorescence staining for ICAM1 (red), VCAM1 (green), vWF (yellow) and DAPI (blue) in partially ligated left common carotid arteries isolated from GRK2^ECKO^ mice following the administration of ICAM‐2 promoter‐driven AAV9 carrying AP‐1^S63D^ or vector. d) Quantification of ICAM1 and VCAM1 protein expression levels (n = 5, Student's *t* test). e) Immunofluorescence staining for F4/80 (red) and DAPI (blue) indicating the infiltration of macrophages in partially ligated left common carotid arteries isolated from GRK2^ECKO^ mice following the administration of ICAM‐2 promoter‐driven AAV9 carrying AP‐1^S63D^ or vector. f) Quantification of the F4/80 fluorescent intensity (n = 5, Student's *t* test). g) Oil Red O staining indicating increased atherosclerotic lesions in endothelial cell specific AP‐1^S63D^‐overexpressing GRK2^ECKO^; ApoE^‐/‐^ mice fed a high‐fat Western diet. h) Quantification of atherosclerotic lesions (n = 12, Student's *t* test). i) H&E staining and Oil Red O staining of the aortic roots of GRK2^ECKO^; ApoE^‐/‐^ hyperlipidemic mice following the administration of ICAM‐2 promoter‐driven AAV9 carrying AP‐1^S63D^ or vector. j,k) Quantification of atherosclerotic lesions (n = 12, Student's *t* test). l,m) Co‐IP experiment revealing the direct binding of AP‐1 to GRK2 in HEK293 cells (n = 3). n) Co‐IP experiments revealing the direct binding of AP‐1 to GRK2 in HUVECs (n = 3). o) Rescue Western blotting experiments demonstrating that AP‐1^S63D^ overexpression abrogated the protective effects of GRK2 knockdown on vascular endothelial cells exposed to OSS in HUVECs and HAECs. p) Quantification of ICAM1 and VCAM1 protein expression levels in HUVECs (n = 5, one‐way ANOVA). q) Rescue THP‐1 monocyte adhesion assay results indicating that overexpressing AP‐1^S63D^ abrogated the protective effects of GRK2 knockdown on vascular endothelial cells exposed to OSS. r) RAW macrophages engulf ox‐LDL after treatment with conditioned medium from GRK2^S29D^‐overexpressing HUVECs following AP‐1 siRNA or scramble siRNA treatment.

To further verify that AP‐1^S63p^ is a direct downstream target of GRK2 in mediating inflammation in the vascular endothelium caused by OSS, we utilized adeno‐associated virus 9 (AAV9) with an ICAM2 promoter to specifically overexpress AP‐1^S63D^ in the vascular endothelial cells of GRK2^ECKO^ mice, as illustrated in **Figure** **5**a. Co‐immunofluorescence staining for vWF and green fluorescent protein (GFP) demonstrates that AAV9‐GFP was successfully delivered to vascular endothelial cells (Figure 5b). Following partial carotid artery ligation, the specific overexpression of AP‐1^S63D^ in the endothelium of GRK2^ECKO^ mice led to increased expression levels of ICAM1 and VCAM1 (Figure 5c,d). Immunofluorescence staining for F4/80 indicated increased macrophage infiltration in the vessels of mice overexpressing AP‐1^S63D^ (Figure 5e,f). GRK2^ECKO^; ApoE^‐/‐^ mice were fed a high‐fat Western diet for 12 weeks after the injection of AAV9 oe‐AP‐1^S63D^ or vector. Oil Red O staining revealed that the plaques area and severity in GRK2^ECKO^; ApoE^‐/‐^ mice treated with AAV9 oe‐AP‐1^S63D^ were significantly aggravated (Figure 5g,h). Histological analysis with H&E and Oil Red O staining of the aortic root further confirmed that, compared with their vector‐transfected counterparts, GRK2^ECKO^; ApoE^‐/‐^ mice overexpressing AP‐1^S63D^ presented significantly exacerbated atherosclerotic lesions (Figure 5i,k). Table S11, Supporting Information compares the body weight and cholesterol levels between two groups of GRK2^ECKO^; ApoE^‐/‐^ mice fed a high‐fat Western diet and administered either AAV oe‐AP‐1^S63D^ or an AAV vector, and there is no significant difference between the two groups. In addition, we used AAV9 driven by the endothelial‐specific ICAM2 promoter to overexpress GRK2^S29D^ in vascular endothelial cells. Vascular endothelial cell‐specific overexpression of GRK2^S29D^ led to marked upregulation of ICAM1 and VCAM1 expression in the vascular endothelium (Figure S10a,b, Supporting Information). Immunofluorescence staining of F4/80 further revealed increased macrophage infiltration in the vessels of mice overexpressing GRK2^S29D^ (Figure S10c,d, Supporting Information). Moreover, ApoE^‐/‐^ mice treated with AAV9‐GRK2^S29D^ or vector were fed a high‐fat Western diet for 12 weeks. Oil Red O staining revealed significantly increased plaques in GRK2^S29D^‐overexpressing mice (Figure S10e,f, Supporting Information). Consistently, both H&E and Oil Red O staining of the aortic root revealed that vascular endothelial cell‐specific overexpression of GRK2^S29D^ significantly increased atherosclerotic lesion formation and severity (Figure S10g–i, Supporting Information). There is no significant difference between the ApoE^‐/‐^ mice fed a high‐fat Western diet and administered either AAV oe‐GRK2^S29D^ or an AAV vector (Table S12, Supporting Information). Using Co‐immunoprecipitation (Co‐IP), we validated the interaction between GRK2 and AP‐1 in human embryonic kidney 293 (HEK293) cells and HUVECs. In HEK293 cells, coexpressing Flag‐tagged GRK2 and Myc‐tagged AP‐1 enabled exogenous Co‐IP. Flag‐based immunoprecipitation pulled down AP‐1 (Figure 5l), and reciprocal Myc‐based Co‐IP confirmed the association of GRK2 with AP‐1 (Figure 5m). Endogenous Co‐IP in HUVECs with anti‐GRK2 antibodies further confirmed the association between GRK2 and AP‐1 (Figure 5n). These experimental findings provide strong evidence that GRK2 can interact with AP‐1. To further confirm that AP‐1^S63p^ is a direct downstream target of GRK2 in the context of OSS‐induced inflammation in the vascular endothelium, we performed rescue experiments in both HUVECs and HAECs in vitro. We knocked down GRK2 in vascular endothelial cells using siRNA and overexpressed AP‐1^S63D^ via plasmid transfection. In vascular endothelial cells subjected to OSS, knockdown of GRK2 reduced the increase in ICAM1 and VCAM1 expression levels induced by OSS; however, further overexpression of AP‐1^S63D^ in combination with GRK2 knockdown abrogated the protective effect of GRK2 knockdown against OSS‐induced inflammation in the vascular endothelial cells (Figure 5o,p; Figure S9b, Supporting Information). The results of the monocyte adhesion experiments confirmed that in OSS‐stimulated vascular endothelial cells, further overexpression of AP‐1^S63D^ abrogated the protective effect of GRK2 knockdown, leading to a significant increased monocyte adhesion compared with that in cells with GRK2 knockdown alone (Figure 5q). Previous studies showed that AP‐1 plays a crucial role in mediating inflammatory responses by enhancing the production of cytokines such as CCL2, TNF‐α, and IL‐6, which are known to promote macrophage activation and lipid uptake. In the present study, RAW macrophages exhibited increased ox‐LDL uptake after treatment with conditioned medium from GRK2^S29D^‐overexpressing HUVECs, and this uptake was significantly lower when AP‐1 was knocked down in HUVECs than in cells treated with scrambled siRNA, suggesting that GRK2^S29D^ promotes the secretion of pro‐inflammatory mediators in an AP‐1–dependent manner (Figure 5r).

### 2.6 Transcriptional Regulation of Nuclear Receptor Subfamily 4 Group A 1 (NR4A1) by AP‐1 in OSS‐Induced Vascular Endothelial Dysfunction

**Figure 6:**
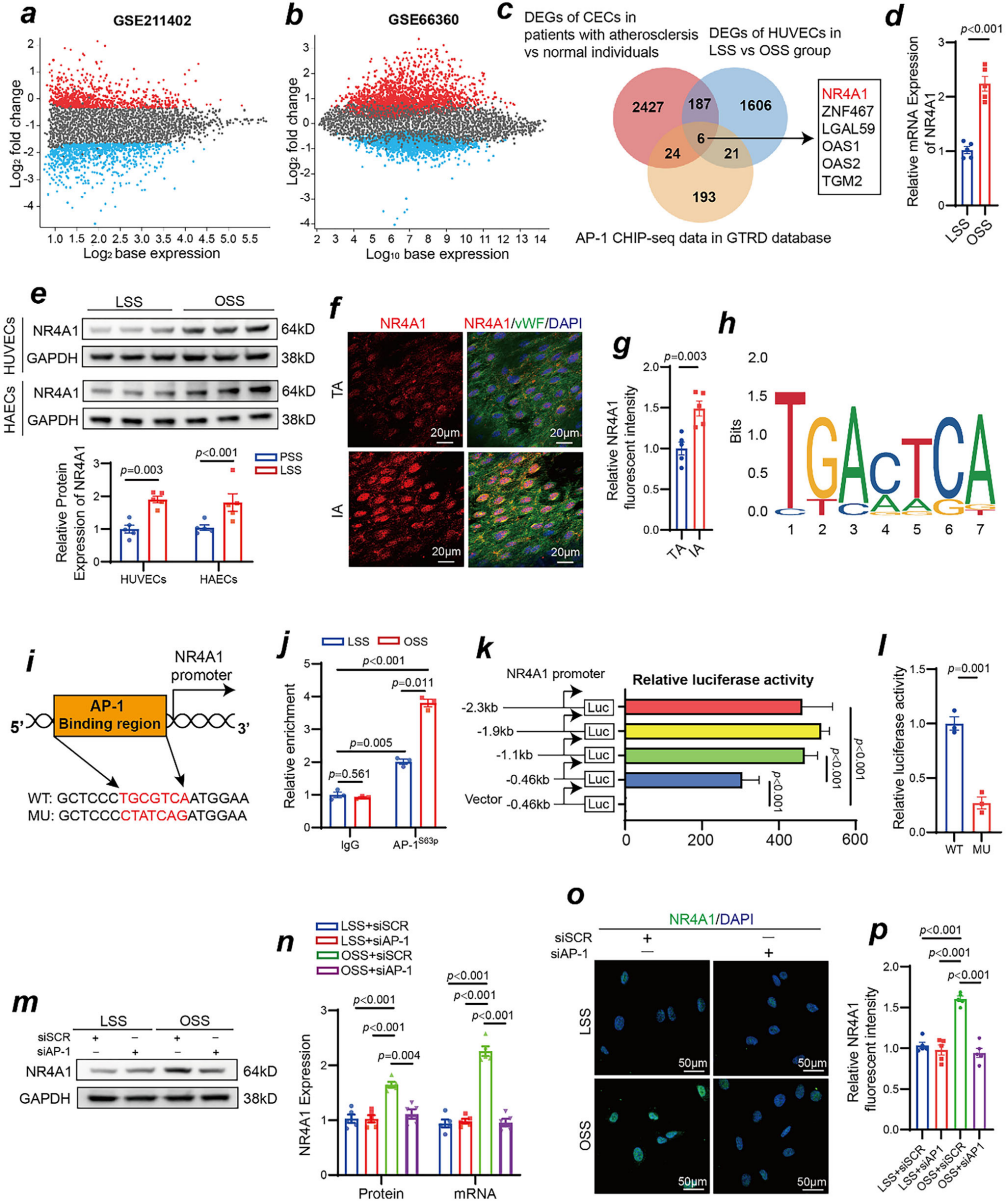
NR4A1 is transcriptionally regulated by AP‐1. a) Volcano plot showing the differentially expressed genes in the GSE211402 dataset. b) Volcano plot showing the differentially expressed genes in the GSE66360 dataset. c) Venn diagram showing the downstream factors that may be transcriptionally regulated by AP‐1 and play important roles in vascular endothelial dysfunction and atherosclerosis induced by OSS. d) qPCR experiment validating the effect of OSS on NR4A1 mRNA expression in HUVECs (n = 5, Student's *t* test). e) Validation of the effect of OSS on NR4A1 protein expression in HUVECs and HAECs via Western blotting (n = 5, Student's *t* test). f) *Enface* immunofluorescence staining of NR4A1 (red), vWF (green), and DAPI (blue) in the inner curvature of the aortic arch and thoracic aorta isolated from C57BL/6J mice. g) Quantification of the NR4A1 fluorescent intensity (n = 5, one‐way ANOVA). h) Potential binding sequences of the NR4A1 promoter region and AP‐1 predicted by the JASPAR database. i) Schematic diagram of the putative AP‐1 binding site in the NR4A1 promoter and the mutant. j) ChIP‒qPCR experiment validating the transcriptional regulation of NR4A1 by AP‐1 (n = 3, one‐way ANOVA). HUVECs were exposed to OSS and LSS, and then crosslinked chromatin was extracted and immunoprecipitated with anti‐AP‐1^S63p^ or IgG antibodies. The immunoprecipitated DNA was amplified by PCR. k) HUVECs were co‐transfected with the AP‐1 expression vector (AP‐1 WT) and different NR4A1 truncation promoter‐reporter plasmids to identify the promoter activity regions where AP‐1 regulates NR4A1 transcription (n = 5, one‐way ANOVA). l) HUVECs were cotransfected with the AP‐1 expression vector (AP‐1 WT), the NR4A1 promoter‐reporter plasmid (NR4A1 WT) or the NR4A1 promoter‐reporter binding site mutant (NR4A1 mutant). 48 h after transfection, cell lysates were prepared for the luciferase assay. The data represent the relative NR4A1 promoter activity normalized to Renilla luciferase activity (n = 3, Student's *t* test). m) NR4A1 expression in AP‐1 siRNA treated‐HUVECs and scramble siRNA treated‐HUVECs exposed to different shear stresses was evaluated via Western blotting and PCR. n) Quantification of NR4A1 protein and mRNA expression levels (n = 5, one‐way ANOVA). o) Immunofluorescence staining for NR4A1 (red) and DAPI (blue) in AP‐1 siRNA treated‐HUVECs and scramble siRNA treated‐HUVECs exposed to different shear stresses. p) Quantification of the relative NR4A1 fluorescent intensity (n = 5, one‐way ANOVA).

Since AP‐1 is a transcription factor, we analyzed transcriptomic data from public databases related to vascular endothelial dysfunction and atherosclerosis. The GSE211402 dataset includes transcriptomic sequencing data from vascular endothelial cells of atherosclerosis patients and nonatherosclerotic individuals. Through our analysis, we identified a total of 2427 differentially expressed genes (**Figure** **6**a). The GSE66360 dataset includes transcriptomic data from HUVECs subjected to OSS and those subjected to LSS, resulting in the identification of 1606 differentially expressed genes (Figure 6b). Additionally, we obtained 193 potential target genes most likely regulated by AP‐1 from chromatin immunoprecipitation sequencing (ChIP‐seq) datasets available in the gene transcription regulation database (GTRD). Using a Venn diagram to identify the shared genes, we identified six candidate downstream molecules potentially regulated by AP‐1 and involved in vascular endothelial dysfunction and atherosclerosis induced by OSS: NR4A1, ZNF467, LGAL59, OAS1, OAS2, and TGM2 (Figure 6c). Since NR4A1 had the highest score in the ChIP‐seq analysis, we selected it for further study. The qPCR results shown in Figure 6d indicate that NR4A1 mRNA expression levels increased significantly in response to OSS compared with LSS in HUVECs. Furthermore, the Western blot results shown in Figure 6e confirmed a significant increase in NR4A1 protein expression under OSS in both HUVECs and HAECs. Compared with that in the LSS region, NR4A1 expression in the OSS region was elevated according to *enface* immunofluorescence staining in vivo (Figure 6f,g). As shown in Figure 6h, we predicted potential binding sequences of AP‐1 within the NR4A1 promoter region using the JASPAR database. By comparing sequences, we identified the TGCGTCA segment within the NR4A1 promoter region as a possible binding site for AP‐1 (Figure 6i). The ChIP‒qPCR results revealed that the recruitment of the NR4A1 promoter region to AP‐1^S63p^ was significantly greater than that of the immunoglobulin G (IgG) controls. Additionally, compared with LSS, OSS stimulation increased the binding of AP‐1^S63p^ to the NR4A1 promoter region (Figure 6j). In dual‐luciferase reporter assays, we detected no significant differences in luciferase activity among the −2.3, −1.9, and −1.1 kb truncated plasmids; however, the luciferase activity of the −0.46 kb truncated plasmid was significantly lower than that of the −1.1 kb truncated plasmid, suggesting that the −1.1 to −0.46 kb region may be an active regulatory region for AP‐1 transcriptional control of NR4A1 (Figure 6k). By mutating the binding sites of the AP‐1 and NR4A1 promoters, we observed that luciferase activity was significantly lower in the mutant group than in the wild‐type group (Figure 6l). Subsequently, we also found that AP‐1 knockdown significantly reduced the increase in NR4A1 expression induced by OSS (Figure 6m,n). The immunofluorescence results also revealed that NR4A1 localized to the nucleus and that AP‐1 knockdown significantly decreased the increase in NR4A1 expression induced by OSS (Figure 6o,p). Moreover, the Western blot (Figure S11a,b, Supporting Information) and immunofluorescence (Figure S11c,d, Supporting Information) results indicated that GRK2 knockdown also decreased the OSS‐induced increase in NR4A1 expression.

### 2.7 NR4A1 Anchors Liver Kinase B1 in the Nucleus to Disrupt Energy Metabolism in Vascular Endothelial Cells

**Figure 7:**
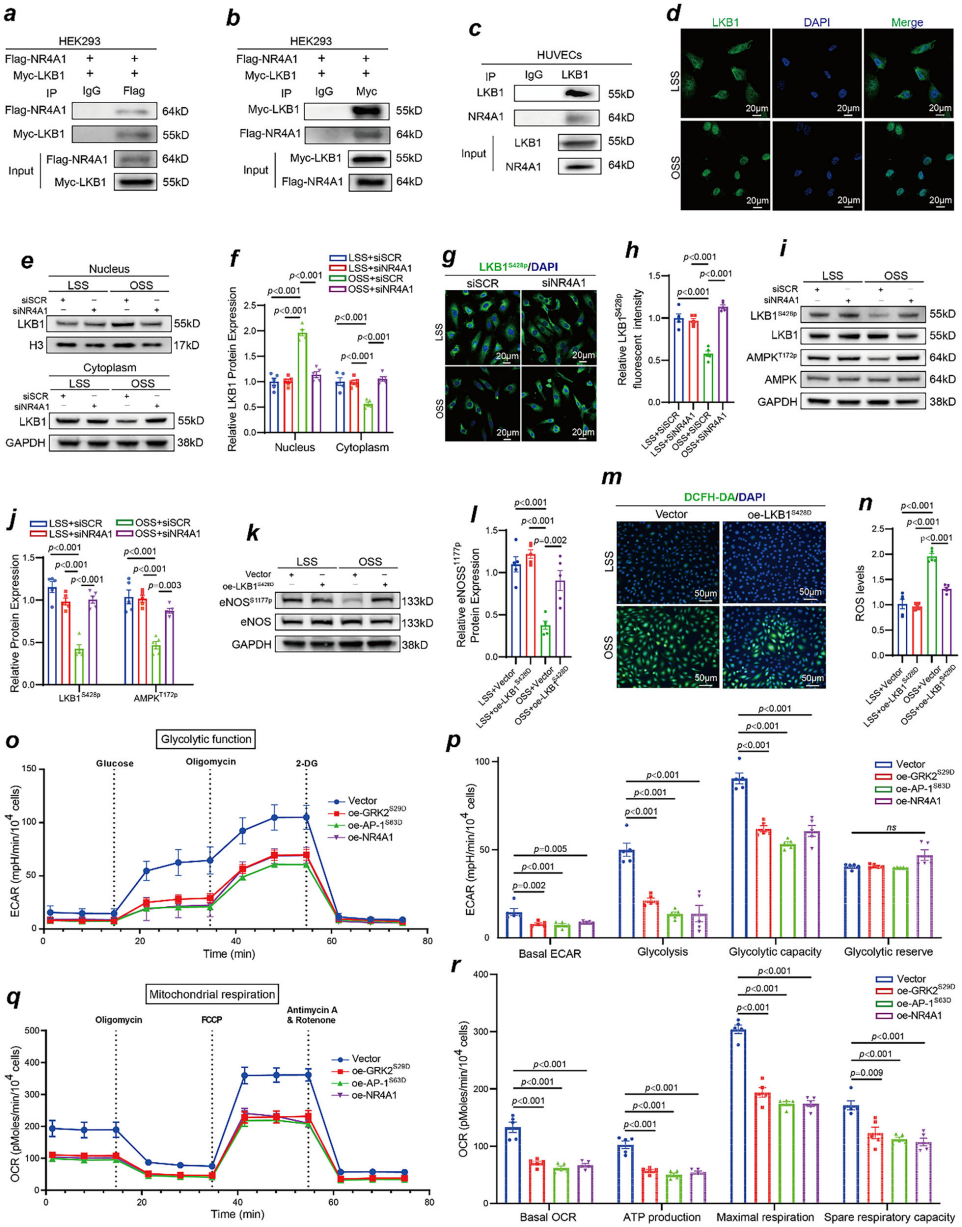
NR4A1 functions in vascular endothelial cell energy metabolism disorders induced by OSS by binding to LKB1 in the nucleus. a,b) Co‐IP experiment revealing the direct binding of NR4A1 to LKB1 in HEK293 cells (n = 3). c) Co‐IP experiment revealing the direct binding of NR4A1 to LKB1 in HUVECs (n = 3). d) Immunofluorescence staining for LKB1 (green) and DAPI (blue) in HUVECs exposed to different shear stresses. e) NR4A1 siRNA treated‐HUVECs and scramble siRNA treated‐HUVECs were exposed to OSS and LSS, and the LKB1 protein expression levels in the nucleus and cytoplasm of the HUVECs were detected by Western blotting. f) Quantification of LKB1 protein expression levels in the nucleus and cytoplasm (n = 5, one‐way ANOVA). g) Immunofluorescence staining for LKB1^S428p^ (green) and DAPI (blue) in NR4A1 siRNA treated‐HUVECs and scramble siRNA treated‐HUVECs exposed to different shear stresses. h) Quantification of LKB1 immunofluorescent intensity (n = 5, one‐way ANOVA). i) NR4A1 siRNA treated‐HUVECs and scramble siRNA treated‐HUVECs were subjected to different shear stresses, and the protein expression levels of LKB1^S428p^, LKB1, AMPK^T172p^, and AMPK were detected by Western blotting. j) Quantification of LKB1^S428p^ and AMPK^T172p^ protein expression levels (n = 5, one‐way ANOVA). k) eNOS^S1177p^ expression in constitutively activated LKB1^S428D^‐overexpressing HUVECs and vector control‐transfected HUVECs exposed to different shear stresses was detected via Western blotting. l) Quantification of eNOS^S1177p^ protein expression levels (n = 5, one‐way ANOVA). m) ROS levels were detected by DCFH‐DA (green) in constitutively activated LKB1^S428D^‐overexpressing HUVECs and vector control HUVECs exposed to different shear stresses. n) Quantification of ROS levels (n = 5, one‐way ANOVA). o) Extracellular acidification rate (ECAR) profiles showing glycolytic function in vector‐, GRK2^S29D^‐, AP‐1^S63D^‐, and NR4A1‐overexpressing HUVECs. The vertical lines indicate the time of addition of glucose (10 mmol L^−1^), oligomycin (3 µmol L^−1^), and 2‐deoxy‐D‐glucose (2‐DG) (100 mmol L^−1^). p) Quantification of glycolytic function parameters from (o); values are normalized to those of 10⁴ cells (n = 5, one‐way ANOVA). q) Oxygen consumption rate (OCR) profiles showing mitochondrial respiration function in vector‐, GRK2^S29D^‐, AP‐1^S63D^‐, and NR4A1‐overexpressing HUVECs. The vertical lines indicate the time of addition of oligomycin (3 µmol L^−1^), trifluoromethoxy phenylhydrazone (FCCP) (1 µmol L^−1^), antimycin A (1.5 µmol L^−1^), or rotenone (3 µmol L^−1^). r) Quantification of mitochondrial respiration function parameters from (q); values normalized to those of 10⁴ cells (n = 5, one‐way ANOVA).

Previous studies have shown that NR4A1 can influence cellular energy metabolism by binding to and anchoring LKB1 in the nucleus. In this study, we verified the interaction between NR4A1 and LKB1 via Co‐IP experiments in both HEK293 cells and HUVECs. In HEK293 cells, we co‐expressed Flag‐tagged NR4A1 and Myc‐tagged LKB1 for exogenous Co‐IP experiments. The results from immunoprecipitation with Flag antibodies indicated that LKB1 is among the proteins that bind to NR4A1 (**Figure** **7**a). Similarly, the results from immunoprecipitation with Myc antibodies revealed that NR4A1 is among the proteins that interact with LKB1 (Figure 7b). Additionally, endogenous Co‐IP experiments in HUVECs demonstrated that LKB1 is included among the proteins that bind to NR4A1 (Figure 7c). These findings confirm that NR4A1 interacts with LKB1. Immunofluorescence staining revealed that OSS increased the nuclear localization of LKB1 (Figure 7d). Next, we performed cytoplasmic‒nuclear separation experiments, and OSS stimulation led to a reduction in the cytoplasmic distribution of LKB1, which significantly increased upon NR4A1 knockdown (Figure 7e,f). These experiments confirmed that OSS induced an increase in NR4A1 expression, leading to the binding and anchoring of LKB1 in the nucleus. LKB1 phosphorylation is regulated by its nuclear import and export. When LKB1 resides in the nucleus, it cannot undergo phosphorylation. Immunofluorescence results further revealed that under OSS conditions, the nuclear distribution of LKB1 increased, whereas its phosphorylation level decreased. Following NR4A1 knockdown with siRNA, LKB1 was transported to the cytoplasm, and its level of phosphorylation increased (Figure 7g,h). The impact of OSS on LKB1 phosphorylation was also confirmed in HAECs. We found that after 2 h of OSS stimulation, the phosphorylation level of LKB1 significantly decreased (Figure S12a,b, Supporting Information). As LKB1 is a well‐established upstream regulator of the AMP‐activated protein kinase (AMPK) signaling pathway, an essential pathway for energy metabolism, we conducted Western blotting experiments to assess the phosphorylation levels of LKB1 and its downstream target AMPK. The Western blot results indicated that OSS significantly reduced the phosphorylation levels of LKB1 at S428 and of AMPK at T172. However, after NR4A1 knockdown with siRNA, the phosphorylation levels of LKB1^S428p^ and AMPK^T172p^ were partially restored (Figure 7i,j). Moreover, extensive research has demonstrated that disruptions in vascular endothelial cell energy metabolism can lead to decreased nitric oxide (NO) production and increased oxidative stress. We validated the levels of endothelial nitric oxide synthase (eNOS)^S1177p^ phosphorylation using Western blotting and assessed oxidative stress levels in vascular endothelial cells using reactive oxygen species (ROS) probes (DCFH‐DA probes). Our results indicated that OSS caused a decrease in eNOS^S1177p^ phosphorylation levels (Figure 7k,l) and increased oxidative stress levels (Figure 7m,n). Furthermore, overexpression of LKB1^S428D^ significantly abrogated the reduction in eNOS^S1177p^ phosphorylation (Figure 7k,l) and oxidative stress (Figure 7m,n) caused by OSS. Additionally, Western blot results revealed that knockdown of GRK2 restored the phosphorylation levels of LKB1^S428p^, AMPK^T172p^ and eNOS^S1177p^ (Figure S13a,b, Supporting Information). Moreover, we explored the effects of GRK2 deficiency on energy metabolism in HAECs subjected to OSS, and the results also revealed that knockdown of GRK2 abrogated the decreases in the phosphorylation levels of AMPK^T172p^ and eNOS^S1177p^ induced by OSS (Figure S13c,d, Supporting Information). Moreover, knockdown of GRK2 decreased the oxidative stress levels induced by OSS in HUVECs (Figure S14a,b, Supporting Information). Similar effects of alleviating the OSS‐induced decreases in the phosphorylation levels of LKB1^S428p^, AMPK^T172p^ and eNOS^S1177p^ were also found in AP‐1 siRNA‐treated HUVECs (Figure S15a,b, Supporting Information). To further investigate the regulatory effects of GRK2^S29p^ on downstream targets, we generated GRK2^WT^, GRK2^S29D^, and GRK2^S29A^ plasmids in HUVECs. After LSS stimulation, we found that overexpression of GRK2^S29D^ markedly increased the phosphorylation of AP‐1^S63p^, as well as the expression levels of the downstream target NR4A1. Moreover, overexpression of GRK2^S29D^ markedly decreased the phosphorylation of LKB1 and AMPK. In contrast, overexpression of GRK2^WT^ or GRK2^S29A^ did not induce these downstream changes (Figure S16a,b, Supporting Information). These results further underscore the importance of GRK2^S29p^ in modulating AP‐1 activity and its downstream signaling pathways. Next, using Seahorse flux analysis, we determined the effects of overexpression of GRK2^S29D^, AP‐1^S63D^, or NR4A1 on glycolytic and mitochondrial activities in vascular endothelial cells. We did not test the effects of LKB1 and AMPK, considering that their effects on energy metabolism are very clear. Compared with vector‐transfected control cells, GRK2^S29D^‐, AP‐1^S63D^‐, and NR4A1‐overexpressing HUVECs presented a significantly lower extracellular acidification rate (ECAR), an indicator of glycolytic activity and lactate production (Figure 7o). In addition to a reduced basal ECAR, both glucose‐induced glycolysis and maximal glycolytic capacity were markedly diminished in cells overexpressing GRK2^S29D^, AP‐1^S63D^, and NR4A1 (Figure 7p). Assessment of the oxygen consumption rate (OCR) further revealed that GRK2^S29D^, AP‐1^S63D^, and NR4A1 overexpression also attenuated mitochondrial respiration. Specifically, basal respiration, ATP production, maximal respiratory capacity and spare respiratory capacity were all lower in transduced cells than in control cells (Figure 7q,r).

### 2.8 Paroxetine Alleviates OSS‐Induced Vascular Endothelial Dysfunction and Atherosclerosis

**Figure 8:**
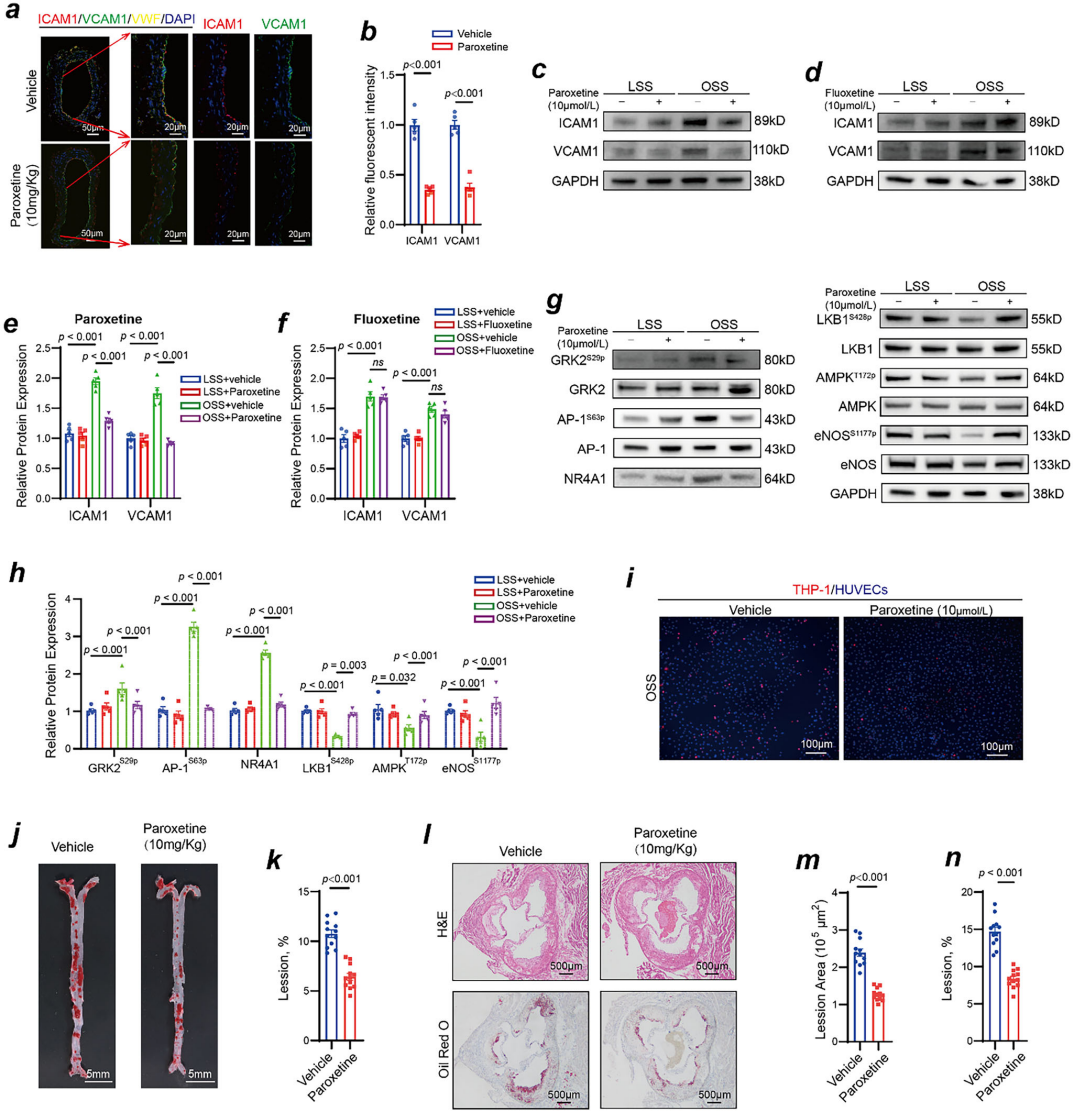
A GRK2 inhibitor (paroxetine) alleviates OSS‐induced vascular endothelial dysfunction and atherosclerosis in vivo and in vitro. a) Immunofluorescence staining for ICAM1 (red), VCAM1 (green), vWF (yellow) and DAPI (blue) in partially ligated left common carotid arteries isolated from vehicle group mice and paroxetine‐treated mice (10 mg kg^−1^ daily; I.g). b) Quantification of the relative ICAM1 and VCAM1 fluorescent intensities (n = 5, Student's *t* test). c) ICAM1 and VCAM1 were detected by Western blotting in paroxetine treated‐HUVECs and vehicle treated‐HUVECs exposed to different shear stresses. d) ICAM1 and VCAM1 were detected by Western blotting in fluoxetine treated‐HUVECs and vehicle treated‐HUVECs exposed to different shear stresses. e,f) Quantification of ICAM1 and VCAM1 protein expression levels (n = 5, one‐way ANOVA). g) The GRK2/AP‐1 signaling axis and its downstream targets were detected by Western blotting in paroxetine treated‐HUVECs and vehicle treated‐HUVECs exposed to different shear stresses. h) Quantification of the GRK2/AP‐1 signaling axis and its downstream targets expression levels (n = 5, one‐way ANOVA). i) Adhesion of THP‐1 monocytes in paroxetine treated‐HUVECs and vehicle treated‐HUVECs exposed to OSS. j) Oil Red O staining indicating decreased atherosclerotic lesions in paroxetine‐treated ApoE^‐/‐^ mice fed a high‐fat Western diet. k) Quantification of atherosclerotic lesions (n = 12, Student's *t* test). l) H&E and Oil Red O staining of the aortic roots of hyperlipidemic ApoE^‐/‐^ mice in the vehicle group and paroxetine‐treated (10 mg kg^−1^ daily; I.g) group. m,n) Quantification of atherosclerotic lesions (n = 12, Student's *t* test).

Paroxetine, a GRK2 chemical inhibitor, was used in the present study. Immunofluorescence staining, as shown in **Figure** **8**a,b, revealed that, after carotid artery partial ligation, the expression levels of ICAM1 and VCAM1 were lower in the mice that received paroxetine (10 mg kg^−1^, I.g.) than in the vehicle‐treated mice. We also performed in vitro experiments to further elucidate the role of paroxetine in modulating the GRK2/AP‐1 signaling axis under OSS conditions. Considering that paroxetine can inhibit both GRK2 activity and serotonin reuptake in vitro, we further used fluoxetine (10 µmol L^−1^, selective inhibitor of serotonin reuptake) as a control. We found that, unlike paroxetine (10 µmol L^−1^), fluoxetine did not decrease ICAM1 or VCAM1 expression after OSS exposure (Figure 8c–f). As shown in Figure 8g,h, paroxetine treatment significantly reduced GRK2^S29p^, AP‐1^S63p^, and NR4A1 expression levels, indicating a clear inhibitory effect on this signaling cascade. Moreover, paroxetine markedly attenuated the OSS‐induced reduction in LKB1^S428p^, AMPK^T172p^, and eNOS^S1177p^ phosphorylation. These findings confirm that paroxetine effectively suppresses the activation of the GRK2/AP‐1 pathway, thereby halting downstream inflammatory and metabolic responses induced by OSS. The monocyte adhesion results were consistent with the Western blot results, HUVECs treated with paroxetine exhibited decreased monocyte adhesion after OSS stimulation (Figure 8i). To evaluate the therapeutic potential of paroxetine in atherosclerosis, ApoE⁻/⁻ mice were fed a high‐fat Western diet for 12 weeks and treated with paroxetine (10 mg kg^−1^ daily, i.g.). After 12 weeks of high‐fat Western diet feeding, Oil Red O staining revealed that atherosclerotic lesions were reduced in ApoE^‐/‐^ mice treated with paroxetine (10 mg kg^−1^ daily, I.g.) (Figure 8j,k). Consistently, both H&E and Oil Red O staining of the aortic root showed the similar results (Figure 8l–n). Taken together, our results indicate that GRK2 might be a treatment target for OSS‐induced vascular endothelial dysfunction and atherosclerosis.

## 3 Discussion

Vascular endothelial dysfunction is the initial stage of and plays a crucial role in atherosclerosis.^[^ ^17^, ^18^ ^]^ Vascular endothelial shear stress can be calculated using Poiseuille's formula: Vascular endothelial shear stress = 4 µv/πr^3^, “μ” represents the dynamic viscosity of blood, “v” is the blood flow velocity, and “r” is the radius of the blood vessel.^[^ ^3^, ^19^, ^20^ ^]^ Under physiological conditions, laminar flow and LSS activate eNOS, leading to an increase in NO release. Additionally, laminar flow and LSS inhibit mammalian target of rapamycin complex 1 (mTORC1), thereby suppressing vascular smooth muscle cell proliferation.^[^ ^21^ ^]^ These effects are crucial for preventing the development and progression of atherosclerotic diseases.^[^ ^22^, ^23^ ^]^ However, disturbed flow leads to OSS, which actives intracellular signaling cascades and causes vascular endothelial damage. Wu et al., using the same in vitro cell model used in this study, demonstrated that OSS can activate the transcription factor GATA binding protein 6 (GATA6), thereby promoting the transcription and increasing the expression of the chemokine C‐C motif chemokine ligand 5 (CCL5), leading to increased monocyte adhesion. Additionally, GATA6 can promote the transcription and expression of cytidine/uridine monophosphate kinase 2 (CMPK2), which in turn activates the inflammasome NOD‐like receptor family pyrin domain containing 3 (NLRP3), ultimately resulting in inflammation in the vascular endothelium and atherosclerosis.^[^ ^24^ ^]^ Unlike the present study, Wu et al. focused more on the impact of chemokine‐mediated monocyte migration and adhesion on inflammation in the vascular endothelium, whereas our study emphasized the role of the adhesion molecules ICAM1 and VCAM1 in the increased monocyte adhesion induced by OSS in vascular endothelial cells. Quan et al. also conducted a study using the endothelium of the straight segment of the thoracic aorta as the region subjected to LSS and the endothelium at the inner curvature of the aortic arch as the region subjected to OSS. Additionally, their team established the same partial ligation model of the LCA as used in this study. Their results revealed that disturbed flow and OSS can phosphorylate and activate mammalian sterile 20‐like kinase 1 (MST1), which in turn phosphorylates and activates its downstream protein connexin 43 (CX43), leading to conformational changes in CX43 and increased immune cell infiltration, ultimately accelerating the development and progression of atherosclerosis.^[^ ^25^ ^]^ Similar to the present study, their research focused on the mechanisms by which OSS leads to vascular endothelial dysfunction and atherosclerosis. Both studies share similarities: first, the animal and cell models used are comparable, and second, both studies highlight protein kinases as key molecular targets.

In recent years, an increasing number of studies have focused on the non‐canonical functions of GRKs, specifically that GRK2 can directly act on substrates and regulate various downstream signaling pathways through its kinase activity.^[^ ^26^, ^27^ ^]^ Our previous findings indicate that GRK2 plays a role in the pathological process of pulmonary arterial hypertension, where it regulates both the expression and nuclear translocation of YAP in pulmonary vascular smooth muscle cells through non‐canonical mechanisms.^[^ ^28^ ^]^ In fact, in a previous study, researchers explored the effects of OSS on GRK2 in vascular endothelial cells. These findings confirmed that OSS leads to increased GRK2^S29p^ in vascular endothelial cells. Additionally, activated GRK2 under OSS exerts its non‐canonical function by phosphorylating the downstream protein vinculin, thereby damaging vascular endothelial junctions and promoting the development of atherosclerosis.^[^ ^29^ ^]^ However, above study had several limitations. First, the researchers did not measure the levels of GRK2^S29p^ in the endothelium of human atherosclerotic arteries. Additionally, the study lacked detailed in vitro experiments and did not delve deeply into the molecular mechanisms involved. Notably, we constructed vascular endothelial cell‐specific GRK2 knockout mice on an ApoE^‐/‐^ background to investigate whether vascular endothelial cell‐specific knockout of GRK2 could alleviate the development of atherosclerosis. Our results confirmed that vascular endothelial cell‐specific knockout of GRK2 reduces atherosclerosis in ApoE^‐/‐^ mice fed a high‐fat Western diet. Previous studies have also demonstrated that GRK2 in myeloid cells promotes plaque development through immune cell recruitment and inflammatory mediator production.^[^ ^30^ ^]^ Our findings extend this understanding by showing that GRK2 contributes to vascular endothelial dysfunction in response to disturbed blood flow. Notably, treatment with paroxetine, a chemical GRK2 inhibitor, significantly reduced OSS‐induced vascular endothelial dysfunction, suggesting that GRK2 could be a potential target for developing new drugs to treat atherosclerosis in the future. Understanding these differential and potentially interactive effects is critical, as it could open new avenues for targeted therapeutic interventions aimed at modulating GRK2 activity in both vascular and myeloid cells. Future research should investigate the potential crosstalk between vascular endothelial and myeloid cells involving GRK2 signaling, which could provide further insights into molecular mechanisms underlying atherosclerosis and lead to more effective treatment strategies.

To investigate the functional roles of the differentially expressed phosphorylated proteins, we performed functional enrichment analysis on the phosphorylated proteins that were differentially expressed in vascular endothelial cells stimulated by LSS or OSS. The results revealed significant enrichment in biological functions and signaling pathways related to energy metabolism and inflammation. We analyzed the proteins whose expression was most prominently upregulated in vascular endothelial cells under OSS and found a significant increase in the level of AP‐1^S63p^. AP‐1 is a transcription factor known for promoting inflammation and has been shown in previous studies to play a crucial role in the development of cardiovascular inflammation. Since previous research has demonstrated that AP‐1 can promote the transcription of ICAM1 and VCAM1 in vascular endothelial cells, we did not perform ChIP assays or dual‐luciferase reporter assays to further detail its role in promoting the transcription of ICAM1 and VCAM1 in this study.^[^ ^31^, ^32^, ^33^ ^]^ However, we conducted a detailed investigation of the relationship between GRK2 and AP‐1, and our results revealed that AP‐1 can bind to GRK2. Additionally, knockdown GRK2 significantly reduced the increase in phosphorylated AP‐1 levels induced by OSS, indicating that AP‐1 is a downstream target of GRK2 in mediating OSS‐induced endothelial dysfunction.

Functional enrichment analysis suggested that energy metabolism may be affected by OSS in the phosphoproteomic analysis. NR4A1, also known as NUR77, is a member of the nucleus receptor family.^[^ ^34^ ^]^ NR4A1 has been implicated in the regulation of metabolism, mitochondrial function, and immune responses; notably, NR4A1 has been shown to regulate mast cell activation via the LKB1‐AMPK pathway and to modulate cardiac metabolism.^[^ ^35^, ^36^, ^37^ ^]^ LKB1 is a serine/threonine kinase that plays a crucial regulatory role in AMPK activation. The LKB1‐AMPK axis is an important pathway for regulating energy metabolism in cells. LKB1 is located on the inner side of the nuclear membrane, and it must translocate to the cytoplasm to undergo phosphorylation to be activated. Previous studies have shown that NR4A1 can bind to and anchor LKB1 in the nucleus, thereby inactivating the downstream AMPK signaling pathway and ultimately leading to metabolic disorders such as diabetes.^[^ ^36^, ^38^ ^]^ Previous evidence suggested that inactivation of the AMPK signaling pathway can lead to oxidative stress and excessive ROS accumulation in the vascular endothelium.^[^ ^39^, ^40^ ^]^ Additionally, the AMPK signaling pathway is an upstream regulator of eNOS. Abnormalities in vascular endothelial cell energy metabolism can lead to eNOS inactivation, ultimately resulting in reduced NO production in vascular endothelial cells.^[^ ^41^, ^42^ ^]^ In this study, our experimental results indicate for the first time that OSS can increase the expression of NR4A1 in vascular endothelial cells, which, by binding to LKB1 in the nucleus, affects the downstream AMPK signaling pathway and energy metabolism, ultimately leading to vascular endothelial dysfunction, including energy metabolism imbalance, reduced NO production, and oxidative stress. However, NR4A1 exhibits context‐dependent functions, with several previous studies highlighting its anti‐inflammatory and anti‐atherosclerotic roles in other cell types. Our results emphasize its involvement in vascular endothelial dysfunction under disturbed flow conditions. In the context of vascular endothelial cells, NR4A1 acts as a mediator of proatherosclerotic signaling rather than exerting protective effects on macrophages and VSMCs.^[^ ^43^, ^44^, ^45^ ^]^

Seahorse flux analysis is a powerful tool for exploring energy metabolism. However, there is a technical infeasibility in performing Seahorse flux analysis on endothelial cells after shear stress stimulation. Shear stress stimulation needs to be applied using a parallel plate flow chamber system, whereas the Seahorse flux analysis requires cells to be plated in a specialized 96‐well microplate compatible with the Seahorse flux platform. To our knowledge, no study has directly performed Seahorse flux analysis on endothelial cells following exposure to shear stress. A previous study found that LSS may suppress endothelial metabolism through KLF2‐mediated repression of glycolytic enzymes such as PFKFB3.^[^ ^46^ ^]^ Notably, the investigators did not perform Seahorse flux analysis directly on shear stress‐stimulated endothelial cells. Instead, they analyzed static endothelial cells with or without KLF2 overexpression, using the latter as a surrogate for LSS‐induced effects, given that KLF2 is upregulated in response to laminar flow. In the present study, we found that overexpression of GRK2^S29D^, AP‐1^S63D^, and NR4A1, molecules upregulated in endothelial cells under OSS, profoundly suppressed endothelial energy metabolism. Activation of the GRK2/AP‐1/NR4A1 axis markedly reduced glycolytic activity, as evidenced by decreased ECAR, glucose‐stimulated glycolysis, and maximal glycolytic capacity. In addition, this signaling axis significantly impaired mitochondrial respiration, including reductions in basal OCR, ATP production, and respiratory capacity. The simultaneous suppression of glycolysis and mitochondrial oxidative phosphorylation may be attributed to the inhibition of AMPK signaling. AMPK is a central metabolic sensor that promotes ATP‐generating catabolic processes, including stimulation of glycolysis through activation of 6‐phosphofructo‐2‐kinase activity, and enhancement of mitochondrial biogenesis and oxidative phosphorylation.^[^ ^47^, ^48^, ^49^ ^]^ The Seahorse flux analysis findings of this study have clinical implications. Previous study showed that under steady LSS conditions, endothelial cells enter a quiescent state characterized by low metabolism, anti‐inflammation, and low proliferation through the induction of KLF2 expression. This state is thought to help maintain vascular endothelial homeostasis and suppress the development of atherosclerosis, representing an adaptive suppression aimed at reducing the endothelial dependency on energy to maintain functional stability. However, under OSS conditions, we observed activation of the GRK2/AP‐1/NR4A1 signaling axis, which further leads to a reduction in AMPK activity. This metabolic suppression not only fails to maintain homeostasis but also reduces the cell ability to resist environmental stress, promoting endothelial dysfunction. Furthermore, AMPK typically exerts anti‐inflammatory effects in endothelial cells.^[^ ^50^ ^]^ Therefore, the decrease in AMPK activity may be a key mechanism underlying the coexistence of metabolic suppression and inflammatory activation under OSS conditions.

Our study provides several novel insights. First, we revealed that GRK2 serves as a mechanotransducer, activating AP‐1 signaling under disturbed flow, which in turn upregulates the expression of adhesion molecules (ICAM1 and VCAM1) and promotes monocyte recruitment, contributing to inflammation in the vascular endothelium. Second, unlike the previously reported anti‐inflammatory and antiatherosclerotic roles of NR4A1 in macrophages, we are the first to demonstrate that NR4A1 impairs vascular endothelial energy metabolism in the context of disturbed flow‐induced vascular endothelial dysfunction. NR4A1 anchors LKB1 in the nucleus, thereby inhibiting AMPK activation and impairing vascular endothelial cell energy metabolism. Third, we identified the binding site of AP‐1 and its regulatory region on the NR4A1 promoter, further elucidating how disturbed flow amplifies AP‐1‐driven transcriptional activation of NR4A1 and highlighting a previously unexplored relationship between inflammation and metabolic dysfunction in the context of atherosclerosis. Our findings also have several clinical implications. First, owing to the classic mechanosensitive role of GPCRs and the regulation of GPCRs by GRK2, as well as the important role of AP‐1 in inflammatory responses, targeting GRK2/AP‐1 signaling pathway could be a potential therapeutic strategy for preventing or mitigating atherosclerosis, particularly in regions exposed to disturbed flow. Second, NR4A1 and its regulation of LKB1‐AMPK signaling affect vascular endothelial cell metabolism, suggesting that restoring normal metabolic function could be a promising approach to counteract vascular endothelial dysfunction associated with disturbed flow. Third, paroxetine, a GRK2 inhibitor, has protective effects against disturbed flow‐induced vascular endothelial dysfunction and atherosclerosis. Understanding the GRK2/AP‐1/NR4A1 signaling axis in disturbed flow‐induced vascular endothelial dysfunction and atherosclerosis will aid in the development of novel drugs, offering more targeted and effective treatment options for vascular diseases.

## 4 Conclusion

**Figure 9:**
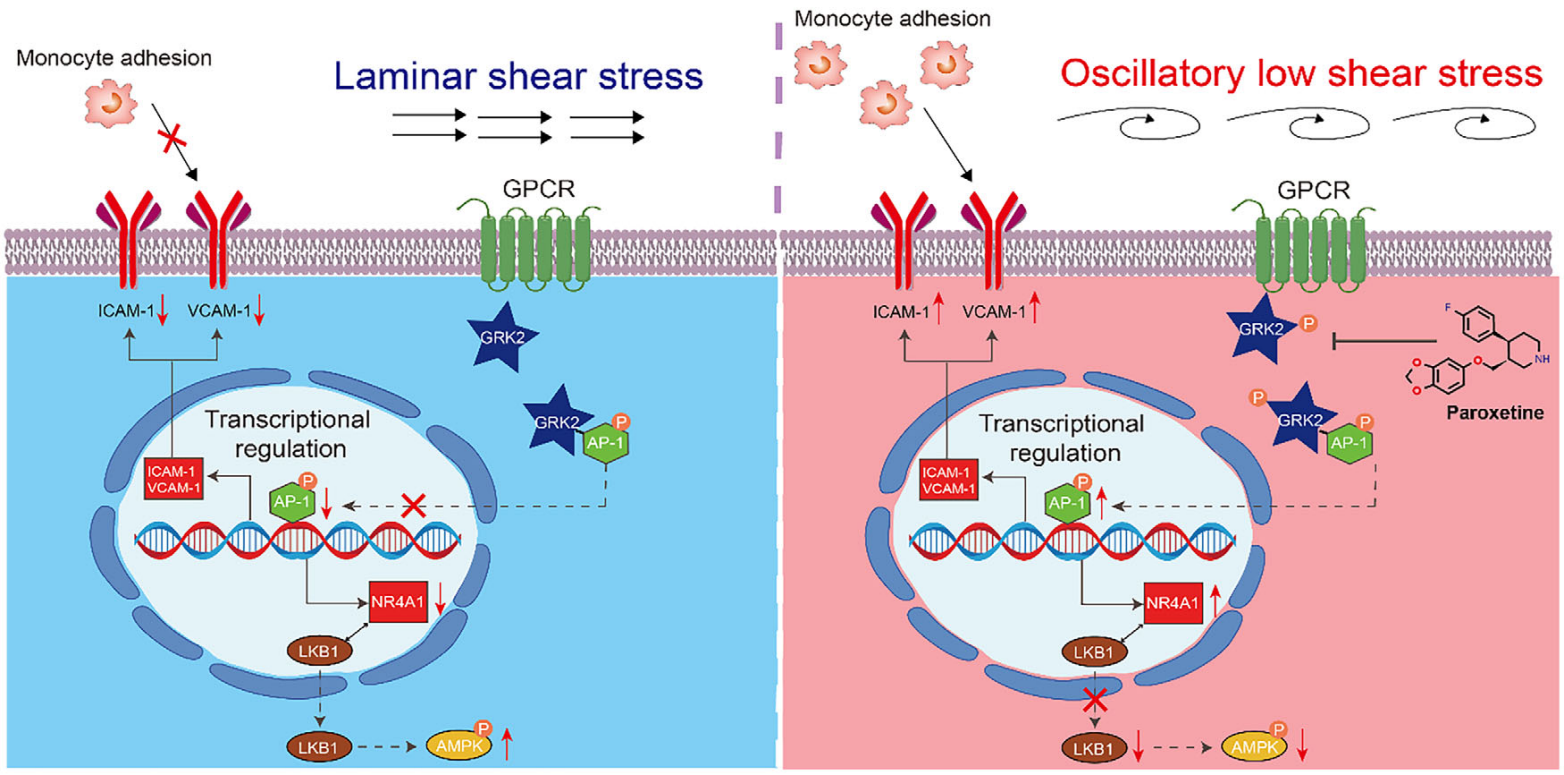
Schematic illustration. OSS activates the GPCR in vascular endothelial cells and leads to the phosphorylation of GRK2, which in turn phosphorylates the downstream transcription factor AP‐1. AP‐1 promotes the transcription of the monocyte recruitment factors ICAM1 and VCAM1, causing inflammation in the vascular endothelium. In addition, AP‐1 also leads to increased expression levels of NR4A1, which binds with LKB1 in the nucleus, resulting in decreased LKB1 phosphorylation and activity. This ultimately leads to AMPK inactivation, oxidative stress, and reductions in eNOS activity.

Our study highlights the pivotal role of GRK2 in mediating OSS‐induced vascular endothelial dysfunction and atherosclerosis. OSS increases the expression level of GRK2^S29p^, and the use of vascular endothelial cell‐specific GRK2 knockout mice and chemical GRK2 inhibitors further supports the potential of targeting GRK2 as a therapeutic strategy for atherosclerosis. Notably, AP‐1^S63p^ was identified as a prominently upregulated phosphoprotein under OSS and was found to be regulated by GRK2, thereby influencing the expression of ICAM1 and VCAM1. Moreover, OSS‐induced AP‐1 activation also increases the expression of NR4A1 in vascular endothelial cells, which affects energy metabolism by anchoring LKB1 within the nucleus and modulating the AMPK signaling pathway, leading to impaired energy metabolism, oxidative stress and reduced NO production (**Figure** **9**).

## 5 Experimental Section

### Atherosclerosis Studies

GRK2^flox/flox^ mice (Stock No. 01 2458, The Jackson Laboratory, Bar Harbor, ME, USA) were interbred with Cdh5‐CRE (Cre recombinase) mice, provided by the Genetic Animal Center at Nanjing University, China. Offspring comprising GRK2^flox/–^ Cdh5‐CRE^+^ mice were then crossed with GRK2^flox/flox^ mice to produce endothelial cell‐specific GRK2 knockout mice (GRK2^ECKO^: GRK2^flox/ flox^ Cdh5‐CRE^+^). All experimental subjects were maintained on C57BL/6J background. After genotyping, littermates were randomly assigned to various experimental groups. GRK2^ECKO^ mice were crossed with ApoE^‐/‐^ mice to generate GRK2^ECKO^; ApoE^‐/‐^ mice. Mice aged 6 to 8 weeks, including both mutants and wild‐type controls, received tamoxifen at a dosage of 100 mg kg^−1^ (intraperitoneal injection), administered 7 consecutive days. GRK2^flox/flox^; ApoE^‐/‐^ mice and GRK2^ECKO^; ApoE^‐/‐^ mice were fed by an 8‐week h high‐fat Western diet, then aortic tissues were harvested for the assessment of atherosclerotic lesions. Cryostat sections of the aortic root were utilized for staining to quantify the area of atherosclerotic lesions and to analyze lesion composition. Moreover, Sudan IV staining was also performed on whole aortas to quantify atherosclerotic lesions. All procedures were conducted in accordance with the guidelines established by the Animal Care and Use Committee of Nanjing Medical University (Ethical approval number: No. DWSY23110414).

### Phosphorproteomic Sequencing

Proteins were extracted from samples using lysis buffer containing protease and phosphatase inhibitors, followed by sonication and centrifugation. After DTT reduction and IAM alkylation, protein concentration was determined by the Bradford assay. SDS‐PAGE was used for quality control. ≈30 µg of protein was separated by gel electrophoresis, excised, destained, and subjected to in‐gel trypsin digestion. Peptides were extracted, dried, and labeled with IBT reagents. Labeled peptides were desalted and enriched for phosphopeptides using TiO₂ affinity chromatography. Eluted phosphopeptides were dried and fractionated into six parts using high‐pH reversed‐phase chromatography. Each fraction was reconstituted and analyzed by nanoLC‐MS/MS on a Thermo UltiMate 3000 UHPLC system coupled to a Q‐Exactive HF‐X mass spectrometer in DDA mode. Peptides were separated on a C18 column using a 60‐min gradient and detected with high resolution. The 20 most intense ions per scan were selected for HCD fragmentation and MS/MS analysis. Dynamic exclusion was set at 30 s. Phosphorproteomics technology has inherent constraints in detecting low‐abundance phosphorylated peptides, in our study, we identified 2609 phosphorylated proteins, which is within the expected detection range for this technique. We did not identify the peptide corresponding to GRK2^S29p^ due to its low abundance. Additionally, we did not detect peptides corresponding to other phosphorylation sites of GRK2.

### Human Carotid Artery Samples

Carotid artery samples were obtained from patients with atherosclerosis undergoing carotid endarterectomy, all patients signed the written informed consent. This study involved only a retrospective analysis of de‐identified tissue samples and did not involve or disclose any personal or identifiable patient information, which was approved by the Ethics Committees of Nanjing First Hospital, and was conducted in accordance with the principles outlined in the Declaration of Helsinki (Ethical approval number: No. Csyayj2024013).

### Ligation of the Left Carotid Artery

To simulate an in vivo disturbed flow model, we performed partial carotid artery ligation, as described previously.^[^ ^16^, ^51^, ^52^, ^53^ ^]^ Briefly, three of the four branches of the LCA, specifically the left external carotid artery, internal carotid artery, and occipital artery, were occluded using 6‐0 silk sutures. The superior thyroid artery was left intact. The RCA served as the control group. Carotid blood flow was assessed 1 day after ligation using the Vevo 2100 ultrasound imaging system (VisualSonics, Toronto, Canada) equipped with a high‐frequency 30 MHz probe.

### Shear Stress Experiments In Vitro

To simulate shear stress conditions in vitro, endothelial cells were initially cultured on glass slides. After transfection or pharmacological treatment, these slides were moved to a parallel plate flow chamber system (ibidi Pump System, #10902‐S, ibidi, Martin Luther, German). The chamber was filled with ECM supplemented with 10% fetal bovine serum to ensure the endothelial cells were fully immersed. A unidirectional flow rate controller was utilized to maintain the desired flow rate, exposing the endothelial cells to either OSS of ±2 dyne cm^−^^2^ at a frequency of 1 Hz or LSS of 15 dyne cm^−^^2^, 0 Hz.

### Histological Staining

Samples were fixed in 4% neutral paraformaldehyde (PFA) and subsequently embedded in paraffin, from which 6 µm thick sections were obtained. H&E staining was performed according to the manufacturer's instructions to assess the severity of atherosclerotic plaque lesions, while Oil Red O staining was utilized to evaluate lipid accumulation within the plaques, following the manufacturer's guidelines. For antigen retrieval, the tissue sections were immersed in a 10 mM sodium citrate buffer (pH 6.0) and subjected to heat in a 100 °C water bath for 20 min. The sections were then permeabilized and blocked with phosphate‐buffered saline (PBS)‐T (0.02% Triton X‐100) containing 1% goat serum for 1 h. Immunostaining involved the application of primary antibodies diluted in PBS‐T, with overnight incubation at 4 °C. Following washing with PBS, the sections were incubated with secondary antibodies, also diluted in PBS‐T (0.02% Triton X‐100), for 1 h at room temperature. After three washes with PBS, the slides were mounted using a medium containing DAPI. Images were captured using confocal microscopy (Carl Zeiss, #LSM 710, Oberkochen, German). Considering that this study focused on endothelial cells, which form the innermost layer of blood vessels, immunofluorescence was chosen as the method for protein expression, which enables the effective analysis of endothelial cells within the context of vascular tissues. All images in same experiments were acquired using the same microscope, exposure time, laser intensity, and gain settings to keep consistency. All samples in same experiments within a given experiment were fixed, permeabilized, blocked, and stained in parallel using the same incubation times to ensure consistency to keep consistency.

### Cell Culture

HUVECs were sourced from the Cell Bank of the Chinese Academy of Sciences (Shanghai, China), while HAECs were obtained from Fuheng Biotechnologies (Shanghai, China). Both types of endothelial cells were maintained in endothelial cell growth medium (ECM) from ScienCell Research Laboratories (#0025, Carlsbad, USA), supplemented with 10% fetal bovine serum (FBS) and 1% penicillin/streptomycin, and incubated at 37 °C in a 5% CO2 environment. Upon reaching 40–60% confluence, cells were transfected with siRNA and plasmids using Lipofectamine 3000 (Thermo Fisher Scientific, #L3000001, Waltham, USA) according to the manufacturer's instructions. To create an in vitro high‐fat milieu, the cells were exposed to 50 µg mL^−1^ of ox‐LDL (Yiyuan Biotechnologies, #YB‐002, Guangzhou, China) for a duration of 48 h. GRK2 inhibitor paroxetine was obtained from Selleck (#S3005, Shanghai, China). The serotonin reuptake inhibitor fluoxetine was purchased from MedChemExpress (#HY‐B0102, Shanghai, China). All siRNAs utilized in these experiments were procured from GenePharma (Shanghai, China), with the specific target siRNA sequences detailed in Table S1, Supporting Information.

### Monocyte Adhesion Assay

The human monocyte cell line THP‐1 (sourced from the Cell Bank of the Chinese Academy of Sciences, Shanghai, China) was cultured in RPMI 1640 medium. Endothelial cells were exposed to either OSS or LSS for a duration of 2 h. Following this, the endothelial cells were labeled with DiI (Yeasen, #40718ES50, Wuhan, Hubei, China) and subjected to centrifugation. They were then washed three times with PBS to remove any excess DiI and resuspended in fresh medium. ≈5 × 10^4^ THP‐1 cells were introduced to the adherent endothelial cells and incubated at 37 °C for 1 h. Non‐adherent cells were subsequently eliminated by washing the samples three times with PBS.

### Macrophage Ox‐LDL Engulfment Assay

RAW macrophages were plated in a 6‐well dish and treated with endothelial cell‐conditioned medium containing ox‐LDL at a concentration of 50 µg mL^−1^ for 4 h. Following the treatment, the cells were fixed in 4% PFA for 10 min and subsequently washed three times with PBS. Then, cells were stained with Oil Red O solution for 20 min in the dark, images were captured using a Leica microscope.

### RNA Isolation and Quantitative Real‐Time PCR

Total RNA was extracted from the harvested endothelial cells using Trizol reagent (Vazyme, #R701, Nanjing, Jiangsu, China). Following the reverse transcription of RNA into complementary DNA (cDNA) with the HiScript III first Strand cDNA Synthesis Kit (Vazyme, #R312‐01/02, Nanjing, Jiangsu, China), relative mRNA expression levels were quantified through quantitative PCR (qPCR). Amplification was conducted using the ChamQ Universal SYBR qPCR Master Mix (Vazyme, #Q711‐02, Nanjing, Jiangsu, China). Gene expression levels were analyzed using the 2−^ΔΔ^CT method, with 18S rRNA employed as the reference gene for normalization. The primer sequences used in this study are provided in Table S2, Supporting Information.

### Western Blotting

Total protein extraction was performed using RIPA buffer supplemented with protease and phosphatase inhibitors. After quantifying the protein concentration, equal amounts (30 µg) were loaded onto a 10% SDS‐PAGE gel and subsequently transferred to a PVDF membrane. The membrane was blocked with 5% non‐fat milk in TBST for 2 h. Following blocking, it was incubated overnight with the appropriate primary antibodies. The membranes were then blocked again using 5% bovine serum albumin in TBST for an additional 2 h and incubated overnight with primary antibodies at optimized dilutions. The antibodies used included GRK2 (Phospho‐Ser29) (EnoGene, #E11‐0486A, New York, New York, USA), GRK2 (Phospho‐Tyr13) (Thermo Fisher, #PA5‐64755, Waltham, Massachusetts, USA), GRK2 (Phospho‐Tyr86) (Thermo Fisher, #PA5‐64756, Waltham, Massachusetts, USA), GRK2 (Phospho‐Ser685) (Immunoway, #YP1225, Newark, Delaware, USA), GRK2 (Phospho‐Ser670) (Affinity Bioscience, #AF3697, Nanjing, Jiangsu, China), GRK2 (Santa Cruz, #sc‐13143, Dallas, Texas, USA), GRK3 (Abclonal, #A9163, Wuhan, Hubei, China), GRK4 (Abclonal, #A10370, Wuhan, Hubei, China), GRK5 (Abclonal, #A3899, Wuhan, Hubei, China), GRK6 (Abclonal, #A6379, Wuhan, Hubei, China), CD31 (Immunoway, # PT0350R, Newark, Delaware, USA), α‐SMA (Immunoway, # YM6566, Newark, Delaware, USA), AP‐1 (Phospho‐Ser63) (Abclonal, #AP0105, Wuhan, Hubei, China), AP‐1 (Abclonal, #A11378, Wuhan, Hubei, China), NR4A1 (Santa Cruz, #sc‐365113, Dallas, Texas, USA), LKB1 (Phospho‐Ser428) (Santa Cruz, #sc‐271924, Dallas, Texas, USA), LKB1 (Santa Cruz, #sc‐32245, Dallas, Texas, USA), AMPK (Phospho‐Tyr172) (Abclonal, #AP1441, Wuhan, Hubei, China), AMPK (Abclonal, #A12718, Wuhan, Hubei, China), VCAM‐1 (Proteintech, #66294‐1‐Ig, Wuhan, Hubei, China), ICAM‐1 (Abclonal, #A19300, Wuhan, Hubei, China), eNOS (Phospho‐Ser1177) (Santa Cruz, #sc‐81510, Dallas, Texas, USA), and GAPDH (Abclonal, #A19056, Wuhan, Hubei, China). After washing with TBST, the membranes were incubated with HRP‐conjugated secondary antibodies (Thermo Fisher, #C31430100, Waltham, Massachusetts, USA) for 2 h at room temperature. Protein bands were visualized using an ECL chemiluminescent reagent (Yeasen, #36208ES60, Nanjing, Jiangsu, China), and band intensities were quantified using Image J software version 1.43.

### ChIP Assays

The binding sites of AP‐1^S63p^ on NR4A1 were identified using the JASPAR database. ChIP assays were performed following the protocol provided in the ChIP assay kit (Abcam, #ab500, Cambridge, UK). Chromatin DNA was extracted from HUVECs, and chromatin cross‐linking was achieved using 1% formaldehyde. Protein‐DNA complexes were subsequently immunoprecipitated with either an AP‐1^S63p^ antibody or control rabbit IgG. Following reverse cross‐linking, PCR amplification was conducted on the immunoprecipitated chromatin samples. The primer sequences for all predicted binding sites are listed in Table S3, Supporting Information.

### Luciferase Reporter Assay

The promoter regions with AP‐1 binding sites on NR4A1 were cloned into the pGL3 promoter luciferase vector. Luciferase activity was assessed 48 h after transfection using a dual luciferase assay kit (Promega, #E1960, Madison, Wisconsin, USA). The measured luciferase activity for each sample was normalized against the corresponding Renilla luciferase activity.

### Constructing of ICAM2 Promoter‐Driven AAV9 for Endothelial‐Specific Overexpression of AP‐1^S63D^ and GRK2^S29D^ In Vivo

To facilitate endothelial‐specific overexpression of AP‐1^S63D^ and GRK2^S29D^ in vivo, an AAV9 was engineered utilizing the ICAM2 promoter. This plasmid, containing AP‐1^S63D^ or GRK2^S29D^ fused with GFP, was created by substituting the CMV promoter in the AAV9‐CMV‐GFP‐FT2A‐MCS‐WPRE‐SV40‐PolyA vector with the mouse ICAM2 promoter, which was synthesized by GeneChem (http://www.genechem.com.cn, Nanjing, China). AAV9, at a concentration of 10¹^2^ vg mL^−1^, was administered through tail vein injection.

### Measurement of Intracellular ROS

Intracellular ROS levels were measured using the fluorescent probe 2′,7′‐dichlorodihydrofluorescein diacetate (DCFH‐DA, Beyotime, #S0033S, Shanghai, China). Briefly, cells were incubated with 10 µmol L^−1^ DCFH‐DA diluted in serum‐free medium at 37 °C for 30 min in the dark. After incubation, the cells were washed three times with phosphate‐buffered saline (PBS) to remove excess probe. Fluorescence intensity was then analyzed using a fluorescence microscope. The relative ROS levels were quantified based on the fluorescence intensity.

### Seahorse Flux Analysis

To assess cellular metabolic activity, HUVECs were seeded in fibronectin‐coated Seahorse XF96 cell culture microplates (Agilent Technologies) and incubated overnight. Prior to analysis, the culture medium was replaced with unbuffered DMEM assay medium, and cells were equilibrated for 1 h at 37 °C in a non‐CO₂ incubator. Metabolic parameters were then assessed using the Seahorse XFe96 Extracellular Flux Analyzer. The following compounds were sequentially injected at indicated concentrations to probe specific metabolic functions: glucose (10 mmol L^−1^), oligomycin (3 µmol L^−1^), 2‐deoxy‐D‐glucose (2‐DG, 100 mmol L^−1^), FCCP (1 µmol L^−1^), antimycin A (1.5 µmol L^−1^), and rotenone (3 µmol L^−1^). All metabolic data (OCR and ECAR) were normalized 10⁴ cells.

### Statistical Analysis

Statistical analyses were performed using GraphPad Prism 8.0 software. Results are expressed as mean ± SEM. For comparisons between two groups, an unpaired Student's *t* test was utilized, while one‐way analysis of variance (ANOVA) was employed for multiple group comparisons. Multiple comparisons made using the Bonferroni post‐hoc test. A two‐side *p*‐value less than 0.05 was considered statistically significant.

## Conflict of Interest

The authors declare no conflict of interest.

## Author Contributions

L.D.W., Y.S., and C.H.K. contributed equally to this work, leading the experimental design, data acquisition, and initial manuscript preparation. A.Q.C., Y.K., and J.Y.K. were involved in critical data collection and analysis, providing essential insights into data interpretation. X.M.J. and D.C.W. assisted in developing the experimental methodology and conducted specialized assays. P.C., Y.S., F.W., Y.F.L., and Z.Y.Q. contributed to data analysis and statistical support, ensuring rigor in the interpretation of results. Z.H.J., Y.W.C., and W.Y.Z. supported experimental procedures and conducted technical validation. Y.G. provided additional analytical tools and assisted in data visualization. J.X.Z. and S.L.C. supervised the project, contributed to the study's conception, and provided final manuscript revisions. All authors reviewed, approved the final manuscript, and agreed to be accountable for their contributions.

## Supporting information



Supporting Information

## Data Availability

The data that support the findings of this study are available from the corresponding author upon reasonable request.
