# Supplementary material for: The GRK2/AP‐1 Signaling Axis Mediates Vascular Endothelial Dysfunction and Atherosclerosis Induced by Oscillatory Low Shear Stress
Source: Adv Sci (Weinh). 2025 Jun 10;12(33):e01981. doi: 10.1002/advs.202501981 (PMC12412516; doi:10.1002/advs.202501981)
Supplement: Supplementary file 1 — Supporting Information [file ADVS-12-e01981-s001.docx]

Supporting Information

**The GRK2/AP-1 Signaling Axis Mediates Vascular Endothelial Dysfunction and Atherosclerosis Induced by Oscillatory Low Shear Stress**

*Li-Da Wu, Yi Shi, Chao-Hua Kong, Ai-Qun Chen, Ying Kang, Jun-Yan Kan, Xiao-Min Jiang, Peng Chu, Dong-Chen Wang, Yi-Fei Lv, Zhi-Yuan Qian, Zi-Hao Jiang, Yun-Wei Chen, Yue Sun, Rui-Rui Chang, Wen-Ying Zhou, Yue Gu, Jun-Xia Zhang^*^, Shao-Liang Chen^*^*

**Contents**

[**Figure S1. Schematic diagram illustrating the procedure of partial ligation of the LCA.**. 4](#_Toc196810202)

[**Figure S2. Effects of OSS on GRK2 phosphorylation levels at different phosphorylation sites.**. 5](#_Toc196810203)

[**Figure S3. Effects of OSS on ICAM1 and VCAM1 expression levels.**. 6](#_Toc196810204)

[**Figure S4. Quantification of GRK2^S29p^, ICAM1, and VCAM1 protein expression level in HAECs following oxLDL treatment.**. 7](#_Toc196810205)

[**Figure S5. Construction of endothelial cell-specific GRK2 knockout (GRK2^ECKO^) mice.**. 8](#_Toc196810206)

[**Figure S6. Validation of the GRK2 siRNA efficiency.**. 10](#_Toc196810207)

[**Figure S7. Mass spectrometry (MS) of AP-1 in GRK2 antibody co-immunoprecipitated proteins.** 11](#_Toc196810208)

[**Figure S8. Validation of the AP-1 siRNA efficiency.**. 12](#_Toc196810209)

[**Figure S9. AP-1 phosphorylation status modulates endothelial inflammatory activation and monocyte adhesion under laminar shear stress.**. 13](#_Toc196810210)

[**Figure S10. Endothelial-specific overexpression of GRK2^S29D^ via ICAM-2 promoter-driven AAV9 promotes vascular inflammation and accelerates atherosclerosis progression.**. 14](#_Toc196810211)

[**Figure 11. Effects of GRK2 knockdown on NR4A1 expression levels in HUVECs subject to different shear stress.**. 15](#_Toc196810212)

[**Figure S12. Low shear stress decreased LKB1^S428p^ expression in HAECs.**. 16](#_Toc196810213)

[**Figure S13. Effects of GRK2 deficiency on energy metabolism in HUVECs and HAECs subject to OSS.**. 17](#_Toc196810214)

[**Figure S14. Effects of GRK2 deficiency on ROS levels in HUVECs subject to different shear stress.**. 18](#_Toc196810215)

[**Figure S15. Effects of AP-1 knockdown on energy metabolism in endothelial cells subject to OSS.**. 19](#_Toc196810216)

[**Figure S16. Effects of overexpression of GRK2^S29D^ on AP-1^S63p^, NR4A1, LKB1^S428p^, and AMPK^T172p^ levels in HUVECs under LSS environment.**. 20](#_Toc196810217)

[**Table S1. Sequence information of siRNAs used in RNA knock down assay.** 21](#_Toc196810218)

[**Table S2. Primer sequences for reverse transcript quantitative PCR (RT-qPCR)** 21](#_Toc196810219)

[**Table S3. Primer sequences for qPCR of chromatin immunoprecipitation (ChIP) assays** 21](#_Toc196810220)

[**Table S4. Nucleic acid and amino acid sequences of GRK2^S29D^, GRK2^S29A^ and GRK2^K220R^.** 22](#_Toc196810221)

[**Table S5. Nucleic acid and amino acid sequences of AP-1^S63D^ and AP-1^S63A^.** 27](#_Toc196810222)

[**Table S6. Nucleic acid and amino acid sequences of NR4A1.** 29](#_Toc196810223)

[**Table S7. Nucleic acid and amino acid sequences of LKB1^S428D^.** 31](#_Toc196810224)

[**Table S8. Nucleic acid sequences of Truncated NR4A1 Promoter.** 32](#_Toc196810225)

[**Table S9. Nucleic acid sequences of Mutant NR4A1 Promoter.** 36](#_Toc196810226)

[**Table S10. Body weight and plasma cholesterol of GRK2^ECKO^; ApoE^-/-^ hyperlipidemia and littermate wild-type mice.** 38](#_Toc196810227)

[**Table 11. Body weight and plasma cholesterol of high-fat Western diet fed GRK2^ECKO^; ApoE^-/-^** **mice administered AAV oe-AP-1^S63D^ and AAV vector.** 38](#_Toc196810228)

[**Table 12. Body weight and plasma cholesterol of high-fat Western diet fed ApoE^-/-^** **mice administered AAV oe-GRK2^S29D^ and AAV vector.** 39](#_Toc196810229)


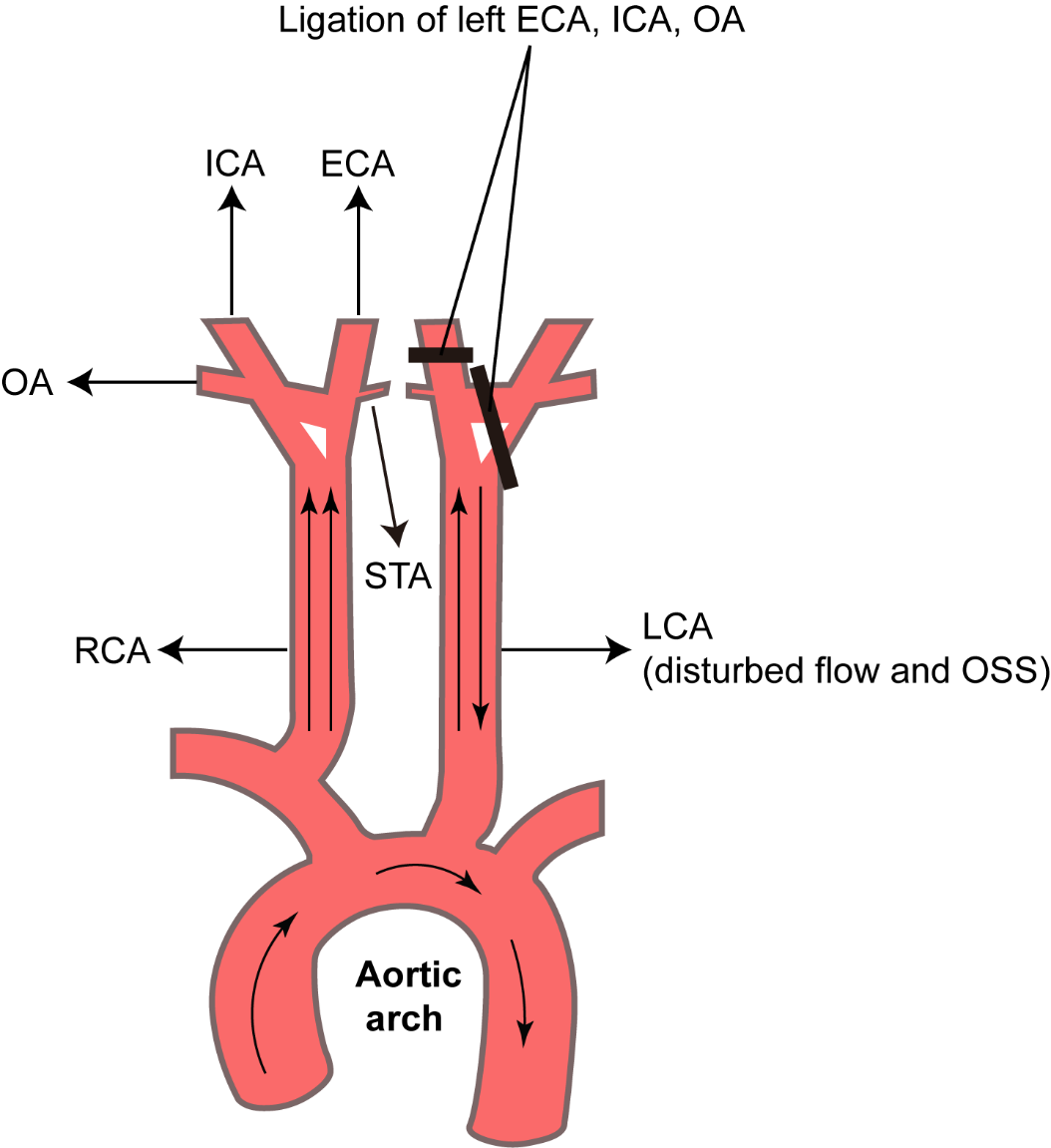


**Figure S1. Schematic diagram illustrating** **the procedure of partial ligation of the LCA.** Partial ligation of the LCA was performed by ligating three of the four caudal branches (ECA, ICA, and OA), while leaving the STA patent. This procedure induces disturbed blood flow in the LCA, resulting in a state of OSS. RCA, right common carotid artery; LCA, left common carotid artery; ECA, external carotid artery; ICA, internal carotid artery; OA, occipital artery; STA, superior thyroid artery.


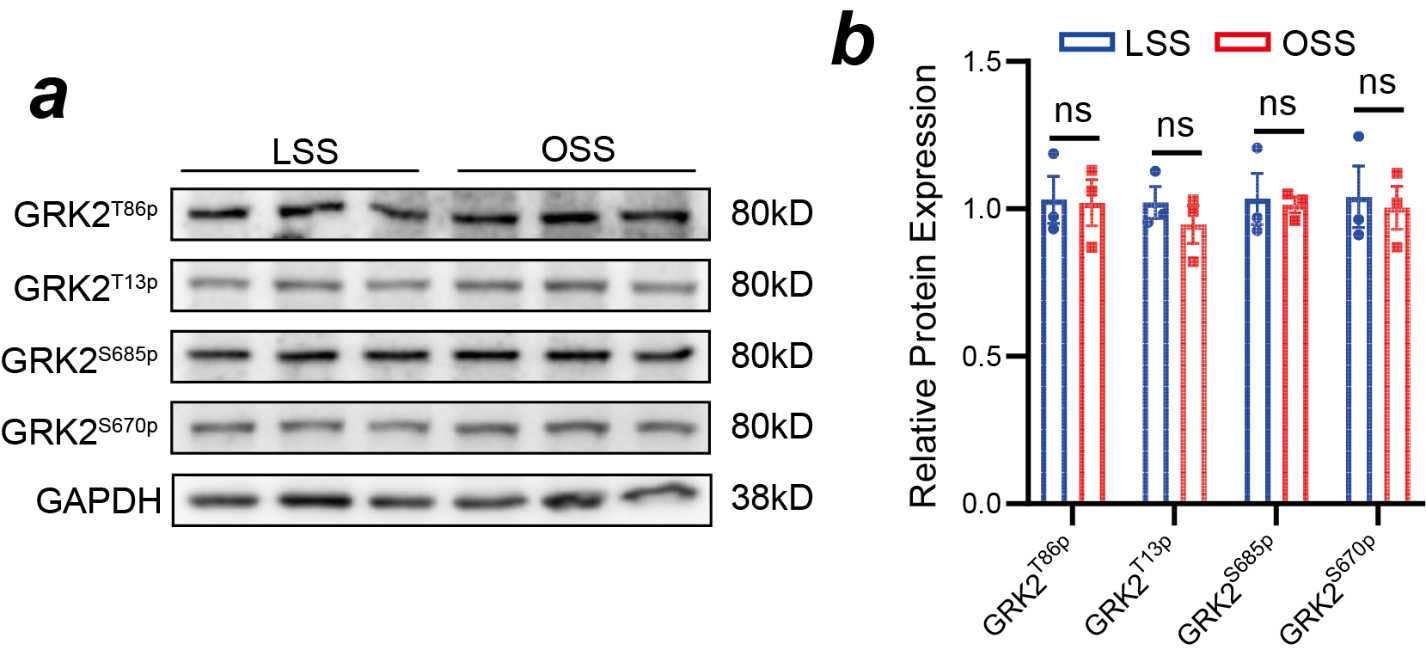


**Figure S2. Effects of OSS on GRK2 phosphorylation levels at different phosphorylation sites.** (a) HUVECs were subjected to LSS (15 dyne/cm^2^, 0 Hz) and OSS (±2 dyne/cm^2^, 1 Hz) for 2 hours. The cells lysate was detected by Western blot with indicated GRK2 antibodies with different phosphorylation sites. (b) Quantification of phosphorylated GRK2 protein expression level (n=3, Student’s t test).


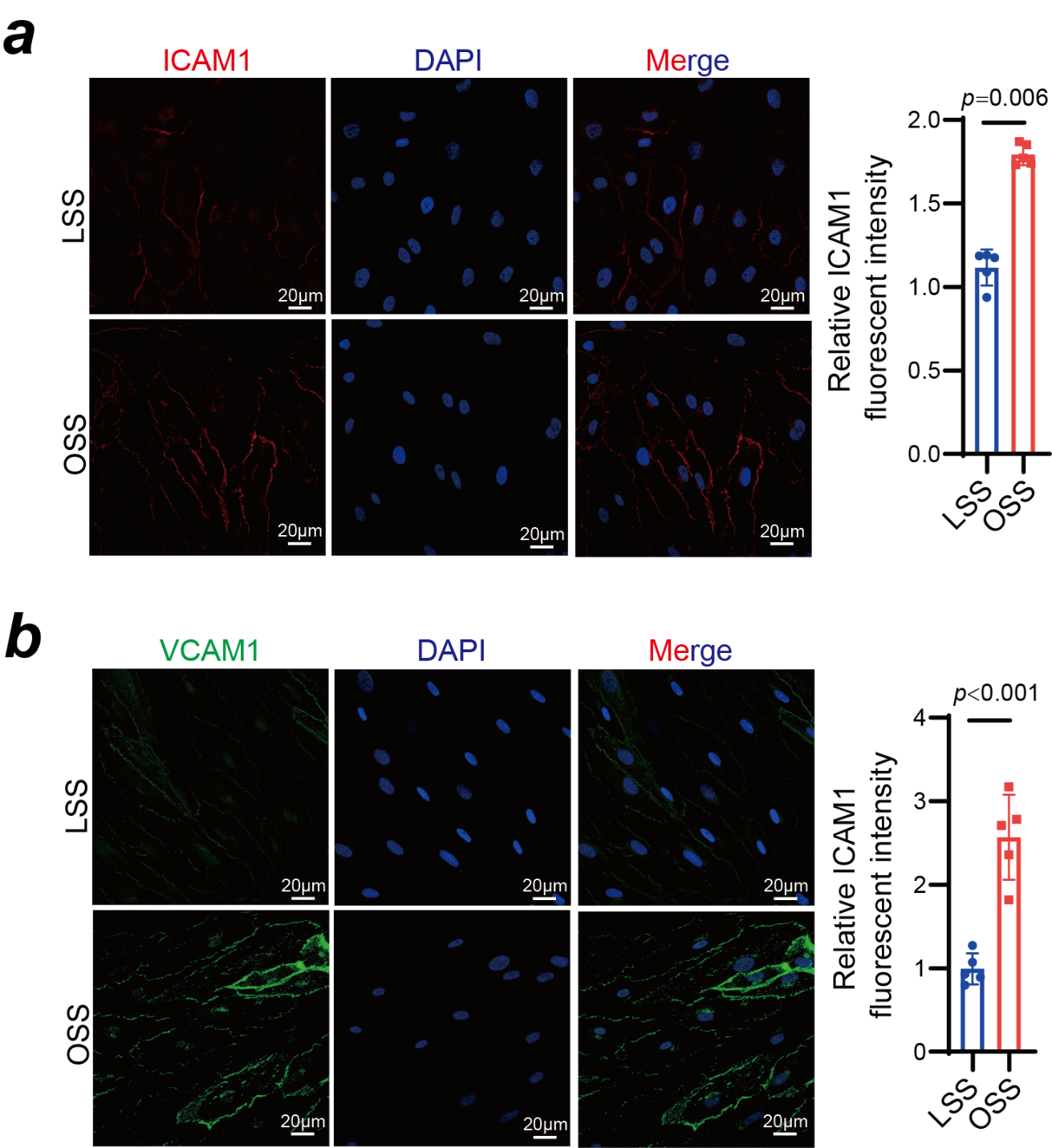


**Figure S3. Effects of OSS on ICAM1 and VCAM1 expression levels.** Immunofluorescence staining for ICAM1 (red) (a), VCAM1 (green) (b), and DAPI (blue) in HUVECs subjected to LSS (15 dyne/cm^2^, 0 Hz) and OSS (±2 dyne/cm^2^, 1 Hz) for 2 hours. Quantification of relative ICAM1 and VCAM1 fluorescent intensity (n=5, Student’s t test) on the right side.


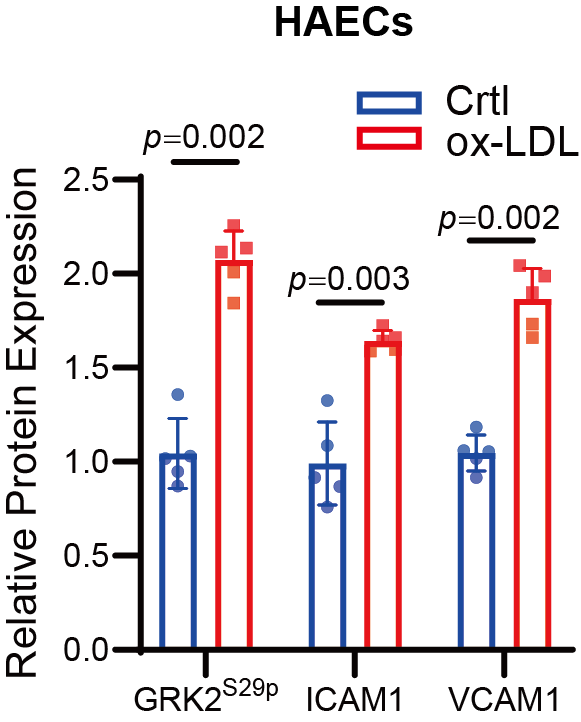


**Figure S4. Quantification of GRK2^S29p^, ICAM1, and VCAM1 protein expression level in HAECs following oxLDL treatment.** HAECs were treated with oxidized low-density lipoprotein (50 g/ml for 48hr), the lysate was detected by Western blot with indicated antibodies (n=5, Student’s t test).


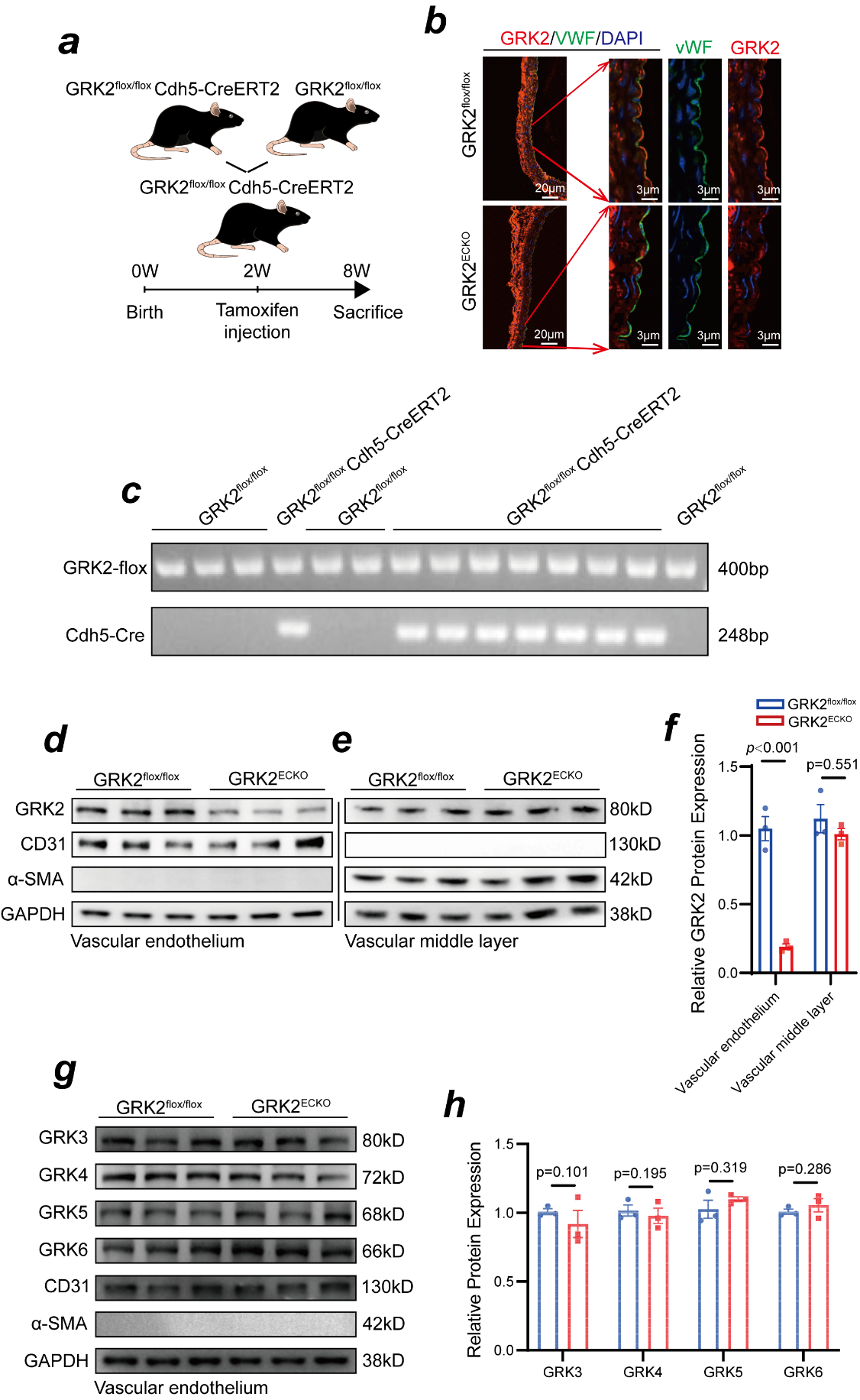


**Figure S5. Construction of endothelial cell-specific GRK2 knockout (GRK2^ECKO^) mice.** (a) Schematic of the transgenic mice used to breed endothelial cell-specific GRK2 knockout (GRK2^ECKO^) mice. (b) Immunofluorescence staining for GRK2 (red), vWF (green), and DAPI (blue) in common carotid artery isolated from GRK2^flox/flox^ mice and GRK2^ECKO^ mice. (c) Genotypic identification of GRK2^ECKO^ mice. (d–e) Western blot of GRK2 expression in endothelium and the medial layer (VSMCs) of endothelium-denuded aortas from GRK2^flox/flox^ mice and GRK2^ECKO^ mice. (f) Quantification of GRK2 protein expression levels (n=3, Student’s t test). (g) Western blot of GRK3, GRK4, GRK5, and GRK6 expression in isolated endothelium from GRK2^flox/flox^ mice and GRK2^ECKO^ mice. (h) Quantification of GRK3, GRK4, GRK5, and GRK6 protein expression levels (n=3, Student’s t test).


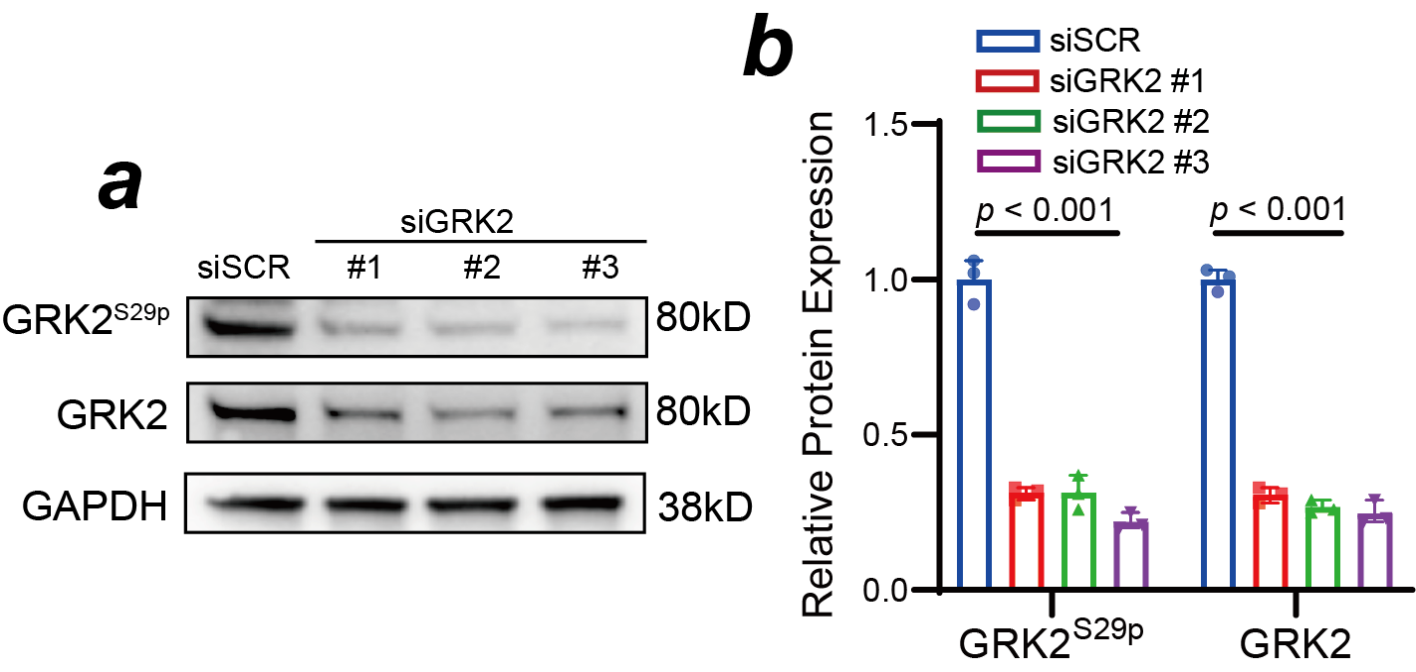


**Figure S6. Validation of the GRK2 siRNA efficiency.** (a) GRK2^S29p^ and GRK2 were detected by Western blot in 3 different GRK2 siRNA treated-HUVECs and scramble siRNA treated-HUVECs. (b) Quantification of GRK2^S29p^ and GRK2 protein expression levels (n=3, Student’s t test).


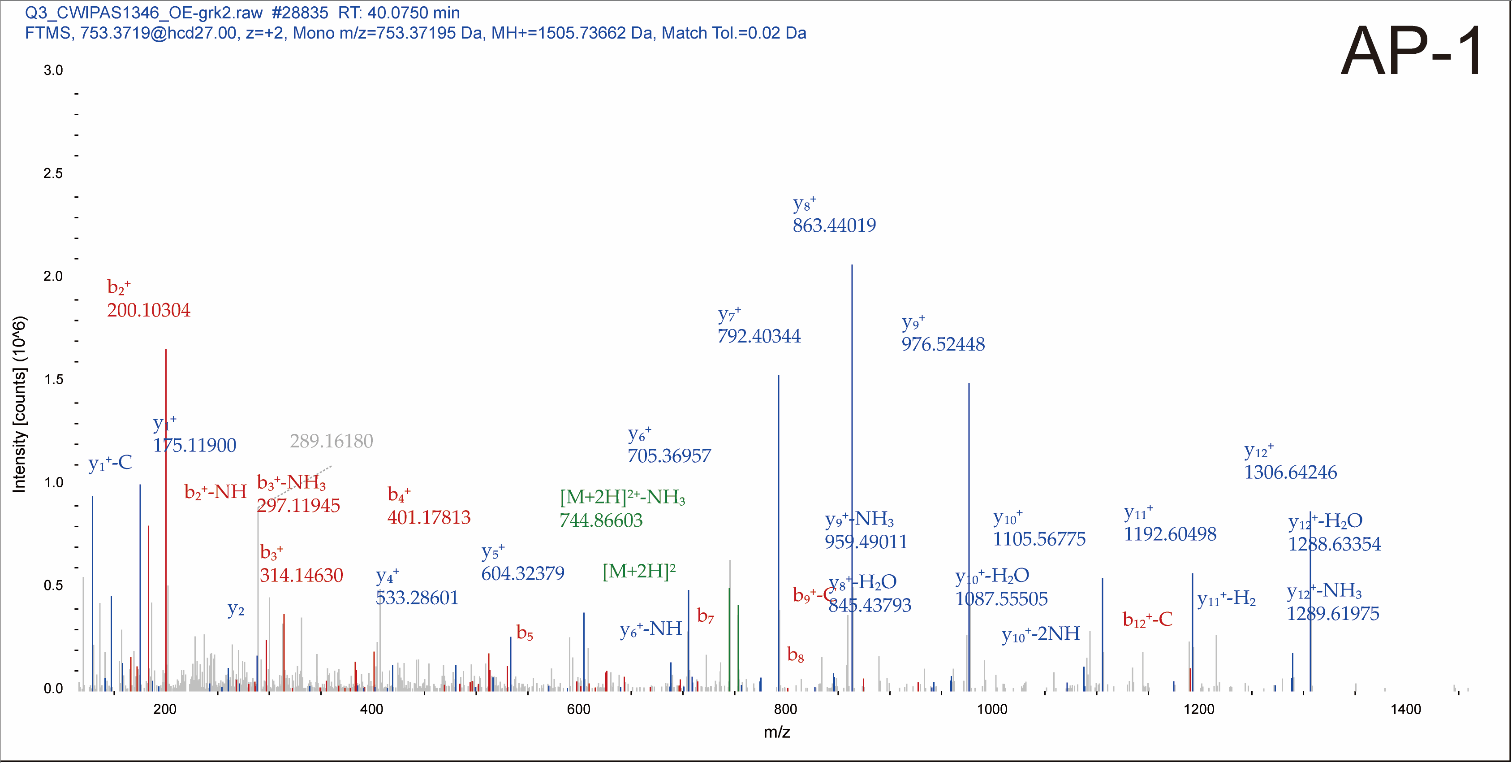


**Figure S7. Mass spectrometry (MS) of AP-1 in GRK2 antibody co-immunoprecipitated proteins.**


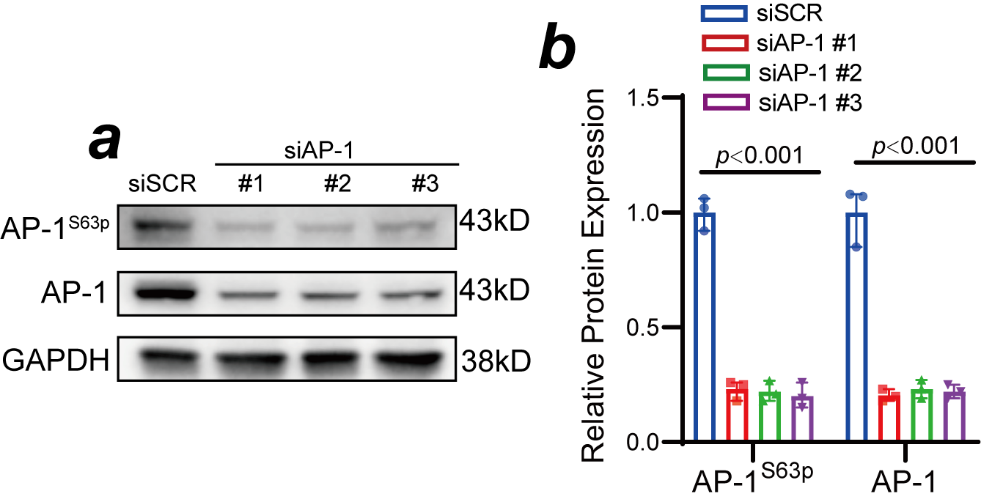


**Figure S8. Validation of the AP-1 siRNA efficiency.** (a) AP-1^S63p^ and AP-1 were detected by Western blot in 3 different AP-1 siRNA treated-HUVECs and scramble siRNA treated-HUVECs. (b) Quantification of AP-1^S63p^ and AP-1 protein expression levels (n=3, one-way ANOVA).


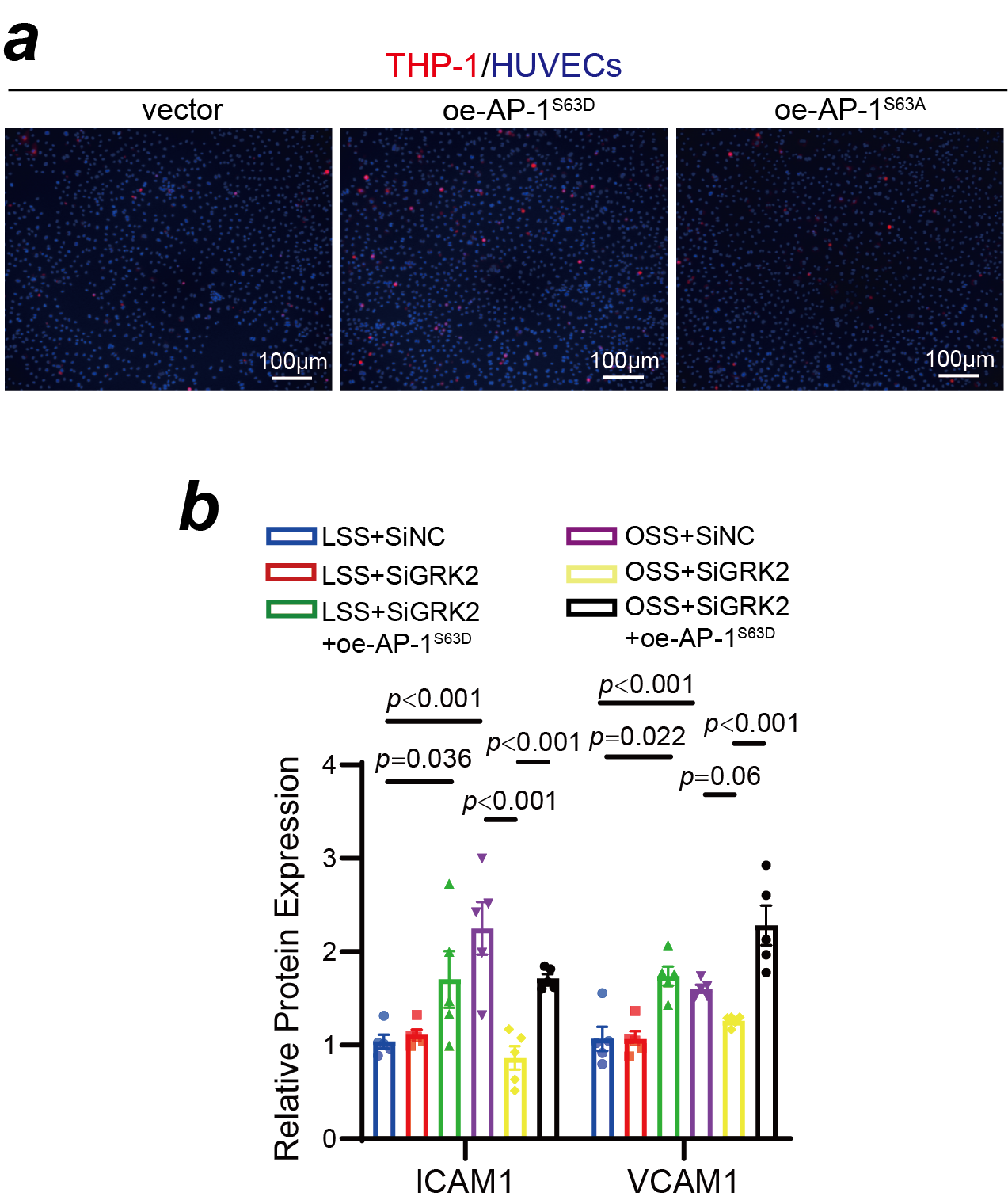


**Figure S9. AP-1 phosphorylation status modulates endothelial inflammatory activation and monocyte adhesion under laminar shear stress.** (a) Adhesion of THP-1 monocytes in sustain activated AP-1^S63D^ overexpressed HUVECs and sustain inactivated AP-1^S63A^ overexpressed HUVECs exposure to LSS (n=5). (b) Quantification of ICAM1 and VCAM1 protein expression levels in rescue Western blot experiments conducted in HAECs (n=5, one-way ANOVA).


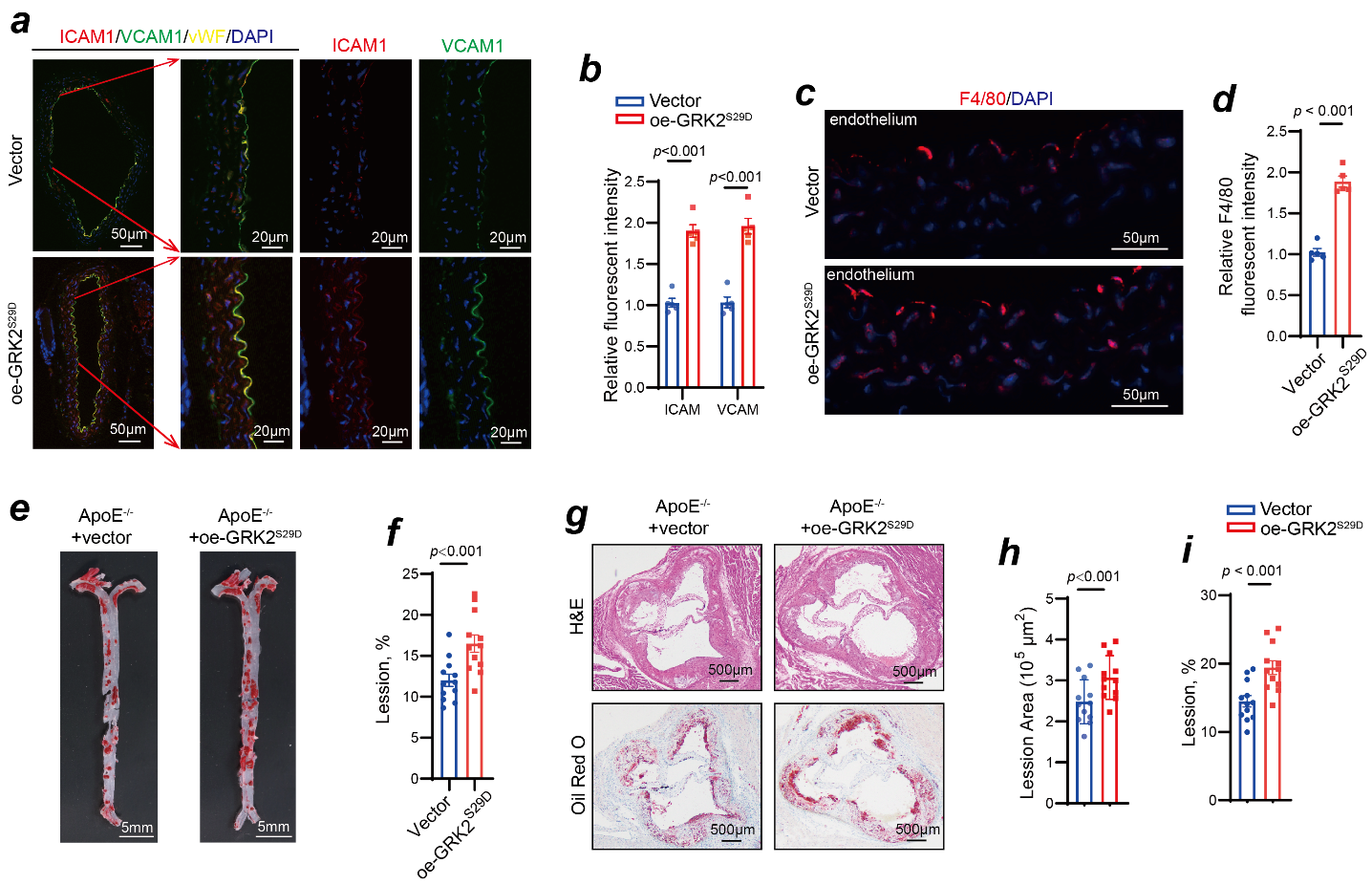


**Figure S10. Endothelial-specific overexpression of GRK2^S29D^ via ICAM-2 promoter-driven AAV9 promotes vascular inflammation and accelerates atherosclerosis progression.** (a) Immunofluorescence staining of ICAM1 (red), VCAM1 (green), vWF (yellow), and DAPI (blue) in carotid arteries from mice injected with AAV9-GRK2^S29D^ or vector. (d) Quantification of ICAM1 and VCAM1 expression levels (n=5, Student’s *t* test). (e) Immunofluorescence staining for F4/80 (red) and DAPI (blue) in carotid arteries indicating macrophage infiltration. (f) Quantification of relative F4/80 fluorescence intensity (n=5, Student’s *t* test). (g) H&E and Oil Red O staining of aortic arch showing increased plaque formation in ApoE^-/-^ mice with endothelial GRK2^S29D^ overexpression. (h-i) Quantification of atherosclerotic lesion area in aortic arch (n=12, Student’s *t* test).


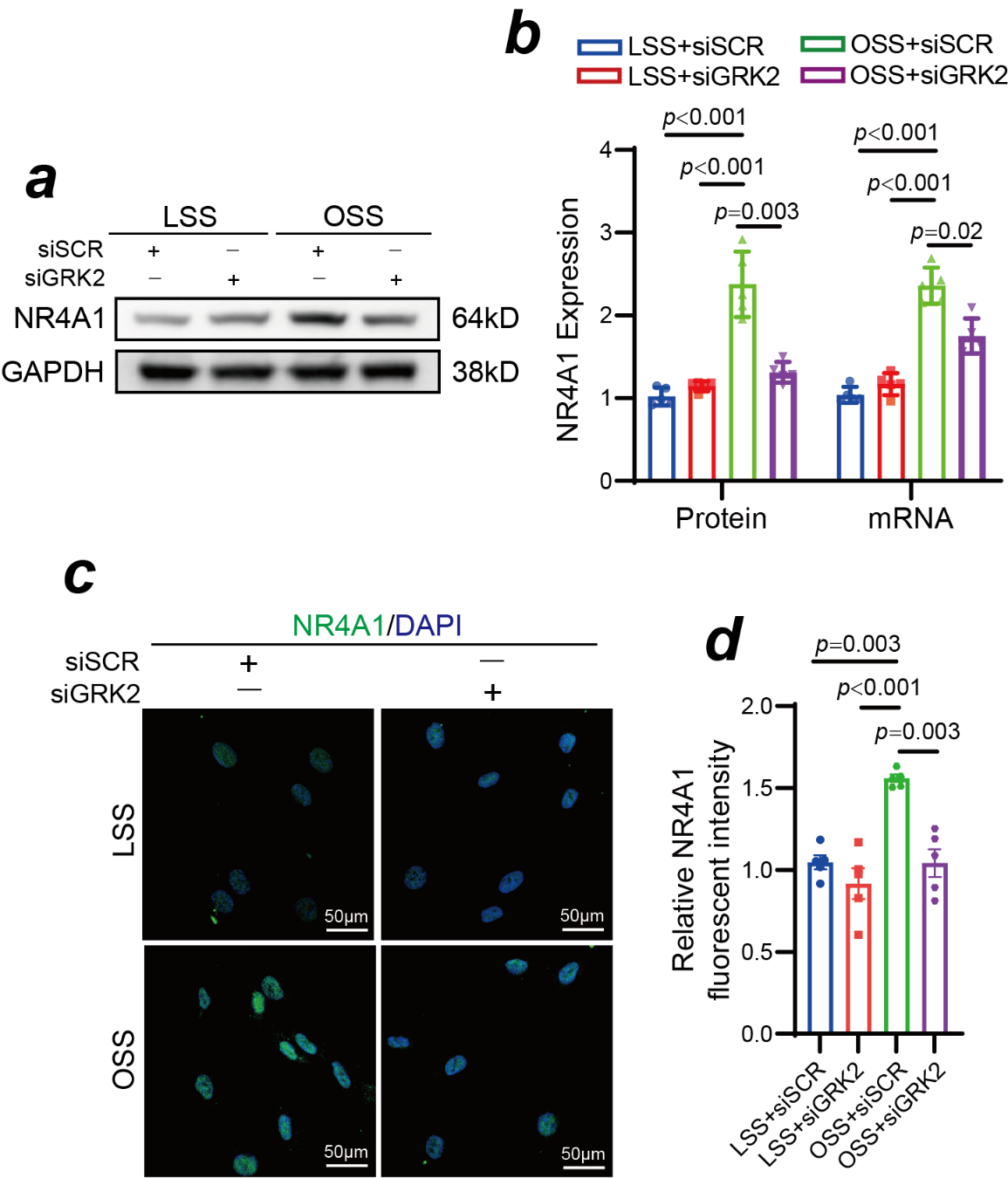


**Figure 11. Effects of GRK2 knockdown on NR4A1 expression levels in HUVECs subject to different shear stress.** (a) GRK2 siRNA treated HUVECs and scramble siRNA treated HUVECs were subjected to LSS (15 dyne/cm^2^, 0 Hz) and OSS (±2 dyne/cm^2^, 1 Hz) for 120 min. The cells lysate was detected by Western blot with indicated NR4A1 antibodies. (b) Quantification of NR4A1 protein and mRNA expression level (n=5, one-way ANOVA). (c) Immunofluorescence staining for NR4A1 (green) and DAPI (blue) in GRK2 siRNA treated-HUVECs and scramble siRNA treated-HUVECs exposure to different shear stress. (d) Quantification of NR4A1 fluorescent intensity level (n=5, one-way ANOVA).


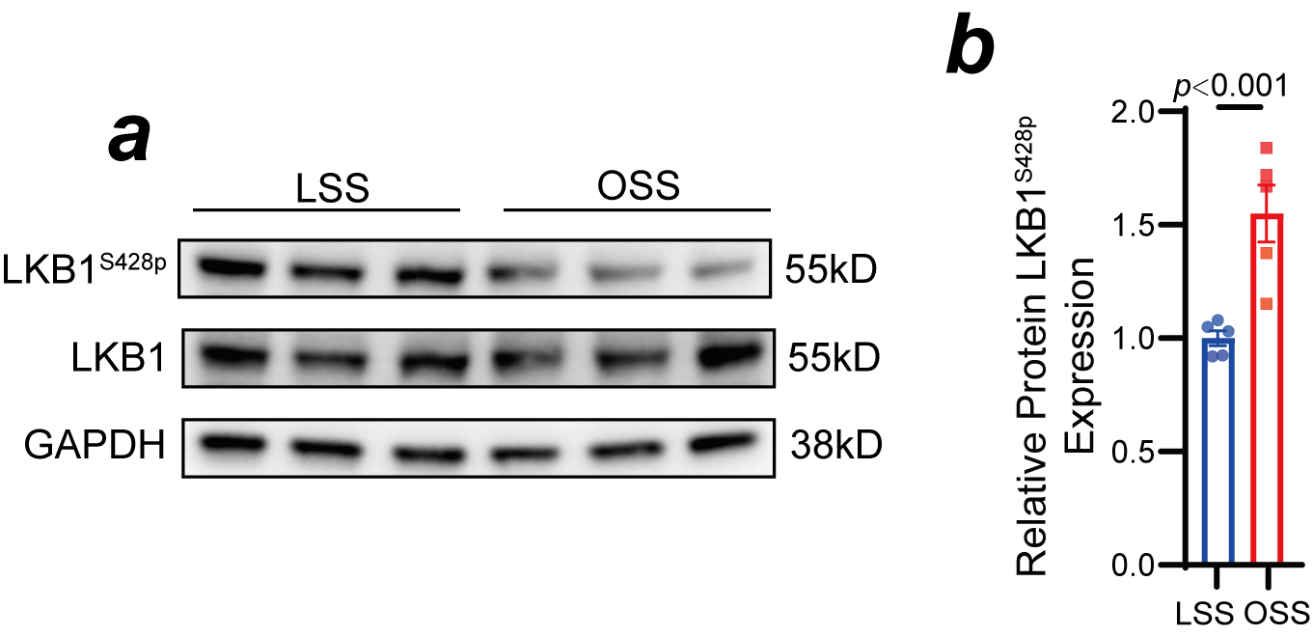


**Figure S12. Low shear stress decreased LKB1^S428p^ expression in HAECs.** (a) HAECs were subjected to LSS (15 dyne/cm^2^, 0 Hz) and OSS (±2 dyne/cm^2^, 1 Hz) for 2 hours. LKB1^S428p^ and LKB1 protein expression levels were detected by Western blot. (b) Quantification of phosphorylated LKB1^S428p^ protein expression level (n=5, Student’s t test).


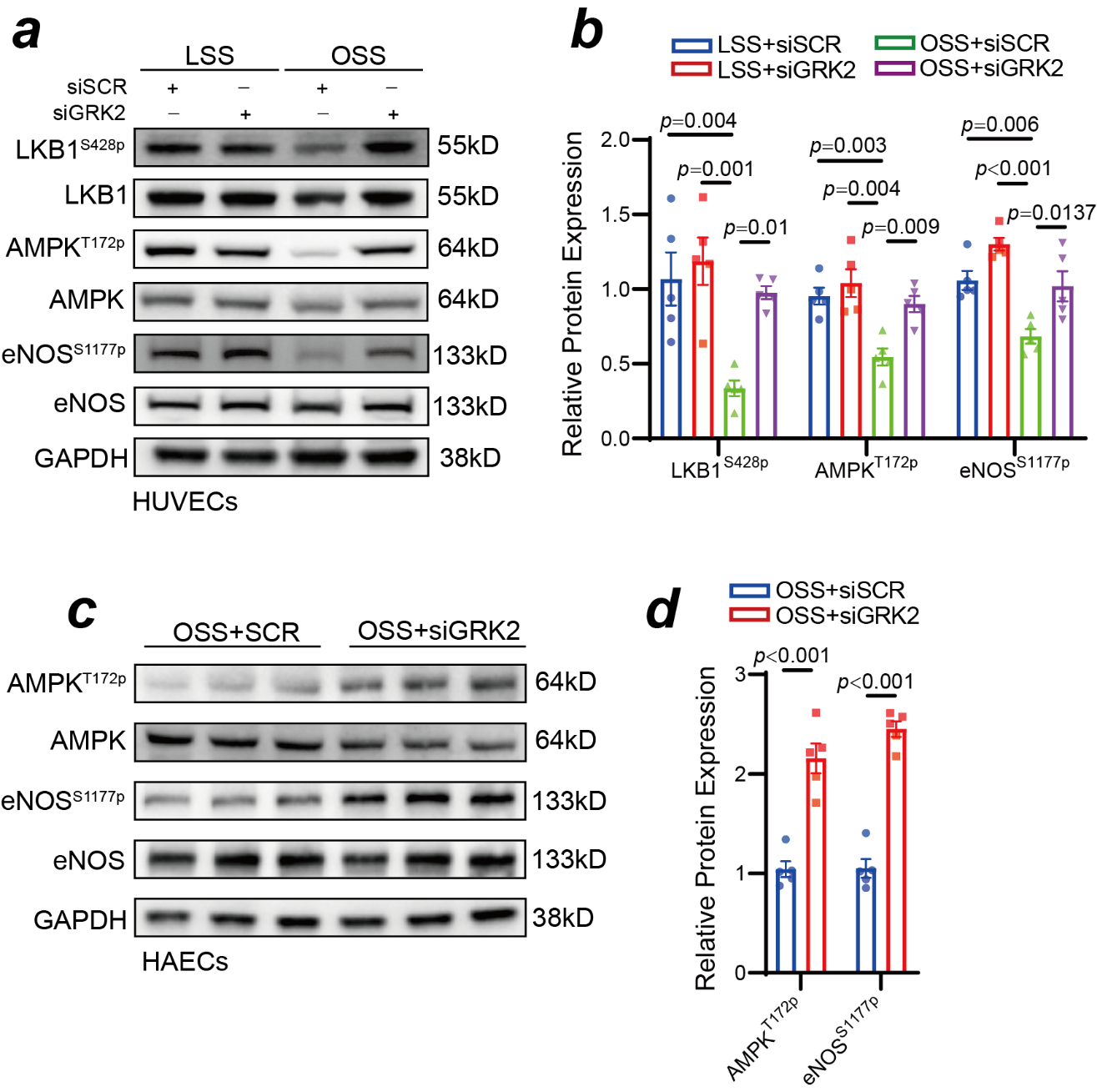


**Figure S13. Effects of GRK2 deficiency on energy metabolism in HUVECs and HAECs subject to OSS.** (a) GRK2 siRNA treated-HUVECs and scramble siRNA treated-HUVECs were exposure to different shear stress, LKB1^S428p^, LKB1, AMPK^T172p^, AMPK, eNOS^S1177p^, and eNOS protein expression levels were detected by Western blot. (b) Quantification of LKB1^S428p^, AMPK^T172p^, eNOS^S1177p^ protein expression levels (n=5, one-way ANOVA). (c) GRK2 siRNA treated-HUVECs and scramble siRNA treated-HUVECs were exposure to OSS, AMPK^T172p^, AMPK, eNOS^S1177p^, and eNOS protein expression levels were detected by Western blot. (d) Quantification of AMPK^T172p^, eNOS^S1177p^ protein expression levels (n=5, Student’s t test).


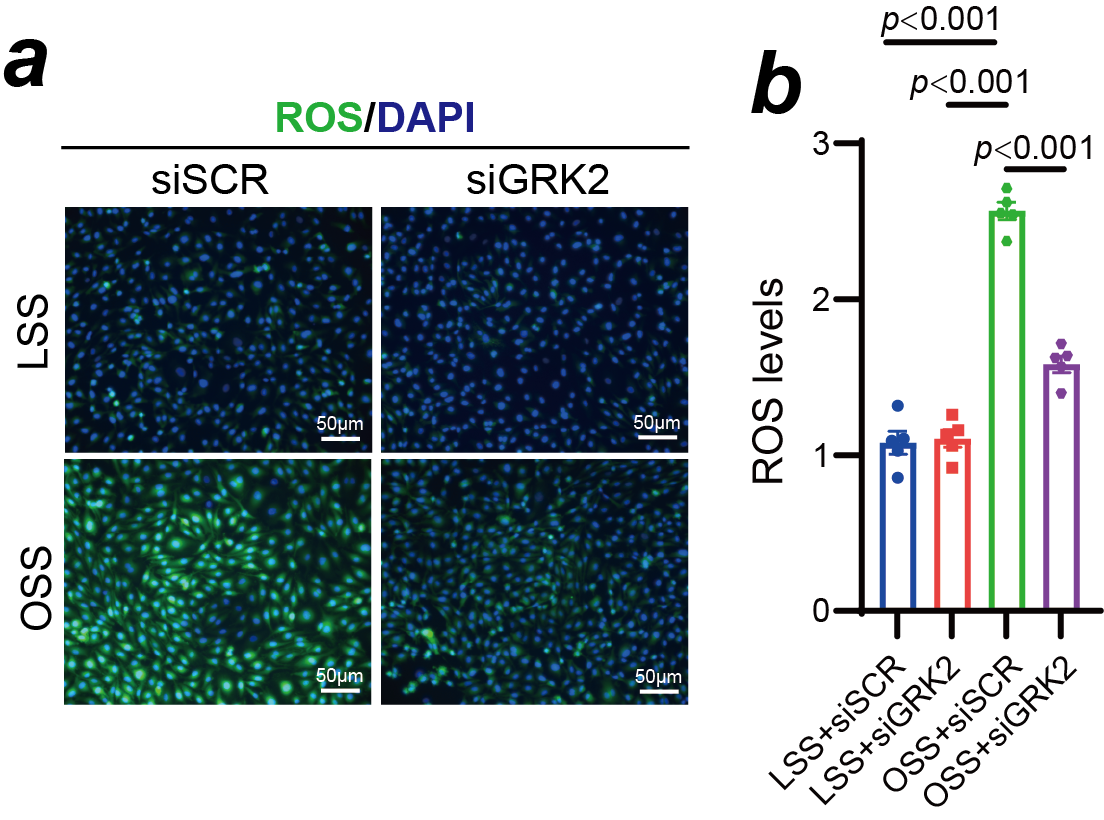


**Figure S14. Effects of GRK2 deficiency on ROS levels in HUVECs subject to different shear stress.** (a) GRK2 siRNA treated-HUVECs and scramble siRNA treated-HUVECs were exposure to different shear stress, ROS levels were detected by DCFH-DA (green). (b) Quantification of ROS levels (n=5, one-way ANOVA).


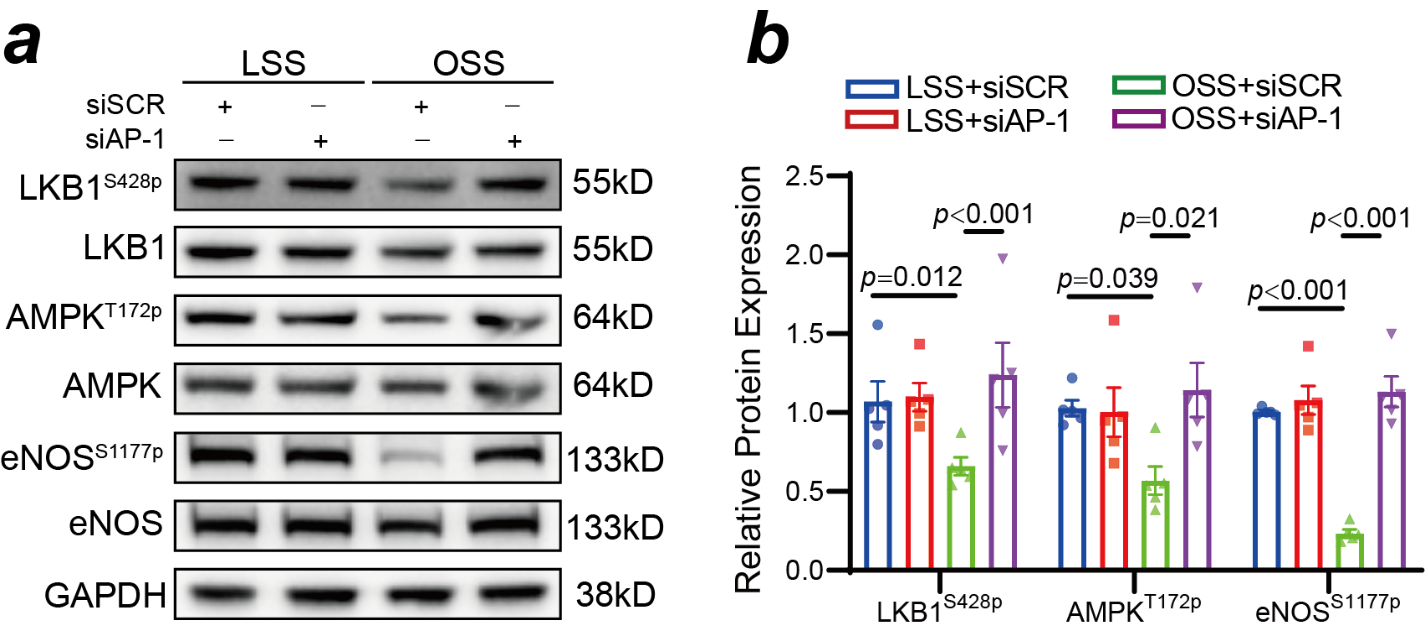


**Figure S15. Effects of AP-1 knockdown on energy metabolism in endothelial cells subject to OSS.** (a) To explore effects of AP-1 knockdown on energy metabolism in endothelial cells, AP-1 siRNA treated-HUVECs and scramble siRNA treated-HUVECs were exposure to different shear stress, LKB1^S428p^, LKB1, AMPK^T172p^, AMPK, eNOS^S1177p^, and eNOS protein expression levels were detected by Western blot. (b) Quantification of LKB1^S428p^, AMPK^T172p^, eNOS^S1177p^ protein expression levels (n=5, one-way ANOVA).


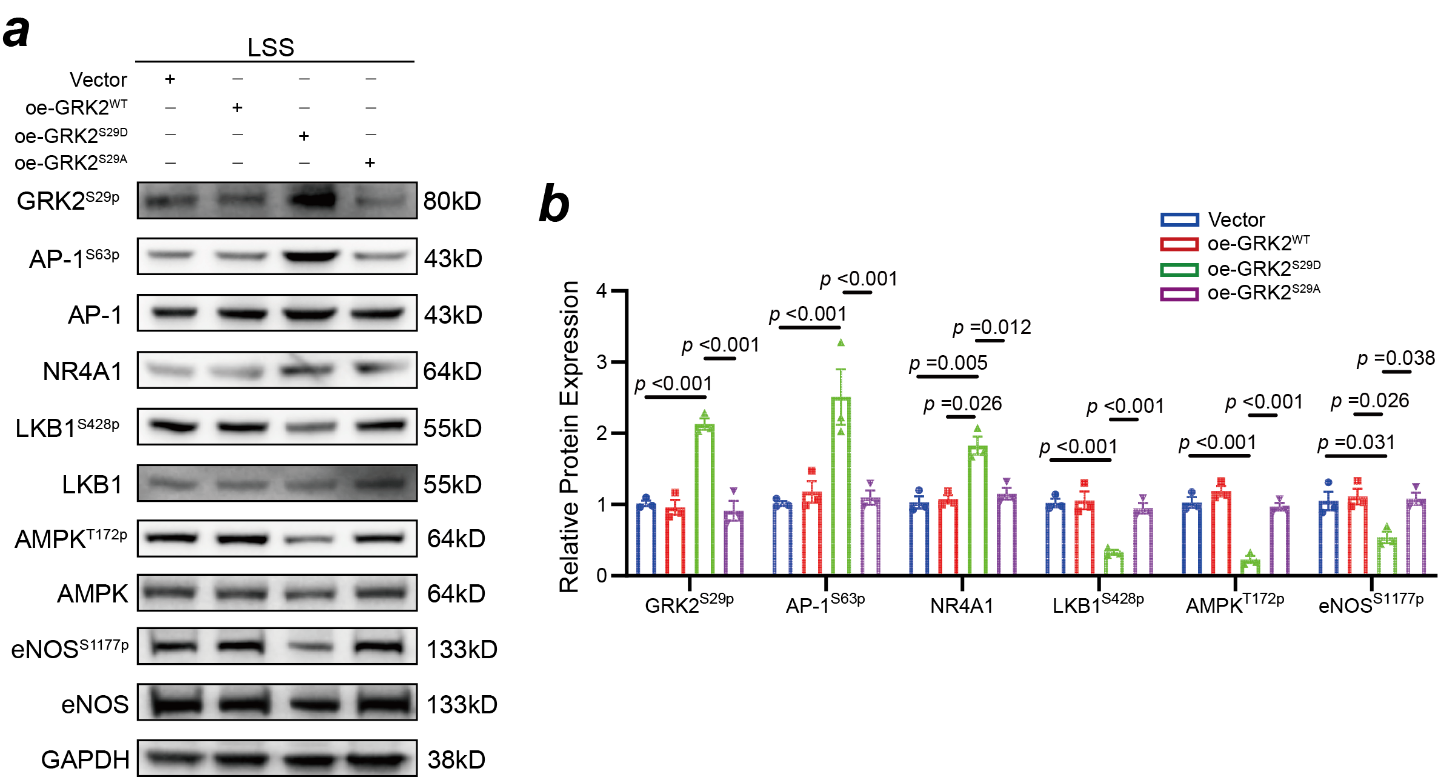


**Figure S16. Effects of overexpression of GRK2^S29D^ on AP-1^S63p^, NR4A1, LKB1^S428p^, and AMPK^T172p^ levels in HUVECs under LSS environment.** (a) HUVECs were transfected with plasmids encoding GRK2^WT^, GRK2^S29D^, or GRK2^S29A^. After LSS stimulation, protein expression levels of AP-1^S63p^, NR4A1, LKB1^S428p^, and AMPK^T172p^ levels were assessed by Western blotting. (b) Quantification of GRK2^S29p^, AP-1^S63p^, NR4A1, LKB1^S428p^, and AMPK^T172p^ levels (n=3, one-way ANOVA).

**Table S1. Sequence information of siRNAs used in RNA knock down assay.**

| **Gene Name** | **Primer Sequence** |
| --- | --- |
| Scramble | Sense: 5’-UUCUCCGAACGUGUCACGUTT-3’  Antisense:5’-ACGUGACACGUUCGGAGAATT-3’ |
| GRK2 | Sense: 5’- UGUCCAGUAACUUGAUUCCTT-3’  Antisense: 5’-GGAAUCAAGUUACUGGACATT-3’ |
| AP-1 | Sense: 5’-GUGCGCUCUUAGAGAAACUTT-3’  Antisense:5’-AGUUUCUCUAAGAGCGCACTT-3’ |
| NR4A1 | Sense: 5’-CCUGUAUCCAAGCCCAAUATT-3’  Antisense:5’-UAUUGGGCUUGGAUACAGGTT-3’ |

**Table S2. Primer sequences for reverse transcript quantitative PCR (RT-qPCR)**

| **Gene Name -** |  |
| --- | --- |
| **Human** |  |
| NR4A1 | Forward: 5’-ATGCCCTGTATCCAAGCCC-3’  Reverse:5’-GTGTAGCCGTCCATGAAGGT-3’ |
| 18S | Forward: 5’-CATTCGAACGTCTGCCCTATC-3’  Reverse:5’-CCTGCTGCCTTCCTTGGA-3’ |
| **Mus musculus** |  |
| GRK2-flox | Forward: 5’-TGAGGCTCAGGGATACCTGTCAT-3’  Reverse: 5’-CAGGCATTCCTGCTGGACTAG-3’ |
| Cdh5-CreERT2 | Forward: 5’-CACTGGGTCCTGATGGTGCCTATC-3’  Reverse: 5’-TCCTGTTGTTCAGCTTGCACCAG-3’ |

**Table S3. Primer sequences for qPCR of chromatin immunoprecipitation (ChIP) assays**

| **Promoter** | **Primer Sequence** |
| --- | --- |
| NR4A1 | Forward: 5’-GGGCGCGAGGAGCCTATTTATAGATCA-3’  Reverse:5’-ACCCTCCTCTTAAGCGCTCCGTGA-3’ |

**Table S4. Nucleic acid and amino acid sequences of GRK2^S29D^, GRK2^S29A^ and GRK2^K220R^.**

**GRK2^S29D^ nucleic acid sequence:** ATGGCGGACCTGGAGGCGGTGCTGGCCGACGTGAGCTACCTGATGGCCATGGAGAAGAGCAAGGCCACGCCGGCCGCGCGCGCCGACAAGAAGATCCTGCTGCCCGAGCCCAGCATCCGCAGTGTCATGCAGAAGTACCTGGAGGACCGGGGCGAGGTGACCTTTGAGAAGATCTTTTCCCAGAAGCTGGGGTACCTGCTCTTCCGAGACTTCTGCCTGAACCACCTGGAGGAGGCCAGGCCCTTGGTGGAATTCTATGAGGAGATCAAGAAGTACGAGAAGCTGGAGACGGAGGAGGAGCGTGTGGCCCGCAGCCGGGAGATCTTCGACTCATACATCATGAAGGAGCTGCTGGCCTGCTCGCATCCCTTCTCGAAGAGTGCCACTGAGCATGTCCAAGGCCACCTGGGGAAGAAGCAGGTGCCTCCGGATCTCTTCCAGCCATACATCGAAGAGATTTGTCAAAACCTCCGAGGGGACGTGTTCCAGAAATTCATTGAGAGCGATAAGTTCACACGGTTTTGCCAGTGGAAGAATGTGGAGCTCAACATCCACCTGACCATGAATGACTTCAGCGTGCATCGCATCATTGGGCGCGGGGGCTTTGGCGAGGTCTATGGGTGCCGGAAGGCTGACACAGGCAAGATGTACGCCATGAAGTGCCTGGACAAAAAGCGCATCAAGATGAAGCAGGGGGAGACCCTGGCCCTGAACGAGCGCATCATGCTCTCGCTCGTCAGCACTGGGGACTGCCCATTCATTGTCTGCATGTCATACGCGTTCCACACGCCAGACAAGCTCAGCTTCATCCTGGACCTCATGAACGGTGGGGACCTGCACTACCACCTCTCCCAGCACGGGGTCTTCTCAGAGGCTGACATGCGCTTCTATGCGGCCGAGATCATCCTGGGCCTGGAGCACATGCACAACCGCTTCGTGGTCTACCGGGACCTGAAGCCAGCCAACATCCTTCTGGACGAGCATGGCCACGTGCGGATCTCGGACCTGGGCCTGGCCTGTGACTTCTCCAAGAAGAAGCCCCATGCCAGCGTGGGCACCCACGGGTACATGGCTCCGGAGGTCCTGCAGAAGGGCGTGGCCTACGACAGCAGTGCCGACTGGTTCTCTCTGGGGTGCATGCTCTTCAAGTTGCTGCGGGGGCACAGCCCCTTCCGGCAGCACAAGACCAAAGACAAGCATGAGATCGACCGCATGACGCTGACGATGGCCGTGGAGCTGCCCGACTCCTTCTCCCCTGAACTACGCTCCCTGCTGGAGGGGTTGCTGCAGAGGGATGTCAACCGGAGATTGGGCTGCCTGGGCCGAGGGGCTCAGGAGGTGAAAGAGAGCCCCTTTTTCCGCTCCCTGGACTGGCAGATGGTCTTCTTGCAGAAGTACCCTCCCCCGCTGATCCCCCCACGAGGGGAGGTGAACGCGGCCGACGCCTTCGACATTGGCTCCTTCGATGAGGAGGACACAAAAGGAATCAAGTTACTGGACAGTGATCAGGAGCTCTACCGCAACTTCCCCCTCACCATCTCGGAGCGGTGGCAGCAGGAGGTGGCAGAGACTGTCTTCGACACCATCAACGCTGAGACAGACCGGCTGGAGGCTCGCAAGAAAGCCAAGAACAAGCAGCTGGGCCATGAGGAAGACTACGCCCTGGGCAAGGACTGCATCATGCATGGCTACATGTCCAAGATGGGCAACCCCTTCCTGACCCAGTGGCAGCGGCGGTACTTCTACCTGTTCCCCAACCGCCTCGAGTGGCGGGGCGAGGGCGAGGCCCCGCAGAGCCTGCTGACCATGGAGGAGATCCAGTCGGTGGAGGAGACGCAGATCAAGGAGCGCAAGTGCCTGCTCCTCAAGATCCGCGGTGGGAAACAGTTCATTTTGCAGTGCGATAGCGACCCTGAGCTGGTGCAGTGGAAGAAGGAGCTGCGCGACGCCTACCGCGAGGCCCAGCAGCTGGTGCAGCGGGTGCCCAAGATGAAGAACAAGCCGCGCTCGCCCGTGGTGGAGCTGAGCAAGGTGCCGCTGGTCCAGCGCGGCAGTGCCAACGGCCTCTGA

**GRK2^S29D^ Amino acid sequence:** MADLEAVLADVSYLMAMEKSKATPAARADKKILLPEPSIRSVMQKYLEDRGEVTFEKIFSQKLGYLLFRDFCLNHLEEARPLVEFYEEIKKYEKLETEEERVARSREIFDSYIMKELLACSHPFSKSATEHVQGHLGKKQVPPDLFQPYIEEICQNLRGDVFQKFIESDKFTRFCQWKNVELNIHLTMNDFSVHRIIGRGGFGEVYGCRKADTGKMYAMKCLDKKRIKMKQGETLALNERIMLSLVSTGDCPFIVCMSYAFHTPDKLSFILDLMNGGDLHYHLSQHGVFSEADMRFYAAEIILGLEHMHNRFVVYRDLKPANILLDEHGHVRISDLGLACDFSKKKPHASVGTHGYMAPEVLQKGVAYDSSADWFSLGCMLFKLLRGHSPFRQHKTKDKHEIDRMTLTMAVELPDSFSPELRSLLEGLLQRDVNRRLGCLGRGAQEVKESPFFRSLDWQMVFLQKYPPPLIPPRGEVNAADAFDIGSFDEEDTKGIKLLDSDQELYRNFPLTISERWQQEVAETVFDTINAETDRLEARKKAKNKQLGHEEDYALGKDCIMHGYMSKMGNPFLTQWQRRYFYLFPNRLEWRGEGEAPQSLLTMEEIQSVEETQIKERKCLLLKIRGGKQFILQCDSDPELVQWKKELRDAYREAQQLVQRVPKMKNKPRSPVVELSKVPLVQRGSANGL

**GRK2^S29A^ nucleic acid sequence:**

ATGGCGGACCTGGAGGCGGTGCTGGCCGACGTGAGCTACCTGATGGCCATGGAGAAGAGCAAGGCCACGCCGGCCGCGCGCGCCGCCAAGAAGATCCTGCTGCCCGAGCCCAGCATCCGCAGTGTCATGCAGAAGTACCTGGAGGACCGGGGCGAGGTGACCTTTGAGAAGATCTTTTCCCAGAAGCTGGGGTACCTGCTCTTCCGAGACTTCTGCCTGAACCACCTGGAGGAGGCCAGGCCCTTGGTGGAATTCTATGAGGAGATCAAGAAGTACGAGAAGCTGGAGACGGAGGAGGAGCGTGTGGCCCGCAGCCGGGAGATCTTCGACTCATACATCATGAAGGAGCTGCTGGCCTGCTCGCATCCCTTCTCGAAGAGTGCCACTGAGCATGTCCAAGGCCACCTGGGGAAGAAGCAGGTGCCTCCGGATCTCTTCCAGCCATACATCGAAGAGATTTGTCAAAACCTCCGAGGGGACGTGTTCCAGAAATTCATTGAGAGCGATAAGTTCACACGGTTTTGCCAGTGGAAGAATGTGGAGCTCAACATCCACCTGACCATGAATGACTTCAGCGTGCATCGCATCATTGGGCGCGGGGGCTTTGGCGAGGTCTATGGGTGCCGGAAGGCTGACACAGGCAAGATGTACGCCATGAAGTGCCTGGACAAAAAGCGCATCAAGATGAAGCAGGGGGAGACCCTGGCCCTGAACGAGCGCATCATGCTCTCGCTCGTCAGCACTGGGGACTGCCCATTCATTGTCTGCATGTCATACGCGTTCCACACGCCAGACAAGCTCAGCTTCATCCTGGACCTCATGAACGGTGGGGACCTGCACTACCACCTCTCCCAGCACGGGGTCTTCTCAGAGGCTGACATGCGCTTCTATGCGGCCGAGATCATCCTGGGCCTGGAGCACATGCACAACCGCTTCGTGGTCTACCGGGACCTGAAGCCAGCCAACATCCTTCTGGACGAGCATGGCCACGTGCGGATCTCGGACCTGGGCCTGGCCTGTGACTTCTCCAAGAAGAAGCCCCATGCCAGCGTGGGCACCCACGGGTACATGGCTCCGGAGGTCCTGCAGAAGGGCGTGGCCTACGACAGCAGTGCCGACTGGTTCTCTCTGGGGTGCATGCTCTTCAAGTTGCTGCGGGGGCACAGCCCCTTCCGGCAGCACAAGACCAAAGACAAGCATGAGATCGACCGCATGACGCTGACGATGGCCGTGGAGCTGCCCGACTCCTTCTCCCCTGAACTACGCTCCCTGCTGGAGGGGTTGCTGCAGAGGGATGTCAACCGGAGATTGGGCTGCCTGGGCCGAGGGGCTCAGGAGGTGAAAGAGAGCCCCTTTTTCCGCTCCCTGGACTGGCAGATGGTCTTCTTGCAGAAGTACCCTCCCCCGCTGATCCCCCCACGAGGGGAGGTGAACGCGGCCGACGCCTTCGACATTGGCTCCTTCGATGAGGAGGACACAAAAGGAATCAAGTTACTGGACAGTGATCAGGAGCTCTACCGCAACTTCCCCCTCACCATCTCGGAGCGGTGGCAGCAGGAGGTGGCAGAGACTGTCTTCGACACCATCAACGCTGAGACAGACCGGCTGGAGGCTCGCAAGAAAGCCAAGAACAAGCAGCTGGGCCATGAGGAAGACTACGCCCTGGGCAAGGACTGCATCATGCATGGCTACATGTCCAAGATGGGCAACCCCTTCCTGACCCAGTGGCAGCGGCGGTACTTCTACCTGTTCCCCAACCGCCTCGAGTGGCGGGGCGAGGGCGAGGCCCCGCAGAGCCTGCTGACCATGGAGGAGATCCAGTCGGTGGAGGAGACGCAGATCAAGGAGCGCAAGTGCCTGCTCCTCAAGATCCGCGGTGGGAAACAGTTCATTTTGCAGTGCGATAGCGACCCTGAGCTGGTGCAGTGGAAGAAGGAGCTGCGCGACGCCTACCGCGAGGCCCAGCAGCTGGTGCAGCGGGTGCCCAAGATGAAGAACAAGCCGCGCTCGCCCGTGGTGGAGCTGAGCAAGGTGCCGCTGGTCCAGCGCGGCAGTGCCAACGGCCTCTGA

**GRK2^S29A^ amino acid sequence:**

MADLEAVLADVSYLMAMEKSKATPAARAAKKILLPEPSIRSVMQKYLEDRGEVTFEKIFSQKLGYLLFRDFCLNHLEEARPLVEFYEEIKKYEKLETEEERVARSREIFDSYIMKELLACSHPFSKSATEHVQGHLGKKQVPPDLFQPYIEEICQNLRGDVFQKFIESDKFTRFCQWKNVELNIHLTMNDFSVHRIIGRGGFGEVYGCRKADTGKMYAMKCLDKKRIKMKQGETLALNERIMLSLVSTGDCPFIVCMSYAFHTPDKLSFILDLMNGGDLHYHLSQHGVFSEADMRFYAAEIILGLEHMHNRFVVYRDLKPANILLDEHGHVRISDLGLACDFSKKKPHASVGTHGYMAPEVLQKGVAYDSSADWFSLGCMLFKLLRGHSPFRQHKTKDKHEIDRMTLTMAVELPDSFSPELRSLLEGLLQRDVNRRLGCLGRGAQEVKESPFFRSLDWQMVFLQKYPPPLIPPRGEVNAADAFDIGSFDEEDTKGIKLLDSDQELYRNFPLTISERWQQEVAETVFDTINAETDRLEARKKAKNKQLGHEEDYALGKDCIMHGYMSKMGNPFLTQWQRRYFYLFPNRLEWRGEGEAPQSLLTMEEIQSVEETQIKERKCLLLKIRGGKQFILQCDSDPELVQWKKELRDAYREAQQLVQRVPKMKNKPRSPVVELSKVPLVQRGSANGL

**GRK2^K220R^ nucleic acid sequence:** ATGGCGGACCTGGAGGCGGTGCTGGCCGACGTGAGCTACCTGATGGCCATGGAGAAGAGCAAGGCCACGCCGGCCGCGCGCGCCAGCAAGAAGATCCTGCTGCCCGAGCCCAGCATCCGCAGTGTCATGCAGAAGTACCTGGAGGACCGGGGCGAGGTGACCTTTGAGAAGATCTTTTCCCAGAAGCTGGGGTACCTGCTCTTCCGAGACTTCTGCCTGAACCACCTGGAGGAGGCCAGGCCCTTGGTGGAATTCTATGAGGAGATCAAGAAGTACGAGAAGCTGGAGACGGAGGAGGAGCGTGTGGCCCGCAGCCGGGAGATCTTCGACTCATACATCATGAAGGAGCTGCTGGCCTGCTCGCATCCCTTCTCGAAGAGTGCCACTGAGCATGTCCAAGGCCACCTGGGGAAGAAGCAGGTGCCTCCGGATCTCTTCCAGCCATACATCGAAGAGATTTGTCAAAACCTCCGAGGGGACGTGTTCCAGAAATTCATTGAGAGCGATAAGTTCACACGGTTTTGCCAGTGGAAGAATGTGGAGCTCAACATCCACCTGACCATGAATGACTTCAGCGTGCATCGCATCATTGGGCGCGGGGGCTTTGGCGAGGTCTATGGGTGCCGGAAGGCTGACACAGGCAAGATGTACGCCATGAGGTGCCTGGACAAAAAGCGCATCAAGATGAAGCAGGGGGAGACCCTGGCCCTGAACGAGCGCATCATGCTCTCGCTCGTCAGCACTGGGGACTGCCCATTCATTGTCTGCATGTCATACGCGTTCCACACGCCAGACAAGCTCAGCTTCATCCTGGACCTCATGAACGGTGGGGACCTGCACTACCACCTCTCCCAGCACGGGGTCTTCTCAGAGGCTGACATGCGCTTCTATGCGGCCGAGATCATCCTGGGCCTGGAGCACATGCACAACCGCTTCGTGGTCTACCGGGACCTGAAGCCAGCCAACATCCTTCTGGACGAGCATGGCCACGTGCGGATCTCGGACCTGGGCCTGGCCTGTGACTTCTCCAAGAAGAAGCCCCATGCCAGCGTGGGCACCCACGGGTACATGGCTCCGGAGGTCCTGCAGAAGGGCGTGGCCTACGACAGCAGTGCCGACTGGTTCTCTCTGGGGTGCATGCTCTTCAAGTTGCTGCGGGGGCACAGCCCCTTCCGGCAGCACAAGACCAAAGACAAGCATGAGATCGACCGCATGACGCTGACGATGGCCGTGGAGCTGCCCGACTCCTTCTCCCCTGAACTACGCTCCCTGCTGGAGGGGTTGCTGCAGAGGGATGTCAACCGGAGATTGGGCTGCCTGGGCCGAGGGGCTCAGGAGGTGAAAGAGAGCCCCTTTTTCCGCTCCCTGGACTGGCAGATGGTCTTCTTGCAGAAGTACCCTCCCCCGCTGATCCCCCCACGAGGGGAGGTGAACGCGGCCGACGCCTTCGACATTGGCTCCTTCGATGAGGAGGACACAAAAGGAATCAAGTTACTGGACAGTGATCAGGAGCTCTACCGCAACTTCCCCCTCACCATCTCGGAGCGGTGGCAGCAGGAGGTGGCAGAGACTGTCTTCGACACCATCAACGCTGAGACAGACCGGCTGGAGGCTCGCAAGAAAGCCAAGAACAAGCAGCTGGGCCATGAGGAAGACTACGCCCTGGGCAAGGACTGCATCATGCATGGCTACATGTCCAAGATGGGCAACCCCTTCCTGACCCAGTGGCAGCGGCGGTACTTCTACCTGTTCCCCAACCGCCTCGAGTGGCGGGGCGAGGGCGAGGCCCCGCAGAGCCTGCTGACCATGGAGGAGATCCAGTCGGTGGAGGAGACGCAGATCAAGGAGCGCAAGTGCCTGCTCCTCAAGATCCGCGGTGGGAAACAGTTCATTTTGCAGTGCGATAGCGACCCTGAGCTGGTGCAGTGGAAGAAGGAGCTGCGCGACGCCTACCGCGAGGCCCAGCAGCTGGTGCAGCGGGTGCCCAAGATGAAGAACAAGCCGCGCTCGCCCGTGGTGGAGCTGAGCAAGGTGCCGCTGGTCCAGCGCGGCAGTGCCAACGGCCTCTGA

**GRK2^K220R^ amino acid sequence:** MADLEAVLADVSYLMAMEKSKATPAARASKKILLPEPSIRSVMQKYLEDRGEVTFEKIFSQKLGYLLFRDFCLNHLEEARPLVEFYEEIKKYEKLETEEERVARSREIFDSYIMKELLACSHPFSKSATEHVQGHLGKKQVPPDLFQPYIEEICQNLRGDVFQKFIESDKFTRFCQWKNVELNIHLTMNDFSVHRIIGRGGFGEVYGCRKADTGKMYAMRCLDKKRIKMKQGETLALNERIMLSLVSTGDCPFIVCMSYAFHTPDKLSFILDLMNGGDLHYHLSQHGVFSEADMRFYAAEIILGLEHMHNRFVVYRDLKPANILLDEHGHVRISDLGLACDFSKKKPHASVGTHGYMAPEVLQKGVAYDSSADWFSLGCMLFKLLRGHSPFRQHKTKDKHEIDRMTLTMAVELPDSFSPELRSLLEGLLQRDVNRRLGCLGRGAQEVKESPFFRSLDWQMVFLQKYPPPLIPPRGEVNAADAFDIGSFDEEDTKGIKLLDSDQELYRNFPLTISERWQQEVAETVFDTINAETDRLEARKKAKNKQLGHEEDYALGKDCIMHGYMSKMGNPFLTQWQRRYFYLFPNRLEWRGEGEAPQSLLTMEEIQSVEETQIKERKCLLLKIRGGKQFILQCDSDPELVQWKKELRDAYREAQQLVQRVPKMKNKPRSPVVELSKVPLVQRGSANGL

**Table S5. Nucleic acid and amino acid sequences of AP-1^S63D^ and AP-1^S63A^.**

**AP-1^S63D^ nucleic acid sequence:** ATGACTGCAAAGATGGAAACGACCTTCTATGACGATGCCCTCAACGCCTCGTTCCTCCCGTCCGAGAGCGGACCTTATGGCTACAGTAACCCCAAGATCCTGAAACAGAGCATGACCCTGAACCTGGCCGACCCAGTGGGGAGCCTGAAGCCGCACCTCCGCGCCAAGAACTCGGACCTCCTCACCGACCCCGACGTGGGGCTGCTCAAGCTGGCGTCGCCCGAGCTGGAGCGCCTGATAATCCAGTCCAGCAACGGGCACATCACCACCACGCCGACCCCCACCCAGTTCCTGTGCCCCAAGAACGTGACAGATGAGCAGGAGGGCTTCGCCGAGGGCTTCGTGCGCGCCCTGGCCGAACTGCACAGCCAGAACACGCTGCCCAGCGTCACGTCGGCGGCGCAGCCGGTCAACGGGGCAGGCATGGTGGCTCCCGCGGTAGCCTCGGTGGCAGGGGGCAGCGGCAGCGGCGGCTTCAGCGCCAGCCTGCACAGCGAGCCGCCGGTCTACGCAAACCTCAGCAACTTCAACCCAGGCGCGCTGAGCAGCGGCGGCGGGGCGCCCTCCTACGGCGCGGCCGGCCTGGCCTTTCCCGCGCAACCCCAGCAGCAGCAGCAGCCGCCGCACCACCTGCCCCAGCAGATGCCCGTGCAGCACCCGCGGCTGCAGGCCCTGAAGGAGGAGCCTCAGACAGTGCCCGAGATGCCCGGCGAGACACCGCCCCTGTCCCCCATCGACATGGAGTCCCAGGAGCGGATCAAGGCGGAGAGGAAGCGCATGAGGAACCGCATCGCTGCCTCCAAGTGCCGAAAAAGGAAGCTGGAGAGAATCGCCCGGCTGGAGGAAAAAGTGAAAACCTTGAAAGCTCAGAACTCGGAGCTGGCGTCCACGGCCAACATGCTCAGGGAACAGGTGGCACAGCTTAAACAGAAAGTCATGAACCACGTTAACAGTGGGTGCCAACTCATGCTAACGCAGCAGTTGCAAACATTTTGA

**AP-1^S63D^ amino acid sequence:** MTAKMETTFYDDALNASFLPSESGPYGYSNPKILKQSMTLNLADPVGSLKPHLRAKNSDLLTDPDVGLLKLASPELERLIIQSSNGHITTTPTPTQFLCPKNVTDEQEGFAEGFVRALAELHSQNTLPSVTSAAQPVNGAGMVAPAVASVAGGSGSGGFSASLHSEPPVYANLSNFNPGALSSGGGAPSYGAAGLAFPAQPQQQQQPPHHLPQQMPVQHPRLQALKEEPQTVPEMPGETPPLSPIDMESQERIKAERKRMRNRIAASKCRKRKLERIARLEEKVKTLKAQNSELASTANMLREQVAQLKQKVMNHVNSGCQLMLTQQLQTF

**AP-1^S63A^ nucleic acid sequence:** ATGACTGCAAAGATGGAAACGACCTTCTATGACGATGCCCTCAACGCCTCGTTCCTCCCGTCCGAGAGCGGACCTTATGGCTACAGTAACCCCAAGATCCTGAAACAGAGCATGACCCTGAACCTGGCCGACCCAGTGGGGAGCCTGAAGCCGCACCTCCGCGCCAAGAACTCGGACCTCCTCACCGCGCCCGACGTGGGGCTGCTCAAGCTGGCGTCGCCCGAGCTGGAGCGCCTGATAATCCAGTCCAGCAACGGGCACATCACCACCACGCCGACCCCCACCCAGTTCCTGTGCCCCAAGAACGTGACAGATGAGCAGGAGGGCTTCGCCGAGGGCTTCGTGCGCGCCCTGGCCGAACTGCACAGCCAGAACACGCTGCCCAGCGTCACGTCGGCGGCGCAGCCGGTCAACGGGGCAGGCATGGTGGCTCCCGCGGTAGCCTCGGTGGCAGGGGGCAGCGGCAGCGGCGGCTTCAGCGCCAGCCTGCACAGCGAGCCGCCGGTCTACGCAAACCTCAGCAACTTCAACCCAGGCGCGCTGAGCAGCGGCGGCGGGGCGCCCTCCTACGGCGCGGCCGGCCTGGCCTTTCCCGCGCAACCCCAGCAGCAGCAGCAGCCGCCGCACCACCTGCCCCAGCAGATGCCCGTGCAGCACCCGCGGCTGCAGGCCCTGAAGGAGGAGCCTCAGACAGTGCCCGAGATGCCCGGCGAGACACCGCCCCTGTCCCCCATCGACATGGAGTCCCAGGAGCGGATCAAGGCGGAGAGGAAGCGCATGAGGAACCGCATCGCTGCCTCCAAGTGCCGAAAAAGGAAGCTGGAGAGAATCGCCCGGCTGGAGGAAAAAGTGAAAACCTTGAAAGCTCAGAACTCGGAGCTGGCGTCCACGGCCAACATGCTCAGGGAACAGGTGGCACAGCTTAAACAGAAAGTCATGAACCACGTTAACAGTGGGTGCCAACTCATGCTAACGCAGCAGTTGCAAACATTTTGA

**AP-1^S63A^ amino acid sequence:** MTAKMETTFYDDALNASFLPSESGPYGYSNPKILKQSMTLNLADPVGSLKPHLRAKNSDLLTAPDVGLLKLASPELERLIIQSSNGHITTTPTPTQFLCPKNVTDEQEGFAEGFVRALAELHSQNTLPSVTSAAQPVNGAGMVAPAVASVAGGSGSGGFSASLHSEPPVYANLSNFNPGALSSGGGAPSYGAAGLAFPAQPQQQQQPPHHLPQQMPVQHPRLQALKEEPQTVPEMPGETPPLSPIDMESQERIKAERKRMRNRIAASKCRKRKLERIARLEEKVKTLKAQNSELASTANMLREQVAQLKQKVMNHVNSGCQLMLTQQLQTF

**Table S6. Nucleic acid and amino acid sequences of NR4A1.**

**NR4A1 nucleic acid sequence:**

ATGCCCTGTATCCAAGCCCAATATGGGACACCAGCACCGAGTCCGGGACCCCGTGACCACCTGGCAAGCGACCCCCTGACCCCTGAGTTCATCAAGCCCACCATGGACCTGGCCAGCCCCGAGGCAGCCCCCGCTGCCCCCACTGCCCTGCCCAGCTTCAGCACCTTCATGGACGGCTACACAGGAGAGTTTGACACCTTCCTCTACCAGCTGCCAGGAACAGTCCAGCCATGCTCCTCAGCCTCCTCCTCGGCCTCCTCCACATCCTCGTCCTCAGCCACCTCCCCTGCCTCTGCCTCCTTCAAGTTCGAGGACTTCCAGGTGTACGGCTGCTACCCCGGCCCCCTGAGCGGCCCAGTGGATGAGGCCCTGTCCTCCAGTGGCTCTGACTACTATGGCAGCCCCTGCTCGGCCCCGTCGCCCTCCACGCCCAGCTTCCAGCCGCCCCAGCTCTCTCCCTGGGATGGCTCCTTCGGCCACTTCTCGCCCAGCCAGACTTACGAAGGCCTGCGGGCATGGACAGAGCAGCTGCCCAAAGCCTCTGGGCCCCCACAGCCTCCAGCCTTCTTTTCCTTCAGTCCTCCCACCGGCCCCAGCCCCAGCCTGGCCCAGAGCCCCCTGAAGTTGTTCCCCTCACAGGCCACCCACCAGCTGGGGGAGGGAGAGAGCTATTCCATGCCTACGGCCTTCCCAGGTTTGGCACCCACTTCTCCACACCTTGAGGGCTCGGGGATACTGGATACACCCGTGACCTCAACCAAGGCCCGGAGCGGGGCCCCAGGTGGAAGTGAAGGCCGCTGTGCTGTGTGTGGGGACAACGCTTCATGCCAGCATTATGGTGTCCGCACATGTGAGGGCTGCAAGGGCTTCTTCAAGCGCACAGTGCAGAAAAACGCCAAGTACATCTGCCTGGCTAACAAGGACTGCCCTGTGGACAAGAGGCGGCGAAACCGCTGCCAGTTCTGCCGCTTCCAGAAGTGCCTGGCGGTGGGCATGGTGAAGGAAGTTGTCCGAACAGACAGCCTGAAGGGGCGGCGGGGCCGGCTACCTTCAAAACCCAAGCAGCCCCCAGATGCCTCCCCTGCCAATCTCCTCACTTCCCTGGTCCGTGCACACCTGGACTCAGGGCCCAGCACTGCCAAACTGGACTACTCCAAGTTCCAGGAGCTGGTGCTGCCCCACTTTGGGAAGGAAGATGCTGGGGATGTACAGCAGTTCTACGACCTGCTCTCCGGTTCTCTGGAGGTCATCCGCAAGTGGGCGGAGAAGATCCCTGGCTTTGCTGAGCTGTCACCGGCTGACCAGGACCTGTTGCTGGAGTCGGCCTTCCTGGAGCTCTTCATCCTCCGCCTGGCGTACAGGTCTAAGCCAGGCGAGGGCAAGCTCATCTTCTGCTCAGGCCTGGTGCTACACCGGCTGCAGTGTGCCCGTGGCTTCGGGGACTGGATTGACAGTATCCTGGCCTTCTCAAGGTCCCTGCACAGCTTGCTTGTCGATGTCCCTGCCTTCGCCTGCCTCTCTGCCCTTGTCCTCATCACCGACCGGCATGGGCTGCAGGAGCCGCGGCGGGTGGAGGAGCTGCAGAACCGCATCGCCAGCTGCCTGAAGGAGCACGTGGCAGCTGTGGCGGGCGAGCCCCAGCCAGCCAGCTGCCTGTCACGTCTGTTGGGCAAACTGCCCGAGCTGCGGACCCTGTGCACCCAGGGCCTGCAGCGCATCTTCTACCTCAAGCTGGAGGACTTGGTGCCCCCTCCACCCATCATTGACAAGATCTTCATGGACACGCTGCCCTTCGGAGGTGGAGGATCAGAACAAAAACTCATCTCAGAAGAGGATCTGTGA

**NR4A1 amino acid sequence:**

MPCIQAQYGTPAPSPGPRDHLASDPLTPEFIKPTMDLASPEAAPAAPTALPSFSTFMDGYTGEFDTFLYQLPGTVQPCSSASSSASSTSSSSATSPASASFKFEDFQVYGCYPGPLSGPVDEALSSSGSDYYGSPCSAPSPSTPSFQPPQLSPWDGSFGHFSPSQTYEGLRAWTEQLPKASGPPQPPAFFSFSPPTGPSPSLAQSPLKLFPSQATHQLGEGESYSMPTAFPGLAPTSPHLEGSGILDTPVTSTKARSGAPGGSEGRCAVCGDNASCQHYGVRTCEGCKGFFKRTVQKNAKYICLANKDCPVDKRRRNRCQFCRFQKCLAVGMVKEVVRTDSLKGRRGRLPSKPKQPPDASPANLLTSLVRAHLDSGPSTAKLDYSKFQELVLPHFGKEDAGDVQQFYDLLSGSLEVIRKWAEKIPGFAELSPADQDLLLESAFLELFILRLAYRSKPGEGKLIFCSGLVLHRLQCARGFGDWIDSILAFSRSLHSLLVDVPAFACLSALVLITDRHGLQEPRRVEELQNRIASCLKEHVAAVAGEPQPASCLSRLLGKLPELRTLCTQGLQRIFYLKLEDLVPPPPIIDKIFMDTLPFGGGGSEQKLISEEDL*

**Table S7. Nucleic acid and amino acid sequences of LKB1^S428D^.**

**LKB1^S428D^ nucleic acid sequence:** ATGGAGGTGGTGGACCCGCAGCAGCTGGGCATGTTCACGGAGGGCGAGCTGATGTCGGTGGGTATGGACACGTTCATCCACCGCATCGACTCCACCGAGGTCATCTACCAGCCGCGCCGCAAGCGGGCCAAGCTCATCGGCAAGTACCTGATGGGGGACCTGCTGGGGGAAGGCTCTTACGGCAAGGTGAAGGAGGTGCTGGACTCGGAGACGCTGTGCAGGAGGGCCGTCAAGATCCTCAAGAAGAAGAAGTTGCGAAGGATCCCCAACGGGGAGGCCAACGTGAAGAAGGAAATTCAACTACTGAGGAGGTTACGGCACAAAAATGTCATCCAGCTGGTGGATGTGTTATACAACGAAGAGAAGCAGAAAATGTATATGGTGATGGAGTACTGCGTGTGTGGCATGCAGGAAATGCTGGACAGCGTGCCGGAGAAGCGTTTCCCAGTGTGCCAGGCCCACGGGTACTTCTGTCAGCTGATTGACGGCCTGGAGTACCTGCATAGCCAGGGCATTGTGCACAAGGACATCAAGCCGGGGAACCTGCTGCTCACCACCGGTGGCACCCTCAAAATCTCCGACCTGGGCGTGGCCGAGGCACTGCACCCGTTCGCGGCGGACGACACCTGCCGGACCAGCCAGGGCTCCCCGGCTTTCCAGCCGCCCGAGATTGCCAACGGCCTGGACACCTTCTCCGGCTTCAAGGTGGACATCTGGTCGGCTGGGGTCACCCTCTACAACATCACCACGGGTCTGTACCCCTTCGAAGGGGACAACATCTACAAGTTGTTTGAGAACATCGGGAAGGGGAGCTACGCCATCCCGGGCGACTGTGGCCCCCCGCTCTCTGACCTGCTGAAAGGGATGCTTGAGTACGAACCGGCCAAGAGGTTCTCCATCCGGCAGATCCGGCAGCACAGCTGGTTCCGGAAGAAACATCCTCCGGCTGAAGCACCAGTGCCCATCCCACCGAGCCCAGACACCAAGGACCGGTGGCGCAGCATGACTGTGGTGCCGTACTTGGAGGACCTGCACGGCGCGGACGAGGACGAGGACCTCTTCGACATCGAGGATGACATCATCTACACTCAGGACTTCACGGTGCCCGGACAGGTCCCAGAAGAGGAGGCCAGTCACAATGGACAGCGCCGGGGCCTCCCCAAGGCCGTGTGTATGAACGGCACAGAGGCGGCGCAGCTGAGCACCAAATCCAGGGCGGAGGGCCGGGCCCCCAACCCTGCCCGCAAGGCCTGCTCCGCCAGCAGCAAGATCCGCCGGCTGGACGCCTGCAAGCAGCAGTGA

**LKB1^S428D^ amino acid sequence:** MEVVDPQQLGMFTEGELMSVGMDTFIHRIDSTEVIYQPRRKRAKLIGKYLMGDLLGEGSYGKVKEVLDSETLCRRAVKILKKKKLRRIPNGEANVKKEIQLLRRLRHKNVIQLVDVLYNEEKQKMYMVMEYCVCGMQEMLDSVPEKRFPVCQAHGYFCQLIDGLEYLHSQGIVHKDIKPGNLLLTTGGTLKISDLGVAEALHPFAADDTCRTSQGSPAFQPPEIANGLDTFSGFKVDIWSAGVTLYNITTGLYPFEGDNIYKLFENIGKGSYAIPGDCGPPLSDLLKGMLEYEPAKRFSIRQIRQHSWFRKKHPPAEAPVPIPPSPDTKDRWRSMTVVPYLEDLHGADEDEDLFDIEDDIIYTQDFTVPGQVPEEEASHNGQRRGLPKAVCMNGTEAAQLSTKSRAEGRAPNPARKACSASSKIRRLDACKQQ

**Table S8. Nucleic acid sequences of Truncated NR4A1 Promoter.**

**-2.3KB:**

AGGGGGTTGGCTCCAGGACCCCAACTTGTTACTGCGAATACTCAAGTCCCACGTTCGGCCCTGTGGAACCTGTAGATACTAAAAGTCGGCCCTCTGTATATGTGGGTTTTTGGATCCATGTTGGATTGAAAAAAAATCCCCAAATCCCCATATGAGTGGACCTACACAGTTCAAACGTGTTGTTCAAGGGTCAACTGTGTATGAAGACTGCTCCATCTGTGGTTGGACAGTGAGGCTTTAGAAAGAGGAGGACAGGCAGGCTTAATGGATGGTCTTCATGGGTGTGGGAAAGGAGGAGTGGTCACGTGGGCAGAGCAGTTGGTCGTGGGACCAGTGAGGCATGGGCTGTGGCTGGCAGTAGGAAAGCGGGAGAGGCTGAGTGCAGTGGCTCATGCCTATAATCCCAAGCACTTTGGGAAGCCAAGGCAAGAGGCTTGAGCCTGGGAGGTCCAGGCTGCAGTAAGCCATAATCATGCCACTGCATTCTAGGCTGGGCAACAGAACAAGACCTTGTCTCAAAGACAACAAAAACAAAAACAAAAACGGGGAGAGGAAAGGACCCAAAGATGTTCCAAAAAGAATTTCAAGGAAACAGTGAAGGTTGCAAATGGGGCATCAGGAGCCAGGAGCAGAGATTAGGTGGGACGCAGGCGGGGGTGCAGAAATGACAAGTTCTGTCTGGAACTGAGGAAATTTGAGGTGCTTGTGGGACCTTTGAGTGGGCAGAAAGCGGGGGCTGGAGACAAGGGTGTGGGAAAACGTGATGGGGGTTGAGGGTGGAATTCGGGCCGGCAGAGCCTCCGGGGCAAGAGAAAGTCCTATGGGGTGACCAGATGAGTTGAGAACTGGGTGGGTGGGTGCAGGCTCAATGGAAACAGAAACTCATGGATATTGACTTACATGGGAGAAAGGGGAATGGATGGGGGTCGCAGTGGGGTGGGAGGCTCCTCTTTTCCTTGTCTTTTGTTTTTTTGCCCTTCCCTCTTCTCTTACTTTTCCGTGAGGGCTCCCCAGGCTCAGGAGAGATCAGGGTGGAAAGGGGACGGCCAAACCAGGGAAGGCTCCAGGTGGCTGAAGCCTGGTCTGTGCCAGCAGGGCCCTGGCGGGCGTGTTCCCACTCCACCGGGCAGGTGATAACTGGTCAGAGCTGCCTCCCCACAGGTGCTGGAGGTAGGCTGGGAGGGCCGGTGCTCCCTGATGTGGACAGGGGGAGGGGTATTGATAAGAGGCGTGGAGAGATCCCTAGAGATGCAGTCTGTGGCCCCTGGGTTCCAACCCAGTGTGCCACCACCTGGCTGTGTGACCTTCAGCAAGTGCCATTATTTCTCTGAGCCTGTTTGTTTATAAAATGAGGAAGAGTTGGCACAAGTTTGAAGAATTTCTCAGGCTCCACCCGGTTCTGAAATTCCGGTAATTTCCCAACTAGGGTGCACCCCCCTGTAAGGGGCTGGGGAGGGGACGGTGCGAACCAAGTTCAACTTGTGGAGCGGAGCCAGAGCTGTTGGCCGAGCTTGGGCCTGGCCAACGCCCTGCCCTCGGGAAGGTCCTGTGTAGGGAGACTGCCTGGAGGGACTAAGCGAGGGCCTCTAACGTCTCAGGGGCAGCCTCTCAGCCTGAGACCCTGCTGGGGAAGCCGCCTCCTGTGCATAGCTGCGCCCTAGGGCTGAGGTGAGGGCGCAGGCTCCCCAGGGTGTGTCCGAATTGCCCGCCTCAGCCCGCGGCCGTCCTGACCACCCAGCAGCGGCAGCAGCGACACCCTAGGGCTCCAGGAAGGGCTTGGGAAGGTGTAAGGCAGGGGCTAGGCTCGGAGGGAGCCGGAGGGACCGGGCGCAGGTTGGCTCCCGGGAGCAACTGGAGAGTGAGGAGATCCTCATCCGGGGAAGCCCGCAGGCCGCGTCTCTACAGCGCCCTTCTCGGGTCTGGCCCTCCCGCTGGTTATTCTGGACCTGGGGGCCCCCAGCTGGGACCCGAGTCCGGTGCAGGGGAGCCTAGTGGGCCTGGGAGCTGCTATTTTTAGCGGGCGCGGCGGCGCGAGGACCTATTTATAGATCAAACAAACCGCGCTCCCTGCCTCAATGGAAACCCGCGTGCGTGACGCGCGCGCAGACATTCCAGGCCCCCCCCTCCTCGCCCCGCCCCCTCGGGCTCCCCGGGCCGCACCTCCCCCTGGCCGCCTCCCGCCGGAACCGCACCGCCCCCCGCGAACCCCTTGTATGGCCAAAGCTCGACGGGCAGGCCGTGCAGTGGCACCCCGCCCTCCCTCGGTGCATCACGGAGCGCTTAAGAGGAGGGTCGGGCTCGGCCGGGGAGTCCCACTGGCGGAGGCTACGAAACTTGGGGGAGTGCACAGAAGAACTTCGGGAGCGCACGCAGGACCAGGGACCAGGCTGAGACTCGGGGCGCCAGTCCGGGCAGGGGCAGCGGGAGCCGGCCGGAG

**-1.9KB:**

CCAGCACTTTGGGAAGCCAAGGCAAGAGGCTTGAGCCTGGGAGGTCCAGGCTGCAGTAAGCCATAATCATGCCACTGCATTCTAGGCTGGGCAACAGAACAAGACCTTGTCTCAAAGACAACAAAAACAAAACAAAAACGGGGAGAGGAAAGGACCCAAGAGATGTTCCAAAAAAGAATTTCAAGGAAACAGTGAAGGTTGCAAATGGGGCATCAGGAGCCAGGAGCAGAGATTAGGTGGGACGCAGGCGGGGGTGCAGAAATGACAAGTTCTGTCTGGAACTGAGGAAATTTGAGGTGCTTGTGGGACCTTTGAGTGGGCAGAAAGCGGGGGCTGGAGACAAGGGTGTGGGAAAACGTGATGGGGGTTGAGGGTGGAATTCGGGCCGGCAGAGCCTCCGGGGCAAGAGAAAGTCCTATGGGGTGACCAGATGAGTTGAGAACTGGGTGGGTGGGTGCAGGCTCAATGGAAACAGAAACTCATGGATATTGACTTACATGGGAGAAAGGGGAATGGATGGGGGTCGCAGTGGGGTGGGAGGCTCCTCTTTTCCTTGTCTTTTGTTTTTTTGCCCTTCCCTCTTCTCTTACTTTTCCGTGAGGGCTCCCCAGGCTCAGGAGAGATCAGGGTGAAGGGTGACGGCCAAACCAGGGAAGGCTCCAGGTGGCTGAAGCCTGGTCTGTGCCAGCAGGGCCCTGGCGGGCTGTTCCCACTCCACCGGGCAGGTGATAACTGGTCAGAGCTGCCTCCCCACAGGTGCTGGAGGTAGGCTGGGAGGGCCGGTGCTCCCTGATGTGGACAGGGGGAGGGGTATTGATAAGAGGCGTGGAGAGATCCCTAGAGATGCAGTCTGTGGCCCCTGGGTTCCAACCCAGTGTGCCACCACCTGGCTGTGTGACCTTCAGCAAGTGCCATTATTTCTCTGAGCCTGTTTGTTTATAAAATGAGGAAGAGTTGGCACAAGTTTGAAGAATTTCTCAGGCTCCACCCGGTTCTGAAATTCCGGTAATTTCCCAACTAGGGTGCACCCCCCTGTAAGGGGCTGGGGAGGGGACGGTGCGAACCAAGTTCAACTTGTGGAGCGGAGCCAGAGCTGTTGGCCGAGCTTGGGCCTGGCCAACGCCCTGCCCTCGGGAAGGTCCTGTGTAGGGAGACTGCCTGGAGGGACTAAGCGAGGGCCTCTAACGTCTCAGGGGCAGCCTCTCAGCCTGAGACCCTGCTGGGGAAGCCGCCTCCTGTGCATAGCTGCGCCTAGGGCTGAGGTGAGGGCGCAGGCTCCCCAGGGTGTGTCCGAATTGCCCGCCTCAGCCCGCGGCCGTCCTGACCACCCAGCAGCGGCAGCAGCGACACCCTAGGGCTCCAGGAAGGGCTTGGGAAGGTGTAAGGCAGGGGCTAGGCTCGGAGGGAGCCGGAGGGACCGGGCGCAGGTTGGCTCCCGGGAGCAACTGGAGAGTGAGGAGATCCTCATCCGGGGAAGCCCCGCAGGCCGCGTCTCTACAGCGCCCTTCTCGGGTCTGGCCCTCCCGCTGGTTATTCTGGACCTGGGGGCCCCCAGCTGGGACCCGAGTCCGGTGCAGGGGAGCCTAGTGGGCCTGGGAGCTGCTATTTTTAGCGGGCGCGGCGGCGCGAGGACCTATTTATAGATCAAACAAACCGCGCTCCCTGCCTCAATGGAAACCCGCGTGCGTGACGCGCGCGCAGACATTCCAGGCCCCCCCTCCTCGCCCCGCCCCCTCGGGCTCCCCGGGCCGCACCTCCCCCTGGCCGCCTCCCGCCGGAACCGCACCGCCCCCCGCGAACCCCTTGTATGGCCAAAGCTCGACGGGCAGGCCGTGCAGTGGCACCCCGCCCTCCCTCGGTGCATCACGGAGCGCTTAAGAGGAGGGTCGGGCTCGGCCGGGGAGTCCCACTGGCGGAGGCTACGAAACTTGGGGGAGTGCACAGAAGAACTTCGGGAGCGCACGCAGGACCAGGGACCAGGCTGAGACTCGGGGCGCCAGTCCGGGCAGGGGCAGCGGGAGCCGGCCGGAG

**-1.1KB:** GGAGGGGTATTGATAAGAGGCGTGGAGAGATCCCTAGAGATGCAGTCTGTGGCCCTGGGTTCCAACCCAGTGTGCCACCACCTGGCTGTGTGACCTTCAGCAAGTGCCATTATTTCTCTGAGCCTGTTTGTTTATAAAATGAGGAAGAGTTGGCACAAGTTTGAAGAATTTCTCAGGCTCCACCCGGTTCTGAAATTCCGGTAATTTCCCAACTAGGGTGCACCCCCCTGTAAGGGGCTGGGGAGGGGACGGTGCGAACCAAGTTCAACTTGTGGAGCGGAGCCAGAGCTGTTGGCCGAGCTTGGGCCTGGCCAACGCCCTGCCCTCGGGAAGGTCCTGTGTAGGGAGACTGCCTGGAGGGACTAAGCGAGGGCTCTAACTGACGTCTCAGGGGCAGCCTCTCAGCCTGAGACCCTGCTGGGGAAGCCGCCTCCTGTGCATAGCTGCGCCCTAGGGCTGAGGTGAGGGCGCAGGCTCCCCAGGGGTGTGTCCGAATTGCCCGCCTCAGCCCGCGGCCGTCCTGACCACCCAGCAGCGGCAGCAGCGACACCCTAGGGCTCCAGGAAGGGCTTGGGAAGGTGTAAGGCAGGGGCTAGGCTCGGAGGGAGCCGGAGGGACCGGGCGCAGGTTGGCTCCCGGGAGCAACTGGAGAGTGAGGAGATCCTCATCCGGGGAAGCCCCGCAGGCCGCGTCTCTACAGCGCCCTTCTCGGGTCTGGCCCTCCCGCTGGTTATTCTGGACCTGGGGGCCCCCAGCTGGGACCCGAGTCCGGTGCAGGGGAGCCTAGTGGGCCTGGGAGCTGCTATTTTTAGCGGGCGCGGCGGCGCGAGGACCTATTTATAGATCAAACAAACCGCGCTCCCTGCCTCAATGGAAACCCGCGTGCGTGACGCGCGCGCAGACATTCCAGGCCCCCCCTCCTCGCCCCGCCCCCTCGGGCTCCCCGGGCCGCACCTCCCCCTGGCCGCCTCCCGCCGGAACCGCACCGCCCCCCGCGAACCCCTTGTATGGCCAAAGCTCGACGGGCAGGCCGTGCAGTGGCACCCCGCCCTCCCTCGGTGCATCACGGAGCGCTTAAGAGGAGGGTCGGGCTCGGCCGGGGAGTCCCACTGGCGGAGGCTACGAAACTTGGGGGAGTGCACAGAAGAACTTCGGGAGCGCACGCAGGACCAGGGACCAGGCTGAGACTCGGGGCGCCAGTCCGGGCAGGGGCAGCGGGAGCCGGCCGGAG

**-0.46KB:** GAGTCCGGTGCGGGGAGCCTAGTGGGCCTGGGAGCTGCTATTTTTAGCGGGCGCGGCGGCGCGAGGACCTATTTATAGATCAAACAAACCGCGCTCCCTGCCTCAATGGAAACCCGCGTGCGTGACGCGCGCGCAGACATTCCAGGCCCCCCCTCCTCGCCCCGCCCCCTCGGGCTCCCCGGGCCGCACCTCCCCCTGGCCGCCTCCCGCCGGAACCGCACCGCCCCCCGCGAACCCCTTGTATGGCCAAAGCTCGACGGGCAGGCCGTGCAGTGGCACCCCGCCCTCCCTCGGTGCATCACGGAGCGCTTAAGAGGAGGGTCGGGCTCGGCCGGGGAGTCCCACTGGCGGAGGCTACGAAACTTGGGGGAGTGCACAGAAGAACTTCGGGAGCGCACGCAGGACCAGGGACCAGGCTGAGACTCGGGGCGCCAGTCCGGGCAGGGGCAGCGGGAGCCGGCCGGAG

**Table S9. Nucleic acid sequences of Mutant NR4A1 Promoter.**

**WT NR4A1 Promoter:**

GGAGGGGTATTGATAAGAGGCGTGGAGAGATCCCTAGAGATGCAGTCTGTGGCCCTGGGTTCCAACCCAGTGTGCCACCACCTGGCTGTGTGACCTTCAGCAAGTGCCATTATTTCTCTGAGCCTGTTTGTTTATAAAATGAGGAAGAGTTGGCACAAGTTTGAAAAGATTTCTCAGGCTCCACCCGGTTCTGAAATTCGGTAATTTCCCAACTAGGGTGCACTCCCCCTGTAAGGGGCTGGGGAGGGGACGGTGCGAAACCAAGTTCAGCTTGTGGAGCGGAGCCAGAGCTGTTGGCCGAGCTTGGGCCTGGCCAACGCCTGCCCTCGGGAAGGTCCTGTGTAGGGAGACTGCCTGGAGGGACTAAGCGAGGGCTCTAACTGACGTCTCAGGGGCAGCCTCTCAGCCTGAGACCCTGCTGGGGAAGCCGCGTCCTGTGCACTAGCTGCGCCTAGGGCTGAGGTGAGGGCGCAGGCTCCCCAGGGTGTGTCCGAATTGCCCGCCTCAGCCCGCGGCCTGTCCTGACCGCCCAGCAGCGGCAGCAGCGACACCCTAGGGCTCCAGGAAGGGCTTGGGAAGGTGTAAAGGCGGGGCTAGGCTCGGAGGGAGCCGGAGGGACCGGGCGCGGTTGGCTCCCGGGAGCAACTGGAGAGTGAGGAGATCCTCATCCGGGGAAGCCCCGCGGCCGCGTCTCTACAGCGCCCCTTCTCGGGCTCTGGCCCTCCCGCTGGTTATTCTGGACCTGGGGGCCCCCAGCTGGGACCCGAGTCCGGTGCGGGGAGCCTAGTGGGCCTGGGAGCTGCTATTTTTAGCGGGCGCGGCGGGCGCGAGGAGCCTATTTATAGATCAAACAATCCGCGCTCCCTGCGTCAATGGAACCCCGCGTGCGTCACGCGCGCAGACATTCCAGGCCCCCCCTCCTCGCCCCGCCCCCTCGGGCTCCCCGGGCCGCACCTCCCCCTGGCCGCCTCCCGCCGGAACCGCACCGCCCCCCGCGCCCTTGTATGGCCAAAGCTCGACGGGCGGCCTGCGTCAGTGGCGCCCCCGCCCCTCCCCGTGCGTCACGGAGCGCTTAAGAGGAGGGTCGGGCTCGGCCGGGGAGTCCCAGTGGCGGAGGCTACGAAACTTGGGGGAGTGCACAGAAGAACTTCGGGAGCGCACGCGGGACCAGGGACCAGGCTGAGACTCGGGGCGCCAGTCCGGGCAGGGGCAGCGGGAGCCGGCCGGAG

**Mutant NR4A1 Promoter**

GGAGGGGTATTGATAAGAGGCGTGGAGAGATCCCTAGAGATGCAGTCTGTGGCCCTGGGTTCCAACCCAGTGTGCCACCACCTGGCTGTGTGACCTTCAGCAAGTGCCATTATTTCTCTGAGCCTGTTTGTTTATAAAATGAGGAAGAGTTGGCACAAGTTTGAAAAGATTTCTCAGGCTCCACCCGGTTCTGAAATTCGGTAATTTCCCAACTAGGGTGCACTCCCCCTGTAAGGGGCTGGGGAGGGGACGGTGCGAAACCAAGTTCAGCTTGTGGAGCGGAGCCAGAGCTGTTGGCCGAGCTTGGGCCTGGCCAACGCCTGCCCTCGGGAAGGTCCTGTGTAGGGAGACTGCCTGGAGGGACTAAGCGAGGGCTCTAACTGACGTCTCAGGGGCAGCCTCTCAGCCTGAGACCCTGCTGGGGAAGCCGCGTCCTGTGCACTAGCTGCGCCTAGGGCTGAGGTGAGGGCGCAGGCTCCCCAGGGTGTGTCCGAATTGCCCGCCTCAGCCCGCGGCCTGTCCTGACCGCCCAGCAGCGGCAGCAGCGACACCCTAGGGCTCCAGGAAGGGCTTGGGAAGGTGTAAAGGCGGGGCTAGGCTCGGAGGGAGCCGGAGGGACCGGGCGCGGTTGGCTCCCGGGAGCAACTGGAGAGTGAGGAGATCCTCATCCGGGGAAGCCCCGCGGCCGCGTCTCTACAGCGCCCCTTCTCGGGCTCTGGCCCTCCCGCTGGTTATTCTGGACCTGGGGGCCCCCAGCTGGGACCCGAGTCCGGTGCGGGGAGCCTAGTGGGCCTGGGAGCTGCTATTTTTAGCGGGCGCGGCGGGCGCGAGGAGCCTATTTATAGATCAAACAATCCGCGCTCCCCTATCAGATGGAACCCCGCGCTATCAGCGCGCGCAGACATTCCAGGCCCCCCCTCCTCGCCCCGCCCCCTCGGGCTCCCCGGGCCGCACCTCCCCCTGGCCGCCTCCCGCCGGAACCGCACCGCCCCCCGCGCCCTTGTATGGCCAAAGCTCGACGGGCGGCCCTATCAGGTGGCGCCCCCGCCCCTCCCCGCTATCAGCGGAGCGCTTAAGAGGAGGGTCGGGCTCGGCCGGGGAGTCCCAGTGGCGGAGGCTACGAAACTTGGGGGAGTGCACAGAAGAACTTCGGGAGCGCACGCGGGACCAGGGACCAGGCTGAGACTCGGGGCGCCAGTCCGGGCAGGGGCAGCGGGAGCCGGCCGGAG

**Table S10. Body weight and plasma cholesterol of GRK2^ECKO^; ApoE^-/-^ hyperlipidemia and littermate wild-type mice.**

|  | GRK2^flox/flox^; ApoE^-/-^ | GRK2^ECKO^; ApoE^-/-^ | *p* value |
| --- | --- | --- | --- |
| Body weight, g | 31.6±10.1 | 29.8±11.2 | N.S. |
| Cholesterol, mg/dl | 917.5±128.6 | 996.8±158.3 | N.S. |

Values are expressed as means ± SD, n = 12.

N.S., not significant.

**Table 11. Body weight and plasma cholesterol of high-fat Western diet fed GRK2^ECKO^; ApoE^-/-^** **mice administered** **AAV oe-AP-1^S63D^ and AAV vector.**

|  | Vector;  GRK2^ECKO^; ApoE^-/-^ | oe-AP-1^S63D^;  GRK2^ECKO^; ApoE^-/-^ | *p* value |
| --- | --- | --- | --- |
| Body weight, g | 32.8±8.9 | 30.1±11.6 | N.S. |
| Cholesterol, mg/dl | 1066.7±112.9 | 978.8±127.3 | N.S. |

Values are expressed as means ± SD.

N.S., not significant.

**Table 12. Body weight and plasma cholesterol of high-fat Western diet fed ApoE^-/-^** **mice administered AAV oe-GRK2^S29D^ and AAV vector.**

|  | Vector; ApoE^-/-^ | oe-GRK2^S29D^; ApoE^-/-^ | *p* value |
| --- | --- | --- | --- |
| Body weight, g | 30.9±9.3 | 29.1±10.1 | N.S. |
| Cholesterol, mg/dl | 992.7±106.2 | 1085.7±133.6 | N.S. |

Values are expressed as means ± SD.

N.S., not significant.
